# *Bach1* Deficiency and Accompanying Overexpression of Heme Oxygenase-1 Do Not Influence Aging or Tumorigenesis in Mice

**DOI:** 10.1155/2014/757901

**Published:** 2014-06-23

**Authors:** Kazushige Ota, Andrey Brydun, Ari Itoh-Nakadai, Jiying Sun, Kazuhiko Igarashi

**Affiliations:** ^1^Department of Biochemistry, Tohoku University Graduate School of Medicine, 2-1 Seiryo-machi, Sendai 980-0872, Japan; ^2^Division of Nephrology, Endocrinology, and Vascular Medicine, Tohoku University Graduate School of Medicine, 2-1 Seiryo-machi, Sendai 980-0872, Japan; ^3^Department of Cellular Biology, Research Institute for Radiation Biology and Medicine, Hiroshima University, Hiroshima 734-8551, Japan; ^4^Center for Regulatory Epigenome and Diseases, Tohoku University Graduate School of Medicine, 2-1 Seiryo-machi, Sendai 980-0872, Japan

## Abstract

Oxidative stress contributes to both aging and tumorigenesis. The transcription factor Bach1, a regulator of oxidative stress response, augments oxidative stress by repressing the expression of heme oxygenase-1 (HO-1) gene (*Hmox1*) and suppresses oxidative stress-induced cellular senescence by restricting the p53 transcriptional activity. Here we investigated the lifelong effects of *Bach1* deficiency on mice. *Bach1*-deficient mice showed longevity similar to wild-type mice. Although HO-1 was upregulated in the cells of *Bach1*-deficient animals, the levels of ROS in *Bach1*-deficient HSCs were comparable to those in wild-type cells. *Bach1* ^−/−^; *p53* ^−/−^ mice succumbed to spontaneous cancers as frequently as *p53*-deficient mice. *Bach1* deficiency significantly altered transcriptome in the liver of the young mice, which surprisingly became similar to that of wild-type mice during the course of aging. The transcriptome adaptation to *Bach1* deficiency may reflect how oxidative stress response is tuned upon genetic and environmental perturbations. We concluded that *Bach1* deficiency and accompanying overexpression of HO-1 did not influence aging or p53 deficiency-driven tumorigenesis. Our results suggest that it is useful to target Bach1 for acute injury responses without inducing any apparent deteriorative effect.

## 1. Introduction

Elimination of excessive reactive oxygen species (ROS) is pivotal to prevent malignant transformation and to maintain tissue homeostasis [1], because they modify DNA, lipids, and proteins, compromising their functions. According to the “free radical theory,” aging results from the accumulation of the cellular damage due to oxidative stress [2, 3]. Cells are equipped with many protective genes against ROS, among which heme oxygenase-1 (HO-1) is placed in the central position in that its expression is strongly induced in response to oxidative stress and is tightly associated with the progression of diseases involving oxidative stress [4–6]. The two beneficial functions of HO-1 have been pointed out. First, it reduces the levels of free heme, which catalyzes the production of ROS by the Fenton reaction. Second, among the products of HO-1 reaction, biliverdin and carbon monoxide (CO) mitigate ROS. Since genetic ablation of HO-1 in mice causes severe anemia, disorders of iron homeostasis, and shortening of life span, HO-1 is indispensable for organismal homeostasis [7]. Reflecting its antioxidant activities, it has been shown that the overexpression of HO-1 is protective against diverse tissue damages in disease models of mice, including disorders of heart, liver, lung, and intestine [8–11]. However, overexpression of HO-1 has also been considered to result in a pathological iron deposition and mitochondrial damage in aging-related neurodegenerative diseases [12]. Thus, whether a long-term overexpression of HO-1 would be protective or induce detrimental side effects still remains to be examined.

HO-1 is an inducible enzyme and its expression is mainly determined by the transcription level of* Hmox1* gene [13–16]. The transcription factor Bach1 forms heterodimers with small Maf oncoproteins and binds to the Maf-recognition elements (MARE) in the enhancer regions of* Hmox1* to repress its transcription [13, 17–22], whereas heterodimers composed of Nrf2 and small Maf oncoproteins bind to the same sequences to activate* Hmox1* [21, 23–25]. In* Bach1*-deficient mice, the levels of HO-1 are higher compared with control mice in many tissues, including the liver, indicating that Bach1 is a physiological repressor of* Hmox1* [18]. The protective effect of HO-1 appears to be constrained by Bach1 under several specific disease conditions, because* Bach1*-deficient mice are more resistant to tissue damage than wild-type mice in the models of lung, liver, intestine, and cardiovascular diseases [26–31]. Another function of the Bach1-HO-1 axis resides in the regulation of innate immunity [32]. Particularly, the Bach1-HO-1 axis is important for the proper function of the antigen presenting cells such as macrophages and dendritic cells [32].

Recently we have reported that Bach1 restricts the implementation of cellular senescence transcription program in mouse embryonic fibroblasts (MEFs) [33]. Cellular senescence is induced by ROS through DNA damage responses and acts as a barrier against malignant transformation of damaged cells [34, 35]. The tumor suppressor p53 induces cellular senescence in response to oxidative stress, oncogenic stress, and direct DNA damage [36]. Bach1 represses p53-mediated cellular senescence by forming a complex with p53, recruiting histone deacetylase-1 (HDAC1) and thereby repressing a subset of p53 target genes through histone deacetylation [33, 37]. Bach1 specifically inhibits oxidative stress-induced p53-dependent cellular senescence [33]. The p53-Bach1 interaction is inhibited by the tumor suppressor p19^ARF^ [38], which is consistent with the senescence-restricting function of Bach1. Furthermore,* Bach1*-deficient MEFs are resistant to transformation by activated H-Ras oncogene, which is known to utilize increased levels of ROS for transformation [39]. Therefore, a reduction in the Bach1 activity may lead to an enhancement in the tumor-resistant phenotype. However, the roles of Bach1 in tumorigenesis are controversial. There are several reports suggesting that increased levels of HO-1 are associated with tumorigenesis [40–43]. An oncogenic microRNA, miR-155, targets Bach1 and their interaction has been discussed in the context of leukemogenesis [44].

Despite the growing number of evidences that inhibition of Bach1 may be beneficial in certain clinical situations [1, 26, 28, 29, 31, 45], the long-term, organismal response to* Bach1* ablation has not yet been examined. Considering its distinct effects on ROS homeostasis and cell proliferation,* Bach1* deficiency may affect the aging and/or life span in mice under normal conditions; ROS levels would decrease via the derepression of HO-1, and/or the p53-dependent cellular senescence would be increased by the enhanced activity of p53. The purpose of this study was to determine the lifelong effects of* Bach1* deficiency on mice. We monitored cohorts of mice under typical laboratory conditions. We also generated* Bach1*;* p53*-double deficient mice to examine whether the* Bach1* deficiency and accompanying overexpression of HO-1 would affect tumor incidence in the absence of the main tumor suppressor p53. To investigate the possibility that some of the effects of* Bach1* deficiency might be compensated for, we carried out an expression profiling of the liver, a major organ of the iron/heme metabolism, during aging. The overall results indicated that Bach1 was not necessary for the normal life course of mice, including longevity and tumorigenesis under the laboratory conditions. The apparent normal phenotypes of* Bach1*-deficient mice involved a transcriptome-wide adaptation in the liver induced upon aging, which shows a novel gene regulatory mechanism compensating for the loss of Bach1. Based on our results, we discuss possible application of Bach1 inhibition for a clinical treatment.

## 2. Material and Methods

### 2.1. Mice

*Bach1*-deficient mice were previously reported [18] and back-crossed to C57B6J at least 12 times.* p53*-deficient mice with C57B6J background were provided by Dr. Motoya Katsuki. Mice were fed* ad libitum.*

### 2.2. Genotyping

Four-week-old mice were used for genotype. Mouse DNA was extracted as previously reported [18]. PCRs were carried out using the following primers: Bach1-F 5′-CATGTGTGTTTGCAGGTCGA-3′, Bach1 mutant-F 5′-AGTAGGTGTCATTCTATTCTGGG-3′, Bach1-R 5′-GTGGAAGTAGCTGCTGCACG-3′, p53-F 5′-CACCTGCACAAGCGCCTCTC-3′, and p53-R 5′-GCTGTCTCCAGACTCCTCTG-3′. PCR product of p53 amplicon was digested by EcoRI to discriminate the genotypes.

### 2.3. Antibodies for Flow Cytometry

The following fluorescent dye-conjugated monoclonal antibodies were purchased from BD Bioscience (Franklin Lakes, NJ) and used for flow cytometry and cell sorting (FACSCantoII or FACSAriaII): Sca-1 (D7), c-kit (2B8), Flt-3 (A2F10), and CD150 (TC15-12F12.2). Biotinylated lineage antibodies CD3 (145-2C11), CD4 (GK1.5), CD8a (53-6.7), CD11b (M1/70), B220 (RA3-6B2), Gr-1 (RB6-8C5), NK1.1 (PK136), and Ter119 were revealed with streptavidin-PerCP or streptavidin-PerCP5.5.

### 2.4. Analysis of Intercellular ROS in HSCs

Bone marrow cells were collected by aspiration and 5 × 10^6^ cells were stained with PE-Flt3, APC-Sca-1, APC-Cy7-c-kit, and PerCP-lineage antibodies. The cells were incubated with 5 *μ*M DCF-DA (Sigma) in 10% FBS IMDM (Gibco) at 37°C for 30 min and then analyzed by FACSCantoll (BD Bioscience) (52).

### 2.5. Gene Expression Profiling and Real-Time qPCR

All equipment and reagents used for the gene expression profiling were purchased from Agilent Technologies (Santa Clara, CA). Total RNA samples were isolated, amplified, labeled with cyanine-3 dye, and hybridized with Whole Mouse Genome Array (4 × 44 K) slides exactly following the manufacturer's protocol. The analyses were carried out in triplicate. Genes expression data were analyzed using Genespring GX 12 (Agilent Technologies) and IPA 8 (Ingenuity Systems, Redwood City, CA) softwares. The difference in the genes expression was assessed by Volcano plots using moderated* t*-test with Benjamini-Hochberg FDR correction (*α* = 0.05), and 1.5-fold difference with corrected *P* value <0.05 was considered significant. For pathway analysis and GO semantic analysis, enrichment with corrected *P* value <0.0001 (Fisher's exact test, Bonferroni FDR correction) was considered significant. qPCRs were performed using a Light Cycler 2.0 instrument in the SYBR green format (Roche Diagnostics, Mannheim, Germany). Expression of *β* *-actin* was used as an internal control. The primer sequences for *β* *-actin* and* Alas1* were published previously [46, 47].

### 2.6. Histological Analysis

The sections of formalin fixed paraffin embedded tissue samples were stained with hematoxylin and eosin or Prussian blue dye. The specimens were examined by an experienced pathologist.

### 2.7. Bone Marrow Transplantation

The 8-week-old CD45.1 congenic C57/B6 mice were subjected to lethal *γ*-irradiation in two doses of 500 rads each (for a total of 1000 rads) with 3 hours blank time and were injected with 100 sorted LT-HSCs (Lin^−^, c-Kit^+^, Sca-1^+^, Flt3^−^, and CD150^+^) from wild-type or* Bach1*-deficient CD45.2 mice, in competition with 2 × 10^5^ bone marrow mononuclear cells from CD45.1 and CD45.2 heteromice via a tail vein. Mice were treated with ampicillin (30 mg/mL) in the drinking water for 4 weeks. Peripheral blood cells (PBs) reconstitution by donor cells was monitored every 4 weeks. PBs were stained with fluorescent-conjugated antibodies specific for CD45.1, CD45.2, CD4, CD8, CD11b, Gr-1, and B220 and analyzed by a flow cytometry.

### 2.8. Statistical Analysis

Quantitative data except for the genes expression analysis were evaluated using JMP 10 (SAS Institute, Inc., Cary, NC). Log-rank test was used for analysis of survival curves. Student's* t*-test with Welch's correction was used for analysis of measurement of ROS level and bone marrow transplantation experiments. Fisher's exact test was used for comparison of death rates among* Bach1*;* p53* mutant mice. *P* values less than 0.05 were considered statistically significant in all tests.

## 3. Results and Discussion

### 3.1. Life Span of* Bach1*-Deficient Mice

**Figure 1:**
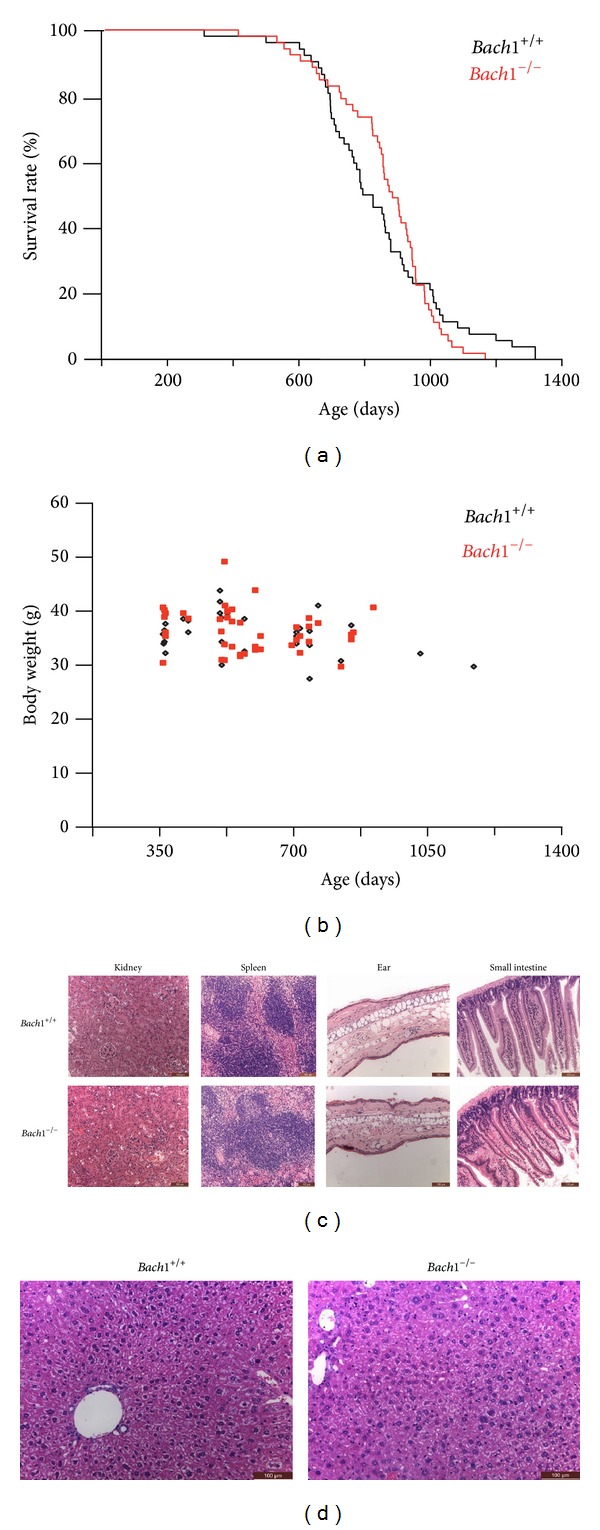
Effects of* Bach1* deficiency on life span. (a) 52 wild-type and 53* Bach1*-deficient mice were monitored for their lives. The average life span was 846.1 ± 199.3 days and 867.5 ± 154.0 days, respectively. (b) The body weight of wild-type and* Bach1*-deficient mice is plotted against the age. (c) The histology of kidney, spleen, skin of ear, and small intestine in wild-type and* Bach1*-deficient mice. Representative images are shown among three mice of each genotype. Scale bars are 100 *μ*m. (d) The histology of liver in wild-type and* Bach1*-deficient mice. Representative images are shown among three mice of each genotype. Scale bars are 100 *μ*m.

As reported previously [18],* Bach1*-deficient mice were born according to Mendelian ratio with normal fertility being indistinguishable from wild-type mice. To examine the effect of* Bach1* deficiency during the life span, cohorts of* Bach1*-deficient and wild-type mice were followed up under pathogen-free condition. Life span was not affected (log-rank test, *P* = 0.93) by* Bach1* deficiency (Figure 1(a)). We used body weight as a basic indicator of mice health and general condition. Logistic regression model indicated that age (*P* = 0.045) and sex (*P* < 0.0001) but not genotype (*P* = 0.45) were significantly associated with body weight (Figure 1(b)). No difference was found by the histological analysis of major organs such as kidney and spleen in around 100-week-old mice (Figure 1(c)). The thickness of skin from ear and villi of small intestine in aging* Bach1*-deficient mice were similar to those of wild-type mice (Figure 1(c)). Because HO-1 liberates iron from heme, we expected that* Bach1*-deficient mice would show enhanced iron deposition. However, there was no apparent change in morphology, iron deposits, and concentration of heme in the liver of aging* Bach1*-deficient mice compared with those in wild-type mice (Figure 1(d)). Our results demonstrated that* Bach1* deficiency did not affect the phenotypes of aging and life span.

### 3.2. Effect of* Bach1* Deficiency on Hematopoietic Stem Cells

**Figure 2:**
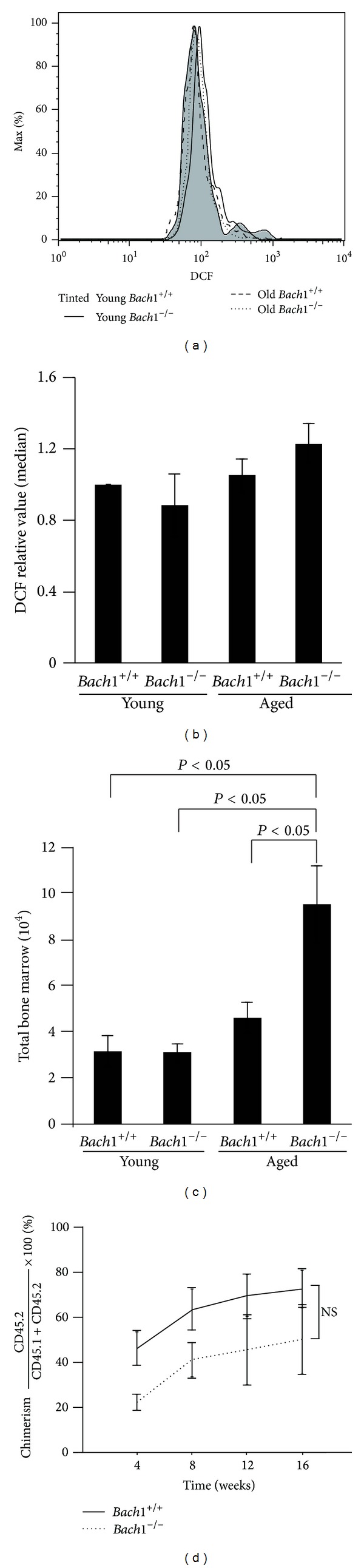
Effects of* Bach1* deficiency in HSCs. (a) The levels of oxidized DCFDA in HSCs in young and aged, wild-type and* Bach1*-deficient mice. A representative result from three independent experiments is shown. (b) The averages of median of the experiments in the three experiments of (a). The results are expressed as mean ± S.D (*n* = 3); relative values to young wild-type HSCs in each experiment. (c) The numbers of HSCs in young and aged, wild-type and* Bach1*-deficient mice. The results are expressed as mean ± S.D (*n* = 3). *P < 0.05. (d) Transplantations of bone marrow LT-HSCs between wild-type and* Bach1*-deficient mice. The chimerism data from five wild-type and four* Bach1*-deficient mice are presented as means ± S.E.M. The chimerism was calculated by a formula, CD45.2 × 100/(CD45.1 + CD45.2) (%).

We evaluated* Bach1*-deficient HSCs since these cells are sensitive to increased levels of ROS and become senescent under such conditions [48, 49]. HSCs isolated from* Bach1*-deficient and wild-type mice (around 10 and 100 weeks) were stained with DCFDA, which was converted to fluorescent DCF within cells by ROS including hydrogen peroxide (Figure 2(a)). To quantify levels of fluorescence, we showed the averages of median in the three independent experiments (Figure 2(b)). The levels of ROS were similar irrespective of the genotype and age of the mice, indicating that* Bach1* deficiency did not apparently influence the metabolism of ROS in the HSCs. While those in young* Bach1*-deficient mice were comparable to wild-type mice, the numbers of HSCs in aged* Bach1*-deficient mice increased (Figure 2(c)). Therefore, it is speculated that Bach1 would affect proliferation and/or maintenance of HSCs during aging. To more directly assess the function of HSCs, transplantations of young wild-type or* Bach1*-deficient LT-HSCs to lethally irradiated wild-type mice were performed (Figure 2(b)). No obvious difference in the chimerism was observed irrespective of the genotypes. These data demonstrated that* Bach1* deficiency did not apparently affect ROS metabolism and the reconstitution activity of HSCs under the normal physiological condition.

We conclude that cellular senescence and its associated phenotype were not accelerated to a degree affecting organismal soundness in* Bach1*-deficient mice kept under the nonstressed condition. We need to examine whether* Bach1* deficiency modulates ROS metabolism when mice are challenged with oxidative stress. The results suggest that a targeting of Bach1 in medical treatment is acceptable for acute and chronic injuries without any long-term side effect as shown in several papers [1, 26, 28, 29, 31, 45].

### 3.3. Effect of* Bach1* Deficiency on Tumor Formation in the p53-Null Mice

*Bach1* deficiency in fibroblasts leads to the resistance to transformation by the H-Ras^V12^ oncogene, possibly by means of mitigating ROS accumulation induced by H-Ras^V12^. Also* Bach1*-deficient mice are less susceptible to 4-nitroquinoline-1-oxide- (4-NQO-) induced tongue carcinoma than wild-type mice [39]. These observations suggest that higher HO-1 levels may be causal for the tumor-suppressive phenotype of* Bach1* deficiency in this experimental model. On the other hand, it has recently been reported that an overexpression of HO-1 is associated with certain types of cancers [40–43, 50]. Therefore, we examined whether Bach1 or its absence would contribute to spontaneous tumorigenesis. We generated cohorts of* Bach1*-deficient mice and* Bach1-* and* p53-*double deficient (*Bach1* ^−/−^;* p53* ^−/−^) mice because* p53* deficiency induces intrinsic onset of tumorigenesis [51, 52]. All of the combinatorial genotypes were viable and indistinguishable to one another. The absence of obvious genetic interaction between Bach1 and p53 during embryonic development and neonatal period was not contradictory to our previous report that Bach1 inhibited p53 [33] and showed that their combined effects could be assessed in the tumorigenesis at adult stages.

*p53*-deficient mice die earlier than wild-type ones due to frequent tumor formation [51, 52]. Mice of various genotypes including* Bach1* ^−/−^;* p53* ^−/−^ were followed up for thirty weeks during which most of* p53*-deficient mice succumbed to cancers (Table 1). While all of the wild-type mice were alive by the end of observation, many of the* p53*-deficient mice died due to spontaneously developed tumors.* Bach1* deficiency did not apparently reduce the death rate in* p53*-deficient mice (Fisher's exact test; *P* = 0.6).

Considering functions of Bach1, we need to clarify the possible involvement of Bach1 and HO-1 in specific tumors by using of different tumor initiation models. Since cellular senescence would not occur in the absence of p53, Bach1 may not affect transformation under the experimental conditions we used. Likewise, the involvement of HO-1 in cancer may depend on genetic changes underlying the transformation process [40–43, 50]. It should be noted that HO-1 can accelerate tumor growth and affect malignancy of the tumor [53]. Hence, we need to assess impacts of* Bach1* deficiency in terms of tumor incidence, spread, and progression as well. Notwithstanding these remaining issues, our current observations indicate that Bach1 does not play a critical modifying role in incidence of death due to tumor in the context of* p53*-null condition.

### 3.4. Transcriptome-Wide Adaptation in* Bach1*-Deficient Mice

**Figure 3:**
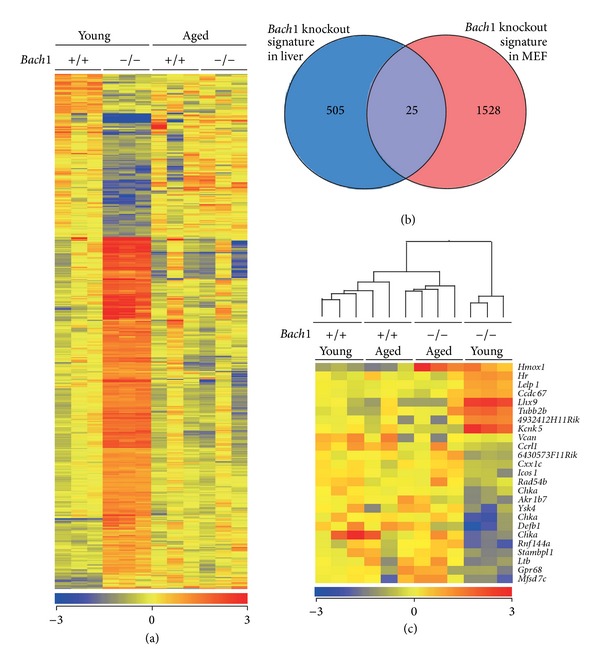
Effects of age and* Bach1* deficiency on gene expressions in mice. (a) Heat map visualizations of the 530 entities, differentially expressed in the livers of young* Bach1-*deficient compared with young wild-type mice. Three mice were used for each group. Here and elsewhere the data for heat maps are normalized and represented as median-centered log-transformed values, using average linkage clustering on entities. Red and blue correspond to high and low expression, respectively, compared with the experiment-wide median. (b) Venn diagram shows overlap between the* Bach1* knockout gene expression signature in liver and the previously published [39]* Bach1* knockout signature in MEF. (c) Heat map visualizations of the 25 entities common for both signatures. Hierarchical clustering on entities and conditions.

One possible explanation for the above observations was that the loss of Bach1 was compensated by other genes and/or pathways. To address this possibility, we focused on gene expression profiles as an intermediary trait. Liver samples were used for this analysis since it is one of the major organs for the metabolism of heme, which is a ligand of Bach1 [19, 54]. We compared the gene expression profiles of the livers of wild-type and* Bach1*-deficient mice along the course of aging. We identified a* Bach1* knockout signature comprised of 530 entities (1.7% of 31433 examined) which were differentially expressed in the livers of 8-week-old* Bach1*-deficient mice compared with wild-type mice of the same age (Figure 3(a) and see Supplementary Table 1 in Supplementary Material available online at  http://dx.doi.org/10.1155/2014/757901). Interestingly, the genes expression signature of the* Bach1*-deficient livers disappeared when examined at 100 weeks of age (Figure 3(a)). The 530 gene sets showed overlap with previously published Bach1 knockout signature in MEFs (25 gene entities, Figure 3(b)) [39]. To our surprise, hierarchical clustering of the 25 entities common for both signatures demonstrated that all genes except for* Hmox1* returned to the normal levels similar to those in wild-type livers (Figure 3(c)). While the expression of Bach1-regulated genes is deregulated in the absence of Bach1 in the liver, their expression can be modulated to a normal pattern upon aging.

**Figure 4:**
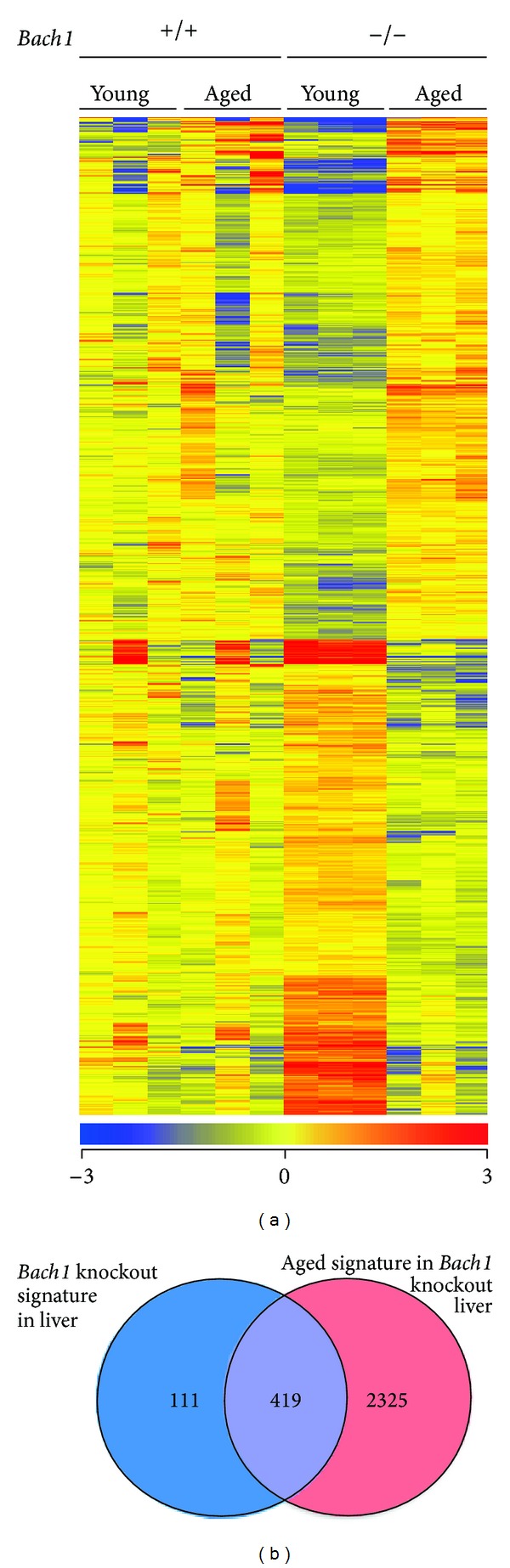
The effect of aging on genes expression profile in* Bach1*-deficient mice liver. (a) Heat map visualizations of the 2744 entities, differentially expressed in the livers of young* Bach1-*deficient mice compared with aged ones. (b) Venn diagram shows overlap between the* Bach1* knockout genes expression signatures (Figure 3(b)) and aging signature in* Bach1*-deficient liver.

To understand which part of the transcriptome was affected in young* Bach1*-deficient mice and resolved with age, we analyzed the aging signature in wild-type and* Bach1*-deficient animals. A relatively small number of genes are known to be affected by aging in mouse liver [55]. There was no statistically significant difference in genes expression in the livers of the aged wild-type mice compared with those of young wild-type mice. In contrast, expression of 2744 entities (8.7% of 31433 examined) was significantly altered by aging in* Bach1*-deficient livers (Figure 4(a)). Roughly half of them were upregulated with aging, whereas the other half was repressed upon aging (Figure 4(a) and Supplementary Table 2). The set of genes repressed in the aged* Bach1*-deficient livers could include direct transcriptional target of Bach1. Their reductions in the expression levels upon aging suggest that the activities of transcription factors responsible for their expression may recede upon aging. On the other hand, the genes which were induced upon aging in* Bach1*-deficient livers may represent indirect target genes of Bach1 and may require specific activating signals that set off upon aging. Approximately 80% of the genes which were differentially expressed in young wild-type and* Bach1*-deficient mice and then reverted to the normal levels (Figure 3(a)) belonged to the genes altered upon aging in* Bach1*-deficient mice (Figure 4(b)), indicating that the genes induced or reduced during aging in* Bach1*-deficient mice may compensate for the* Bach1* deficiency and may contribute to the apparent lack of any phenotypic alteration in the long term.

**Figure 5:**
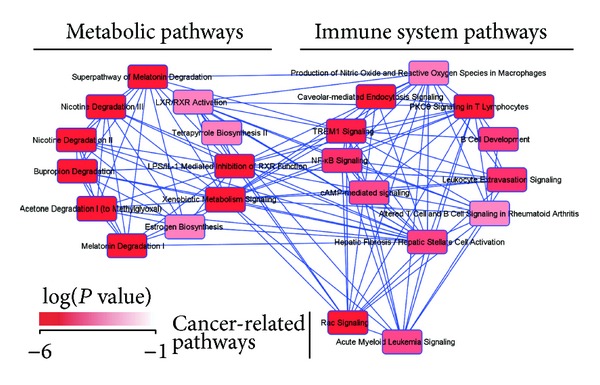
Pathway analysis of the genes which were affected by aging in the livers of* Bach1*-deficient mice. Network presentation of the canonical pathways, components of which were significantly enriched in the aging signature of* Bach1*-deficient mice liver. The intensity of red nodes color indicates the degree of significance (Fisher's exact test). The lines indicate overlap between pathways.

**Figure 6:**
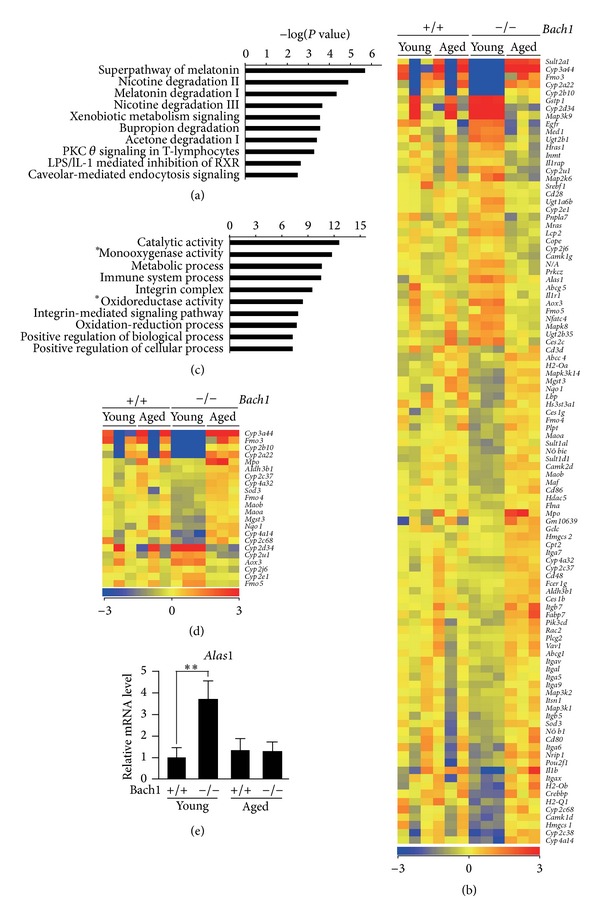
Semantic analysis of the genes which were affected by aging in the livers of* Bach1*-deficient mice. (a) Ten most significantly enriched canonical pathways are listed in significance order (Fisher's exact test). (b) Heat map visualization of the genes from ten most significantly enriched canonical pathways. (c) GO analysis of the genes from ten most significantly enriched canonical pathways. The GO terms are listed in enrichment significance order (Fisher's exact test). (d) Heat map visualizations of the genes corresponding to the GO terms *monooxygenase activity and *oxidoreductase activity in the panel (c). (e) mRNA levels of* Alas1* normalized with beta-*actin*. The results are expressed as mean ± S.D (*n* = 3). **P < 0.05.

To understand the relationship between the transcriptome alterations in* Bach1*-deficient livers and organism functions, we performed pathway analysis (IPA software, Ingenuity Systems) on the list of entities expression of which was significantly altered by aging in* Bach1*-deficient mice (Figure 4(a) and Supplementary Table 2). We identified two clearly distinguished but interconnected modules in the gene regulation network: metabolic pathways module and immune system signaling module, the latter of which also included two tumor-related pathways (Figure 5). Among the significantly enriched pathways, the highest enrichment scores were associated with metabolic pathways (Figure 6(a); genes are listed in Figure 6(b)). These genes showed little change in wild-type mice but showed either increases or decreases in* Bach1*-deficient mice upon aging (Figure 6(b)). Semantic analysis of these genes and their functions revealed significant enrichment of the genes responsible for catalytic activity of oxidoreductases including cytochrome P450 monooxygenase system (Figure 6(c)). A significant portion of oxidoreductases genes were downregulated in the liver of young* Bach1*-deficient mice compared with those of young wild-type mice, and this alteration disappeared upon aging (Figure 6(d)). Many of the oxidoreductases genes encode hemoproteins. Remarkably, we found that expression of* Alas1*, the rate-limiting enzyme in the heme synthetic pathway, was upregulated in the liver tissue of the young* Bach1*-deficient mice (Figure 6(e)). The heme biosynthesis pathway may be activated in* Bach1*-deficient mice when they are young, compensating for the higher expression of HO-1 and therefore enhanced degradation of heme. It has been reported previously that the enzymatic activity of Alas1 decreases upon aging in several tissues including the liver in rats [56]. While this particular paper assessed the enzymatic activity of Alas using aging rats, their results suggest that the expression of* Alas1* mRNA would decrease upon aging [56]. While we did not observe such an alteration in the wild-type mice,* Alas1* mRNA indeed decreased upon aging in* Bach1*-deficient mice. Genes involved in aging may affect the expression of* Alas1* and such a response may have been easily observed in* Bach1*-deficient liver cells due to the higher expression of* Alas1* mRNA in the young* Bach1*-deficient mice compared with corresponding wild-type mice. The reduction of* Alas1* mRNA may indirectly facilitate the transcriptome adaptation in* Bach1*-deficient mice by altering the heme synthesis.

The observed transcriptome-wide adaptation in the gene regulatory network upon aging may be one possible mechanism to suppress a long-term effect of* Bach1* deficiency and HO-1 overexpression. Any side effect of HO-1 overexpression in the absence of Bach1 could be avoided by the transcriptome adaptation. Considering that Bach1 is primarily a repressor of gene expression, the activities of activating transcription factors such as Nrf2 may affect the expression levels of downstream genes in the absence of Bach1. Upon aging, such activators may be inhibited, resulting in a stable and harmless transcriptome. This hypothesis posits that ROS levels would decrease in the liver upon aging. However, we did not see any significant change in the levels of ROS in HSCs upon aging. Since the metabolism of ROS may be differentially regulated upon aging depending on tissues, we need to compare ROS levels in the liver as well.

## 4. Conclusions

Here we showed that Bach1 did not affect phenotypes of aging or life span of mice. At the transcriptome level,* Bach1* deficiency itself affected expression of many genes, including* Hmox1*, cytochrome P450 monooxygenase system, and* Alas1*. However, the difference in the expression of all these genes except for* Hmox1* disappeared when the mice aged. Our findings suggest that long-term targeting of* Bach1*, retaining high expression levels of* Hmox1* during the life span, is safe and free from adverse effects owing to compensation at the level of transcriptome. However, although we did not observe any detrimental effect of* Bach1* deficiency and the long-term overexpression of HO-1 under the normal conditions, it remains possible that Bach1 becomes critical under specific conditions. This is a rational inference considering its evolutionary conservation among vertebrates [22].

## Supplementary Material

Supplement Table 1: Upregulated and downregulated genes in the livers of 8-week-old Bach1-deficient mice compared with wild-type mice of the same age.
Supplement Table 2: Upregulated and downregulated genes in the livers of aged Bach1-deficient mice compared with 8-week-old Bach1-deficient mice.

## Figures and Tables

**Table 1:** Death rate before age of thirty weeks^a^.

Genotype	*p53 *		
	+/+	+/−	−/−
*Bach1 *			
+/+	0/2	0/4	7/10
+/−	0/11	0/19	6/11
−/−	0/9	1/9	2/5

^a^The denominator is the number of observed littermates and the numerator is the number of dead littermates born from *Bach1* ^+/−^; *p53* ^+/−^ parents (*n* = 80).

## Abbreviations

4-NQO: 4-Nitroquinoline-1-oxide
Bach1: BTB and CNC homology 1
CO: Carbon monoxide
DCFDA: Dichlorodihydrofluorescein diacetate
HDAC1: Histone deacetylase 1
HSCs: Hematopoietic stem cells
HO-1: Heme oxygenase-1
MARE: Maf-recognition element
MEFs: Mouse embryonic fibroblasts
PBs: Peripheral blood cells
ROS: Reactive oxygen species.
