# Supplementary material for: Bach1 Deficiency and Accompanying Overexpression of Heme Oxygenase-1 Do Not Influence Aging or Tumorigenesis in Mice
Source: Oxid Med Cell Longev. 2014 Jun 23;2014:757901. doi: 10.1155/2014/757901 (PMC4094857; doi:10.1155/2014/757901)
Supplement: Supplementary file 1 — Supplement Table 1: Upregulated and downregulated genes in the livers of 8-week-old Bach1-deficient mice compared with wild-type mice of the same age. Supplement Table 2: Upregulated and downregulated genes in the livers of aged Bach1-deficient mice compared with 8-week-old Bach1-deficient mice. [file 757901.f1.pdf]

Supplementary table I. Bach1 knockout signature in mice liver

Upregulated entities

Agilent probe ID	Fold change	p-value (Corr)	Gene Symbol	Genbank Accession
A_55_P2044653	32.6	2.2.E-02	*Cyp2b10*	NM_009999
A_55_P2408588	20.9	2.1.E-02	*Arntl*	NM_007489
A_55_P2059931	12.4	2.1.E-02	*Prom1*	NM_001163577
A_52_P329367	9.7	4.3.E-02	*Chka*	NM_001025566
A_55_P2044143	9.7	3.8.E-02	*Loxl4*	NM_001164311
A_52_P321140	8.3	3.5.E-02	*Defb1*	NM_007843
A_55_P1962516	8.1	2.1.E-02	*Fam19a2*	NM_182807
A_55_P1980214	7.2	2.2.E-02	*N/A*	N/A
A_51_P480119	6.8	2.1.E-02	*Prelid2*	NM_029942
A_66_P124179	6.8	3.8.E-02	*Atp6v0d2*	NM_175406
A_55_P2030524	6.8	4.9.E-02	*Vldlr*	NM_013703
A_55_P2020072	6.7	2.3.E-02	*N/A*	N/A
A_55_P2156062	6.3	3.7.E-02	*Pick1*	AK090155
A_55_P2429480	6.1	2.3.E-02	*Atad2*	NM_027435
A_51_P362066	5.9	3.7.E-02	*Chi3l1*	NM_007695
A_51_P440047	5.8	3.2.E-02	*1110067D22Rik*	NM_173752
A_51_P248666	5.6	3.2.E-02	*Cd274*	NM_021893
A_52_P48681	5.4	3.1.E-02	*Cldn1*	NM_016674
A_51_P142923	5.4	3.2.E-02	*Chka*	NM_013490
A_55_P2111508	5.4	2.5.E-02	*Ttyh1*	NM_021324
A_55_P2072005	5.3	3.6.E-02	*Ctsc*	AK152524
A_55_P2030938	5.0	2.9.E-02	*Trim59*	NM_025863
A_55_P1962224	5.0	3.5.E-02	*Afap1l2*	NM_146102
A_55_P2091191	5.0	3.0.E-02	*Slc28a2*	NM_172980
A_55_P2214348	4.8	3.9.E-02	*4930469K13Rik*	AK037310
A_51_P257675	4.8	5.0.E-02	*Tspyl4*	NM_030203
A_52_P140005	4.7	2.7.E-02	*Nipal1*	NM_001081205
A_66_P114381	4.7	2.1.E-02	*Ypel2*	NM_001005341
A_52_P350537	4.6	2.7.E-02	*Mtmr11*	NM_181409
A_51_P423709	4.5	3.4.E-02	*Fam84a*	NM_029007
A_55_P1964871	4.4	2.1.E-02	*Gm5549*	XM_485313
A_55_P2072771	4.4	4.1.E-02	*Cd3g*	NM_009850
A_52_P402786	4.3	4.3.E-02	*Prom1*	NM_008935
A_51_P286748	4.3	3.8.E-02	*Frzb*	NM_011356
A_51_P467448	4.2	2.6.E-02	*Pif1*	NM_172453
A_51_P247637	4.1	3.2.E-02	*Rnf144a*	NM_080563
A_55_P2161055	4.0	2.1.E-02	*Trp63*	NM_001127261

A_51_P488196	4.0	2.3.E-02	*Bmper*	NM_028472
A_55_P1965836	3.9	2.1.E-02	*Crebbp*	NM_001025432
A_51_P424810	3.9	4.1.E-02	*Ncapg2*	NM_133762
A_55_P2059010	3.8	3.2.E-02	*Rbp1*	NM_011254
A_55_P2090874	3.8	4.5.E-02	*Ysk4*	NM_011737
A_55_P2177911	3.8	4.7.E-02	*Lepr*	NM_010704
A_55_P1983152	3.8	4.3.E-02	*Gramd4*	NM_001205353
A_55_P2073377	3.8	3.5.E-02	*Mki67*	NM_001081117
A_52_P54280	3.7	4.3.E-02	*Adck3*	NM_023341
A_55_P2112360	3.7	2.3.E-02	*Arhgap17*	NM_144529
A_51_P386899	3.7	2.3.E-02	*Mfsd7c*	NM_145447
A_51_P304683	3.6	3.8.E-02	*Clpx*	NM_011802
A_55_P1955637	3.6	4.9.E-02	*Trp53i11*	NM_001025246
A_55_P2039274	3.5	3.8.E-02	*Hspb3*	NM_019960
A_51_P314186	3.5	3.1.E-02	*Syne1*	NM_001079686
A_55_P1953723	3.5	4.9.E-02	*Rab13*	AK080805
A_52_P488437	3.5	2.1.E-02	*Plek2*	NM_013738
A_55_P2107207	3.4	2.3.E-02	*Gpr137b*	AK009736
A_51_P267354	3.4	3.1.E-02	*Lrfn3*	NM_175478
A_55_P1954231	3.3	4.2.E-02	*Lrtm2*	NM_172492
A_55_P2072493	3.3	2.1.E-02	*F8*	NM_001161374
A_55_P2102350	3.3	3.5.E-02	*Stambpl1*	NM_029682
A_55_P2152427	3.3	4.3.E-02	*Zwilch*	NM_026507
A_55_P2105152	3.3	4.2.E-02	*Trim59*	NM_025863
A_51_P253803	3.3	4.2.E-02	*Mki67*	NM_001081117
A_55_P2095103	3.2	4.3.E-02	*Gucy1a2*	NM_001033322
A_51_P405606	3.2	3.2.E-02	*Ndrg1*	NM_008681
A_55_P1954798	3.2	3.7.E-02	*Gm15292*	NM_001177487
A_55_P2119772	3.2	3.9.E-02	*Scn3a*	NM_018732
A_52_P131353	3.2	4.4.E-02	*Camk1d*	NM_177343
A_52_P102248	3.2	3.1.E-02	*Mex3b*	NM_175366
A_66_P111191	3.2	4.0.E-02	*N/A*	Z48592
A_55_P2351505	3.2	3.7.E-02	*Dhx9*	NM_007842
A_51_P331288	3.1	2.1.E-02	*Akr1b7*	NM_009731
A_52_P354373	3.1	2.1.E-02	*1190002F15Rik*	NR_037956
A_51_P415546	3.1	3.5.E-02	*Defb6*	NM_054074
A_55_P2128869	3.1	3.2.E-02	*Ccdc80*	NM_026439
A_55_P2448776	3.1	3.3.E-02	*Mdn1*	NM_001081392
A_52_P89477	3.0	4.4.E-02	*Bcl9*	AK147659
A_55_P2110245	3.0	4.1.E-02	*Fignl1*	NM_001163359
A_55_P2156524	3.0	4.2.E-02	*N/A*	N/A

A_55_P1983773	3.0	2.3.E-02	*Birc5*	NM_001012273
A_51_P144349	3.0	3.6.E-02	*Dtx4*	NM_172442
A_55_P1992247	3.0	4.1.E-02	*Rergl*	NM_001128090
A_55_P2316041	3.0	2.7.E-02	*N/A*	BC050972
A_55_P2251974	3.0	3.2.E-02	*A930038B10Rik*	AK044736
A_55_P2103026	3.0	4.9.E-02	*Sema3d*	NM_028882
A_55_P2045741	3.0	4.2.E-02	*Fyb*	NM_011815
A_55_P1980636	2.9	4.3.E-02	*Aurka*	NM_011497
A_55_P2003483	2.9	3.4.E-02	*Gldc*	NM_138595
A_55_P2133225	2.9	2.1.E-02	*Mmgt2*	NM_175002
A_51_P352594	2.9	2.5.E-02	*St5*	NM_001001326
A_51_P302358	2.8	2.3.E-02	*Ltb*	NM_008518
A_55_P2080956	2.8	4.1.E-02	*Chka*	NM_013490
A_52_P476731	2.8	4.1.E-02	*Fam110c*	NM_027828
A_55_P1967500	2.8	4.6.E-02	*Nell1*	NM_001037906
A_55_P2061779	2.8	2.8.E-02	*Zfp456*	NM_001001186
A_52_P354682	2.8	3.2.E-02	*Elovl7*	NM_029001
A_55_P2033041	2.8	4.9.E-02	*Sirpb1b*	NM_001173460
A_66_P139683	2.7	2.3.E-02	*Zbp1*	NM_021394
A_52_P186937	2.7	2.4.E-02	*Cmpk2*	NM_020557
A_52_P235861	2.7	4.1.E-02	*Peg3*	NM_008817
A_51_P391454	2.7	3.0.E-02	*Il7*	NM_008371
A_55_P1956083	2.7	3.0.E-02	*Gpr68*	NM_175493
A_55_P2169064	2.7	4.2.E-02	*Olfr1474*	NM_001011842
A_51_P314397	2.7	3.9.E-02	*Crip2*	NM_024223
A_55_P2050226	2.6	3.3.E-02	*Ccrl1*	AY072938
A_55_P1985070	2.6	4.6.E-02	*Robo1*	NM_019413
A_55_P2098598	2.6	2.3.E-02	*Btg1*	NM_007569
A_55_P2102624	2.6	3.4.E-02	*Eaf2*	NM_001113401
A_52_P1076124	2.6	4.1.E-02	*N/A*	AK053814
A_55_P1983883	2.5	4.8.E-02	*Vps37a*	NM_033560
A_51_P228295	2.5	2.3.E-02	*Mpzl1*	NM_001001880
A_52_P534583	2.5	4.7.E-02	*Ahsp*	NM_133245
A_51_P520966	2.5	4.4.E-02	*Icosl*	NM_015790
A_55_P1960197	2.5	3.1.E-02	*P2ry14*	NM_133200
A_55_P2108784	2.5	4.2.E-02	*Arhgap22*	NM_153800
A_51_P199135	2.5	3.8.E-02	*Cd83*	NM_009856
A_51_P204402	2.4	3.4.E-02	*Shcbp1*	NM_011369
A_51_P112355	2.4	2.3.E-02	*Igtp*	NM_018738
A_55_P1970110	2.4	3.2.E-02	*Gm281*	XM_138893
A_55_P1954086	2.4	4.7.E-02	*Postn*	NM_001198766

A_55_P2472435	2.4	4.0.E-02	*Gbp3*	NM_018734
A_55_P2003221	2.3	3.6.E-02	*Gimap3*	NM_031247
A_52_P180373	2.3	3.5.E-02	*Mpeg1*	NM_010821
A_52_P502771	2.3	3.7.E-02	*Rad54b*	NM_001039556
A_51_P234956	2.3	3.0.E-02	*Xcl1*	NM_008510
A_55_P2079991	2.3	3.8.E-02	*Gipc3*	NM_148951
A_51_P505493	2.3	3.7.E-02	*Elovl5*	NM_134255
A_55_P2137611	2.3	2.7.E-02	*Irgm2*	NM_019440
A_51_P484111	2.3	3.8.E-02	*Matn2*	NM_016762
A_55_P2158404	2.3	4.1.E-02	*Cmpk2*	NM_020557
A_55_P2077901	2.3	4.9.E-02	*Cd2*	NM_013486
A_55_P1982499	2.2	4.8.E-02	*Gldn*	NM_177350
A_55_P1975045	2.2	5.0.E-02	*Sgcg*	NM_011892
A_66_P118513	2.2	3.0.E-02	*Sept11*	NM_001009818
A_51_P181565	2.2	4.5.E-02	*Hbegf*	NM_010415
A_51_P335569	2.2	4.4.E-02	*Slco1a4*	NM_030687
A_55_P2030752	2.2	4.2.E-02	*Nedd4l*	NM_031881
A_55_P2022678	2.2	4.1.E-02	*C1qtnf1*	NM_001204129
A_55_P2293007	2.2	4.3.E-02	*AI662175*	AK139019
A_55_P1981455	2.2	4.2.E-02	*N/A*	N/A
A_55_P2072980	2.2	5.0.E-02	*Spna2*	NM_001177668
A_55_P2106106	2.2	4.1.E-02	*Gpr77*	NM_176912
A_55_P1964327	2.2	5.0.E-02	*Ppwd1*	NM_172807
A_51_P446825	2.2	4.5.E-02	*6430573F11Rik*	NM_176952
A_51_P439876	2.1	4.7.E-02	*Map4k4*	NM_008696
A_52_P1044655	2.1	4.3.E-02	*Vcan*	NM_172955
A_55_P1982201	2.1	3.4.E-02	*Sgsm2*	NM_197943
A_51_P215077	2.1	4.1.E-02	*Mgst3*	NM_025569
A_51_P246773	2.1	4.8.E-02	*Sesn3*	NM_030261
A_51_P321341	2.1	5.0.E-02	*Sult1a1*	NM_133670
A_55_P2007273	2.1	4.2.E-02	*Pole*	NM_011132
A_55_P2136678	2.1	3.5.E-02	*N/A*	XR_108249
A_55_P2109227	2.1	3.5.E-02	*Glt1d1*	NM_177005
A_51_P451346	2.1	3.3.E-02	*Klf6*	NM_011803
A_55_P2035286	2.1	3.3.E-02	*Uhrf1*	NM_010931
A_66_P101519	2.1	3.5.E-02	*Abcc9*	NM_021041
A_55_P2133266	2.0	4.1.E-02	*Cxx1c*	NM_028375
A_55_P2028259	2.0	4.6.E-02	*Rhbdf2*	NM_172572
A_55_P2273656	2.0	4.1.E-02	*N/A*	AK015069
A_51_P349192	2.0	4.9.E-02	*2210013O21Rik*	NR_028432
A_55_P1963454	2.0	3.4.E-02	*Cenpq*	NM_031863

A_55_P2109857	2.0	4.3.E-02	*Rgs2*	NM_009061
A_52_P493091	2.0	4.5.E-02	*Fmr1*	NM_008031
A_55_P2031357	2.0	4.3.E-02	*Zfpm2*	NM_011766
A_66_P106905	1.9	4.6.E-02	*N/A*	AK144962
A_52_P229943	1.9	4.5.E-02	*Ostb*	NM_178933
A_55_P2096883	1.9	4.0.E-02	*Steap3*	NM_001085409
A_51_P323531	1.9	4.6.E-02	*Fam71e1*	NM_028169
A_55_P2036007	1.9	4.3.E-02	*Rai2*	NM_198409
A_55_P2042655	1.9	4.9.E-02	*2210013O21Rik*	NR_028432
A_55_P1994939	1.9	4.4.E-02	*Hmgb2*	NM_008252
A_52_P1831	1.9	4.2.E-02	*Zfp60*	NM_009560
A_55_P1988246	1.9	4.1.E-02	*N/A*	N/A
A_51_P382152	1.9	4.3.E-02	*Procr*	NM_011171
A_55_P2097808	1.8	4.5.E-02	*P4ha1*	NM_011030
A_55_P2166501	1.8	4.5.E-02	*Cd44*	NM_009851

Downregulated entities

Agilent probe ID	Fold change	p-value (Corr)	Gene Symbol	Genbank Accession
A_55_P2032079	22.8	4.9.E-02	*Dbp*	NM_016974
A_51_P211351	16.0	2.1.E-02	*Olfr677*	NM_146358
A_51_P173976	14.6	2.1.E-02	*Olfr935*	NM_146746
A_55_P2156219	13.2	4.5.E-02	*Gm10549*	AK141051
A_55_P1989738	10.6	2.2.E-02	*4930426L09Rik*	NR_024323
A_55_P1966635	10.1	1.6.E-02	*Vmn2r75*	NM_001102578
A_55_P2055834	9.5	1.6.E-02	*N/A*	AK090145
A_55_P2135153	9.0	2.4.E-02	*Lcn13*	NM_153558
A_55_P2221236	8.1	2.1.E-02	*AU024180*	AK085976
A_55_P1985788	7.8	3.9.E-02	*Usp2*	NM_198092
A_66_P130650	7.4	2.1.E-02	*Gm9979*	XM_003085861
A_55_P1989149	7.3	3.1.E-02	*Olfr394*	NM_147007
A_55_P2053704	6.9	2.1.E-02	*Pglyrp4*	NM_001165968
A_55_P2032423	6.9	2.1.E-02	*N/A*	N/A
A_55_P2103033	6.9	2.3.E-02	*N/A*	N/A
A_52_P248013	6.5	2.1.E-02	*Chsy3*	NM_001081328
A_55_P2356263	6.4	2.3.E-02	*Anks1b*	AK036288
A_55_P2007646	6.4	2.2.E-02	*Cryaa*	NM_013501
A_55_P1971744	6.3	2.2.E-02	*4921511M17Rik*	NM_001201358
A_66_P129763	6.2	2.3.E-02	*Gm6329*	AK144087
A_55_P2142334	6.2	2.1.E-02	*Ly6h*	NM_011837
A_55_P2140731	6.1	3.9.E-02	*Gm10876*	XM_003085005

A_55_P2169410	6.1	2.1.E-02	*4930583I09Rik*	XM_128729
A_51_P208987	6.1	2.3.E-02	*Pgm3*	NM_028352
A_55_P2069760	6.0	2.1.E-02	*Sprr2b*	NM_011469
A_55_P2125557	6.0	2.1.E-02	*Irx2*	NM_010574
A_55_P2111267	5.9	2.1.E-02	*Lhx9*	NM_001042577
A_55_P1952935	5.9	2.3.E-02	*Olfr151*	NM_207664
A_55_P2057430	5.9	2.3.E-02	*Lipn*	NM_027340
A_55_P2110542	5.8	2.7.E-02	*N/A*	N/A
A_55_P2134725	5.8	2.1.E-02	*N/A*	N/A
A_55_P2116165	5.7	2.3.E-02	*Pfkfb3*	NM_001177753
A_55_P2073094	5.7	2.1.E-02	*Trex2*	NM_011907
A_52_P347942	5.7	2.1.E-02	*Olfr1384*	NM_146472
A_55_P2158663	5.7	2.1.E-02	*Cldn7*	N/A
A_51_P243418	5.6	2.1.E-02	*Clec2g*	NM_027562
A_55_P2075127	5.6	2.3.E-02	*Pax2*	NM_011037
A_55_P2122300	5.5	2.1.E-02	*Cir1*	NM_025854
A_55_P2018594	5.5	2.7.E-02	*N/A*	XM_888852
A_51_P102122	5.4	2.3.E-02	*Myog*	NM_031189
A_55_P2069679	5.4	3.5.E-02	*Olfr746*	NM_146298
A_55_P2207186	5.3	2.3.E-02	*Slc30a7*	NM_023214
A_66_P122730	5.1	2.3.E-02	*N/A*	AK138367
A_55_P1987117	5.1	2.3.E-02	*Slc22a16*	NM_027572
A_55_P2344593	5.1	2.1.E-02	*N/A*	AK016119
A_51_P218953	5.1	2.3.E-02	*Zfp536*	NM_172385
A_55_P1994733	5.1	2.3.E-02	*Tmem14a*	AK017734
A_51_P151246	5.0	2.1.E-02	*Olfr924*	NM_207560
A_55_P2143683	5.0	2.1.E-02	*Gm9927*	AK034060
A_55_P1975682	5.0	2.3.E-02	*Rhox2e*	NM_001085348
A_52_P329398	4.9	3.0.E-02	*Atp12a*	NM_138652
A_55_P1973447	4.9	3.1.E-02	*Ybx2*	N/A
A_51_P228159	4.9	2.1.E-02	*4930430O22Rik*	XM_003085348
A_55_P2091413	4.9	5.0.E-02	*N/A*	XM_978485
A_55_P2136788	4.9	2.1.E-02	*N/A*	N/A
A_52_P529660	4.8	2.3.E-02	*Lrrc52*	NM_001013382
A_66_P126877	4.7	2.3.E-02	*Vmn1r214*	NM_134214
A_55_P2160691	4.7	2.1.E-02	*N/A*	CK031974
A_55_P2036585	4.7	3.5.E-02	*Gm13547*	AK133213
A_55_P1979893	4.7	3.9.E-02	*Tef*	NM_017376
A_55_P2391185	4.7	2.1.E-02	*AI195381*	DV044254
A_55_P2119907	4.6	2.1.E-02	*Dnahc11*	NM_010060
A_55_P2027708	4.6	2.1.E-02	*5830416P10Rik*	NR_028427

A_55_P2106068	4.5	2.7.E-02	*Myh2*	NM_001039545
A_55_P2128195	4.5	3.3.E-02	*Pm20d2*	NM_001034867
A_55_P1958798	4.5	2.1.E-02	*Mobp*	NM_008614
A_55_P2062171	4.5	3.4.E-02	*Odf3*	AK005883
A_55_P2159717	4.5	2.1.E-02	*Gm5591*	NM_001013810
A_55_P1999187	4.5	3.1.E-02	*Cdk12*	NM_026952
A_55_P1954393	4.5	2.1.E-02	*Susd4*	NM_144796
A_55_P2122479	4.5	2.3.E-02	*N/A*	DV656125
A_52_P262930	4.5	3.1.E-02	*2310081J21Rik*	AK036683
A_55_P1956598	4.4	3.3.E-02	*N/A*	BC019489
A_51_P392429	4.4	2.1.E-02	*Mrgpre*	NM_175534
A_66_P122621	4.4	4.1.E-02	*Gm9889*	AK038322
A_66_P138178	4.4	2.1.E-02	*Gm4107*	AK041437
A_51_P514256	4.4	2.1.E-02	*Tubb2b*	NM_023716
A_55_P2058761	4.4	2.1.E-02	*G6pc2*	NM_021331
A_55_P1997345	4.4	2.1.E-02	*Gm11568*	NM_001205030
A_55_P2086730	4.3	2.3.E-02	*Olfr910*	NM_146811
A_55_P2083186	4.3	2.3.E-02	*LOC100009614*	NM_001081452
A_55_P2094108	4.3	2.1.E-02	*N/A*	N/A
A_51_P263965	4.3	2.1.E-02	*Hmox1*	NM_010442
A_55_P2083307	4.3	5.0.E-02	*Capn8*	NM_130890
A_55_P2145977	4.2	2.1.E-02	*Fbxw15*	NM_199036
A_55_P2023988	4.2	2.1.E-02	*Gm8008*	XM_001477516
A_55_P2083909	4.2	2.7.E-02	*N/A*	N/A
A_66_P130813	4.2	2.1.E-02	*Samd4*	NM_001037221
A_55_P1994147	4.1	2.3.E-02	*Zar1*	NM_174877
A_55_P2099947	4.1	3.0.E-02	*Gdf1*	NM_001163282
A_52_P536494	4.1	4.9.E-02	*Mycn*	NM_008709
A_52_P514352	4.1	2.1.E-02	*Kcnk5*	NM_021542
A_55_P2177371	4.0	2.1.E-02	*Gm3086*	NR_036607
A_55_P2173288	4.0	2.4.E-02	*Pcdhb1*	NM_053126
A_66_P101561	4.0	2.3.E-02	*Gm2176*	NR_028424
A_51_P409637	4.0	2.3.E-02	*4930505A04Rik*	NM_001100394
A_55_P2256393	3.8	2.2.E-02	*1700030L22Rik*	BU961976
A_55_P2369400	3.8	2.3.E-02	*1700066O22Rik*	NR_015541
A_66_P120732	3.8	2.3.E-02	*Gpr45*	NM_053107
A_55_P2187076	3.8	2.3.E-02	*Sncg*	NM_011430
A_55_P1981195	3.8	3.1.E-02	*N/A*	N/A
A_55_P2040071	3.8	4.0.E-02	*Eddm3b*	NM_203508
A_55_P2167171	3.8	2.1.E-02	*N/A*	N/A
A_55_P2068125	3.7	2.1.E-02	*Myct1*	NM_026793

A_66_P132787	3.7	3.1.E-02	*N/A*	M11859
A_55_P2217851	3.6	2.7.E-02	*4930453L07Rik*	AK015455
A_52_P11402	3.6	2.3.E-02	*Lrrc24*	NM_198119
A_55_P2058220	3.6	4.0.E-02	*Prlhr*	NM_201615
A_55_P2012439	3.6	3.8.E-02	*Tnfrsf19*	NM_013869
A_55_P2034033	3.6	2.1.E-02	*Il12rb1*	NM_008353
A_55_P2132800	3.5	2.3.E-02	*Zfp367*	AK041361
A_51_P121962	3.5	3.9.E-02	*Lphn3*	AK051766
A_55_P2156727	3.5	3.3.E-02	*Gm16509*	XM_001473385
A_55_P2378030	3.5	2.1.E-02	*4933439N14Rik*	AK019859
A_55_P2128113	3.5	2.2.E-02	*Tbc1d12*	NM_145952
A_55_P2118604	3.5	2.3.E-02	*St6galnac1*	NM_011371
A_55_P2012779	3.5	2.7.E-02	*Rnf167*	NM_027445
A_52_P217240	3.4	2.1.E-02	*Ppme1*	NM_028292
A_51_P184681	3.4	2.1.E-02	*Olfr675*	NM_001011848
A_55_P2097773	3.4	4.1.E-02	*Msh4*	NM_031870
A_52_P402127	3.4	3.2.E-02	*Mup9*	NM_001126319
A_55_P2039514	3.4	3.0.E-02	*N/A*	N/A
A_55_P2156780	3.4	2.1.E-02	*N/A*	CJ236021
A_51_P229664	3.4	2.1.E-02	*Cd27*	NM_001033126
A_55_P2230154	3.4	2.1.E-02	*A730046J19Rik*	NR_040271
A_55_P2033381	3.4	2.1.E-02	*1810041L15Rik*	NM_001163145
A_52_P275069	3.4	3.2.E-02	*Gm6792*	NM_001177416
A_55_P2069197	3.3	2.5.E-02	*Tas2r125*	NM_207027
A_55_P1973926	3.3	3.0.E-02	*Ipo13*	NM_146152
A_55_P2364755	3.3	3.7.E-02	*N/A*	XM_003086948
A_51_P377228	3.3	4.2.E-02	*N/A*	AK044378
A_52_P520341	3.3	2.1.E-02	*N/A*	AK088084
A_55_P2055112	3.3	3.2.E-02	*Prss44*	AB047758
A_55_P2289824	3.3	3.3.E-02	*1700121L16Rik*	AK007232
A_66_P103052	3.3	2.1.E-02	*N/A*	N/A
A_55_P2061104	3.2	3.2.E-02	*Mup6*	NM_001081285
A_51_P153423	3.2	3.1.E-02	*Fndc1*	NM_001081416
A_55_P2131168	3.2	2.3.E-02	*Sv2c*	NM_029210
A_55_P2089488	3.2	5.0.E-02	*Coq10b*	NM_001039710
A_55_P2019520	3.2	2.4.E-02	*Cma2*	NM_001024714
A_55_P2122896	3.2	2.1.E-02	*N/A*	N/A
A_55_P2000007	3.2	2.3.E-02	*N/A*	N/A
A_52_P502226	3.2	3.7.E-02	*2810429I04Rik*	NR_015522
A_51_P450505	3.2	3.3.E-02	*Olfr835*	NM_001012266
A_55_P2425801	3.2	3.2.E-02	*Fmn1*	NM_010230

A_55_P2079569	3.2	2.1.E-02	*Gm10555*	XM_003086208
A_66_P112495	3.2	2.3.E-02	*Scn4b*	NM_001013390
A_55_P2103756	3.2	2.1.E-02	*Hic2*	NM_178922
A_55_P2370384	3.2	3.0.E-02	*1600029I14Rik*	NR_028123
A_55_P1960659	3.2	2.3.E-02	*Fer1l6*	XM_905719
A_55_P2059732	3.1	2.5.E-02	*N/A*	N/A
A_51_P467668	3.1	4.3.E-02	*Ick*	NM_019987
A_51_P167843	3.1	2.3.E-02	*Timm17a*	NM_011590
A_55_P1966874	3.1	2.1.E-02	*Rtbdn*	NM_144929
A_55_P2008996	3.1	4.8.E-02	*Gm10034*	AK164250
A_52_P627269	3.1	2.3.E-02	*Ces2b*	NM_198171
A_55_P2000289	3.1	3.0.E-02	*Mab21l2*	NM_011839
A_51_P224593	3.1	2.1.E-02	*Arl8a*	NM_026823
A_52_P195246	3.1	2.7.E-02	*Esyt3*	NM_177775
A_55_P2169247	3.1	4.7.E-02	*Gm15056*	NM_001177471
A_55_P2007703	3.1	3.3.E-02	*Nanogpd*	NM_001080945
A_55_P2150328	3.1	3.2.E-02	*N/A*	N/A
A_55_P2067757	3.0	3.5.E-02	*Psg27*	NM_001037168
A_52_P318361	3.0	2.1.E-02	*Ces2c*	NM_145603
A_51_P417876	3.0	2.1.E-02	*Smyd5*	NM_144918
A_55_P2041082	3.0	2.3.E-02	*N/A*	N/A
A_55_P2013710	3.0	3.5.E-02	*Apoc3*	N/A
A_51_P226527	3.0	4.3.E-02	*Vmn1r85*	NM_145847
A_55_P2065671	3.0	3.2.E-02	*Ccnb1*	NM_172301
A_55_P2208463	3.0	2.3.E-02	*6820426E19Rik*	XR_105017
A_55_P2142488	3.0	3.2.E-02	*Spry3*	NM_001030293
A_51_P517145	3.0	3.2.E-02	*Sort1*	NM_019972
A_55_P2124712	2.9	2.3.E-02	*Ces2c*	NM_145603
A_55_P2234889	2.9	3.1.E-02	*C78704*	CA882486
A_55_P2007816	2.9	4.1.E-02	*Mup4*	NM_008648
A_55_P2186302	2.9	4.3.E-02	*Gm9866*	XR_035276
A_55_P2104587	2.9	3.3.E-02	*N/A*	N/A
A_51_P396163	2.9	4.4.E-02	*Mdm1*	M20824
A_55_P1997585	2.9	3.5.E-02	*N/A*	N/A
A_55_P2089840	2.9	2.3.E-02	*Eif2d*	NM_001136070
A_55_P2236291	2.8	2.3.E-02	*Ppap2a*	NM_008903
A_51_P288010	2.8	2.6.E-02	*Chrng*	NM_009604
A_55_P2005501	2.8	4.4.E-02	*Olfr392*	NM_147006
A_55_P2290378	2.8	3.3.E-02	*AU019176*	BM228091
A_55_P2109327	2.8	2.1.E-02	*Ache*	NM_009599
A_55_P2099363	2.8	4.6.E-02	*Stac2*	NM_146028

A_55_P2046671	2.8	3.0.E-02	*N/A*	N/A
A_55_P2092501	2.8	3.7.E-02	*Med1*	NM_134027
A_55_P1969131	2.8	4.3.E-02	*Cidec*	NM_178373
A_55_P2006930	2.8	3.4.E-02	*2610005L07Rik*	BC025151
A_55_P2275249	2.8	3.0.E-02	*Th*	NM_009377
A_55_P2041668	2.8	4.3.E-02	*Foxl2*	NM_012020
A_51_P516119	2.8	2.7.E-02	*Olfr1148*	NM_001011519
A_51_P377812	2.8	2.1.E-02	*1700011E24Rik*	NM_029298
A_51_P340668	2.8	3.2.E-02	*Bcl9l*	NM_030256
A_52_P233305	2.7	3.3.E-02	*Adamts12*	NM_175501
A_55_P2082454	2.7	3.7.E-02	*N/A*	N/A
A_55_P2152240	2.7	2.3.E-02	*Gm10383*	AK156988
A_55_P1959891	2.7	2.9.E-02	*N/A*	N/A
A_55_P2440441	2.7	3.2.E-02	*Wdhd1*	NM_172598
A_55_P2182392	2.7	2.3.E-02	*Adat3*	NM_001100606
A_55_P2335723	2.7	3.8.E-02	*2900009J06Rik*	AA986756
A_52_P676255	2.7	3.4.E-02	*Itpripl1*	NM_001163527
A_66_P114449	2.7	3.2.E-02	*Vps39*	NM_147153
A_55_P2127243	2.7	3.2.E-02	*Tpsb2*	NM_010781
A_51_P500661	2.7	3.3.E-02	*Rnf182*	NM_183204
A_55_P2039289	2.7	3.2.E-02	*Hspb6*	NM_001012401
A_55_P2038882	2.7	2.4.E-02	*Niacr1*	NM_030701
A_55_P2205760	2.7	2.3.E-02	*C88045*	C86380
A_55_P2370160	2.7	4.9.E-02	*C130009A20Rik*	AK142864
A_55_P2180086	2.7	3.9.E-02	*Lrrc28*	NM_175124
A_55_P2116794	2.7	3.3.E-02	*N/A*	N/A
A_51_P228971	2.7	3.2.E-02	*Slc5a4b*	NM_023219
A_55_P2146789	2.7	3.7.E-02	*N/A*	N/A
A_55_P2037930	2.6	4.1.E-02	*N/A*	N/A
A_52_P272145	2.6	4.5.E-02	*N/A*	N/A
A_55_P2250164	2.6	2.1.E-02	*Pcdhgc5*	NM_033583
A_55_P2130438	2.6	3.3.E-02	*6530409C15Rik*	XM_987873
A_51_P316199	2.6	3.3.E-02	*Olfr73*	NM_054090
A_55_P2166069	2.6	3.7.E-02	*2310079G19Rik*	NM_027173
A_52_P395242	2.6	2.3.E-02	*Map2k6*	AK086722
A_66_P112593	2.6	4.0.E-02	*Sec61b*	NM_024171
A_55_P2032868	2.6	2.2.E-02	*4932412H11Rik*	NM_172879
A_55_P2023391	2.6	2.3.E-02	*Grhl3*	NM_001013756
A_52_P204618	2.6	2.3.E-02	*Rnf222*	NM_177060
A_55_P2083257	2.6	4.9.E-02	*A3galt2*	NM_001009819
A_55_P2100281	2.6	4.1.E-02	*Defb30*	DQ141309

A_55_P2024634	2.6	3.2.E-02	*N/A*	XR_104688
A_55_P2074015	2.6	4.1.E-02	*N/A*	N/A
A_52_P388164	2.6	2.3.E-02	*Olfr608*	NM_146756
A_55_P2124361	2.6	3.4.E-02	*N/A*	N/A
A_66_P134539	2.6	2.3.E-02	*A430110L20Rik*	XM_003084469
A_55_P2066862	2.6	3.2.E-02	*Nsun7*	NM_027602
A_55_P2009439	2.6	3.1.E-02	*N/A*	N/A
A_55_P2138422	2.5	3.4.E-02	*Obox1*	NM_027802
A_55_P2169445	2.5	2.3.E-02	*Mfsd6*	NM_133829
A_52_P682996	2.5	3.5.E-02	*N/A*	N/A
A_55_P2022778	2.5	4.9.E-02	*Trpc2*	NM_011644
A_51_P271068	2.5	2.3.E-02	*Klf17*	NM_029416
A_55_P1987953	2.5	3.1.E-02	*Gm5938*	NM_001085534
A_55_P2070938	2.5	4.8.E-02	*N/A*	N/A
A_55_P2157537	2.5	3.8.E-02	*Gm10866*	XM_003085086
A_55_P2044803	2.5	3.2.E-02	*N/A*	AK085886
A_55_P2034091	2.5	4.6.E-02	*N/A*	N/A
A_55_P2008141	2.5	2.7.E-02	*Gm10857*	NR_033470
A_52_P1092823	2.5	2.3.E-02	*Irx1*	NM_010573
A_55_P1958532	2.5	3.0.E-02	*Hr*	NM_021877
A_55_P1955871	2.5	2.2.E-02	*Nlrp4f*	NM_175290
A_52_P15377	2.5	3.6.E-02	*Wnt9b*	NM_011719
A_55_P2037737	2.5	3.4.E-02	*N/A*	N/A
A_55_P2045859	2.5	4.3.E-02	*N/A*	N/A
A_52_P150988	2.5	2.3.E-02	*Txndc11*	NM_029582
A_55_P1967578	2.5	4.1.E-02	*Olfr893*	NM_146336
A_52_P628212	2.5	2.3.E-02	*Esrrb*	AK044339
A_51_P330144	2.4	5.0.E-02	*Ift140*	NM_134126
A_55_P2009684	2.4	2.7.E-02	*Zmiz1*	AK169397
A_51_P398069	2.4	5.0.E-02	*Olfr992*	NM_146865
A_51_P219483	2.4	3.2.E-02	*Tsku*	NM_001024619
A_51_P250571	2.4	3.6.E-02	*Tas2r140*	NM_021562
A_55_P2157952	2.4	3.1.E-02	*Gm10755*	XM_003086133
A_55_P1965821	2.4	3.1.E-02	*Uprt*	NM_001081189
A_51_P313503	2.4	3.2.E-02	*Olfr577*	NM_147109
A_55_P2116674	2.4	4.1.E-02	*N/A*	N/A
A_55_P2127194	2.4	3.1.E-02	*N/A*	N/A
A_66_P135403	2.4	2.3.E-02	*AI481877*	AK135748
A_55_P2074045	2.4	4.9.E-02	*N/A*	N/A
A_55_P2181216	2.4	3.2.E-02	*N/A*	N/A
A_55_P2082519	2.4	3.5.E-02	*Olfr883*	NM_146419

A_51_P201308	2.4	3.4.E-02	*Moxd2*	NM_139296
A_55_P1972001	2.4	3.3.E-02	*LOC100039183*	XM_003086064
A_55_P1963006	2.4	4.9.E-02	*N/A*	N/A
A_55_P2182112	2.4	3.2.E-02	*4833439L19Rik*	NM_133797
A_66_P104751	2.4	4.1.E-02	*2210404J11Rik*	AK019108
A_51_P262489	2.4	3.0.E-02	*Sst*	NM_009215
A_55_P2085518	2.3	3.9.E-02	*N/A*	N/A
A_55_P1974917	2.3	2.3.E-02	*Olfr368*	NM_146374
A_55_P1974522	2.3	3.2.E-02	*A530099J19Rik*	NM_175688
A_52_P344290	2.3	4.3.E-02	*F2r*	NM_010169
A_51_P408199	2.3	4.1.E-02	*Krtap4-2*	NM_026807
A_55_P2026385	2.3	3.7.E-02	*N/A*	N/A
A_66_P137048	2.3	4.1.E-02	*Men1*	NM_008583
A_51_P521052	2.3	3.8.E-02	*Ly6k*	NM_029627
A_55_P2107647	2.3	4.2.E-02	*N/A*	N/A
A_66_P117534	2.3	2.5.E-02	*E030025P04Rik*	NR_037978
A_55_P2054708	2.3	3.2.E-02	*N/A*	AK143362
A_51_P259318	2.3	2.7.E-02	*Fbrs*	NM_010183
A_52_P647607	2.3	5.0.E-02	*Ubr4*	NM_001160319
A_55_P1958227	2.2	3.7.E-02	*N/A*	AK135304
A_55_P2114110	2.2	5.0.E-02	*Cadm4*	NM_153112
A_55_P1993148	2.2	4.7.E-02	*Ascl4*	NM_001163614
A_52_P523368	2.2	3.2.E-02	*Psapl1*	NM_175249
A_52_P1045316	2.2	2.7.E-02	*Gm428*	NM_001081644
A_55_P1961400	2.2	3.2.E-02	*Grid2ip*	NM_133355
A_55_P2091274	2.2	3.5.E-02	*Sly*	NM_201530
A_55_P2151675	2.2	3.9.E-02	*N/A*	AK164979
A_51_P186956	2.2	3.5.E-02	*Adam2*	NM_009618
A_55_P1996344	2.2	4.1.E-02	*N/A*	N/A
A_55_P2283207	2.2	3.0.E-02	*Maz*	NM_010772
A_51_P429366	2.2	3.6.E-02	*Hes6*	NM_019479
A_55_P2154450	2.2	3.7.E-02	*Xkr6*	NM_173393
A_55_P2019428	2.2	3.8.E-02	*Htr1d*	NM_008309
A_51_P496309	2.2	4.1.E-02	*Rfx4*	NM_001024918
A_52_P179785	2.2	3.2.E-02	*Ripk2*	NM_138952
A_55_P1952882	2.2	3.2.E-02	*Cyp4f18*	AF233647
A_66_P107482	2.2	3.9.E-02	*Arhgef33*	NM_001145452
A_55_P2182586	2.2	4.5.E-02	*Esrp1*	NM_194055
A_51_P146149	2.2	4.1.E-02	*Napsa*	NM_008437
A_52_P81693	2.2	3.8.E-02	*LOC100502592*	XM_003084652
A_52_P496726	2.2	5.0.E-02	*Rasd1*	NM_009026

A_55_P2061899	2.2	4.1.E-02	*N/A*	N/A
A_55_P2077263	2.2	4.1.E-02	*Cenpk*	NM_021790
A_55_P1978895	2.2	3.2.E-02	*Skint3*	NM_177578
A_55_P1964237	2.1	3.7.E-02	*4932418E24Rik*	NM_177841
A_55_P2141058	2.1	3.9.E-02	*LOC100504710*	NM_001201389
A_55_P1963767	2.1	3.6.E-02	*Npr2*	NM_173788
A_65_P18756	2.1	3.9.E-02	*Klf17*	NM_029416
A_55_P2101939	2.1	3.3.E-02	*Zfp112*	NM_021307
A_66_P115959	2.1	3.8.E-02	*1700016H13Rik*	NM_001163550
A_55_P2044054	2.1	3.4.E-02	*AA465934*	BB850827
A_55_P1954511	2.1	4.3.E-02	*Btbd10*	NM_133700
A_51_P356842	2.1	4.6.E-02	*Olfr320*	NM_207230
A_55_P2016976	2.1	3.3.E-02	*A530054K11Rik*	NM_183146
A_55_P2182931	2.1	4.1.E-02	*Sim2*	NM_011377
A_55_P2153923	2.1	3.9.E-02	*Slc17a5*	NM_172773
A_55_P1959985	2.1	4.5.E-02	*Alas1*	NM_020559
A_55_P2017343	2.1	3.3.E-02	*Olfr767*	NM_146318
A_55_P2039530	2.1	4.1.E-02	*Gm10724*	XM_003086271
A_55_P2067747	2.1	3.8.E-02	*Psg21*	NM_027403
A_55_P1991069	2.1	4.3.E-02	*Gm885*	NM_001033435
A_51_P443514	2.1	4.4.E-02	*1700001K19Rik*	NM_025488
A_51_P448784	2.1	4.8.E-02	*Nrsn1*	NM_009513
A_55_P2246665	2.1	3.8.E-02	*4833422M21Rik*	AK014752
A_55_P2002642	2.1	3.8.E-02	*Ccdc67*	NM_181816
A_52_P660422	2.1	4.5.E-02	*Mip*	NM_008600
A_52_P362917	2.0	4.8.E-02	*Pfkfb3*	NM_133232
A_51_P342307	2.0	4.6.E-02	*Olfr732*	NM_146665
A_51_P358316	2.0	4.9.E-02	*Chga*	NM_007693
A_55_P2136817	2.0	4.5.E-02	*Coro2b*	NM_175484
A_55_P2398995	2.0	4.2.E-02	*Lelp1*	NM_027042
A_52_P203770	2.0	4.5.E-02	*Vmn2r61*	NM_001105058
A_55_P2139878	2.0	4.3.E-02	*N/A*	N/A
A_51_P102421	2.0	3.5.E-02	*Clcf1*	NM_019952
A_55_P2020911	2.0	4.0.E-02	*Rps6ka6*	NM_025949
A_52_P466853	2.0	3.7.E-02	*Bmyc*	NM_023326
A_55_P2078257	2.0	4.7.E-02	*Gm10183*	AK172566
A_55_P2168736	2.0	4.3.E-02	*Relb*	NM_009046
A_55_P2312741	2.0	4.8.E-02	*AU046084*	BG072043
A_55_P2013470	1.9	5.0.E-02	*Gm14743*	NM_001126321
A_51_P264444	1.9	4.0.E-02	*Ralgapa2*	AK038838
A_55_P2290388	1.9	4.9.E-02	*Rorb*	NM_001043354

A_51_P171616	1.9	4.1.E-02	*Wnt10a*	NM_009518
A_66_P133213	1.9	4.1.E-02	*Slc35f1*	NM_178675
A_55_P1967391	1.9	4.8.E-02	*Antxr1*	AK013005
A_51_P161554	1.9	4.7.E-02	*Arid3b*	NM_019689

Supplementary table II. Aging signature in Bach1 knockout mice liver

Upregulated entities

Agilent probe ID	Fold change	p-value (Corr)	Gene Symbol	Genbank Accession
A_55_P1952517	27076.3	2.1.E-06	*Sult2a1*	NM_001111296
A_55_P1952512	21192.4	2.1.E-06	*Sult2a2*	NM_009286
A_55_P1952507	9372.0	2.1.E-06	*Sult2a4*	NM_001101534
A_55_P2172532	7475.3	2.1.E-06	*Sult2a6*	NM_001081325
A_55_P2107223	7304.4	2.1.E-06	*Sult2a6*	NM_001081325
A_52_P73552	2459.9	2.0.E-05	*A1bg*	NM_001081067
A_52_P218833	1143.4	2.0.E-05	*Sult2a3*	NM_001101586
A_52_P366803	535.4	8.3.E-05	*Cyp3a44*	NM_177380
A_55_P1989248	527.4	2.1.E-06	*N/A*	N/A
A_51_P269404	340.1	2.5.E-03	*Fmo3*	NM_008030
A_55_P1991841	281.8	1.1.E-04	*Slc22a27*	NM_134256
A_55_P2020428	240.3	1.9.E-05	*Slc22a26*	NM_146232
A_51_P114722	150.7	8.3.E-05	*Hao2*	NM_019545
A_55_P2129449	117.6	1.5.E-02	*Sult3a1*	NM_020565
A_51_P144531	105.1	2.3.E-05	*Slc22a29*	NM_172776
A_55_P2107225	70.3	8.3.E-05	*Sult2a6*	NM_001081325
A_55_P2044653	61.0	1.4.E-05	*Cyp2b10*	NM_009999
A_52_P293682	56.8	2.2.E-03	*Sult2a7*	NM_001184981
A_52_P289091	55.6	4.3.E-04	*Cyp2b13*	NM_007813
A_55_P2034531	51.3	5.6.E-05	*N/A*	N/A
A_52_P256914	45.2	1.9.E-05	*Cyp2b9*	NM_010000
A_52_P493477	42.0	1.2.E-03	*Serpinb1c*	NM_173051
A_55_P2060138	33.5	1.1.E-03	*Cyp2a22*	NM_001101467
A_55_P2165091	32.7	1.5.E-03	*Acnat2*	NM_145368
A_55_P2075263	32.6	1.5.E-03	*Acnat2*	NM_145368
A_55_P1962516	32.4	2.5.E-04	*Fam19a2*	NM_182807
A_55_P2408588	29.7	8.4.E-04	*Arntl*	NM_007489
A_51_P269792	28.2	4.9.E-03	*Rad51l1*	NM_009014
A_55_P2098175	25.9	4.3.E-04	*Olfr701*	NM_028910
A_51_P362066	25.1	2.1.E-03	*Chi3l1*	NM_007695
A_51_P461067	24.3	1.3.E-03	*N/A*	X87228
A_55_P1955861	23.3	1.5.E-03	*Cux2*	NM_007804
A_55_P2071906	23.2	6.1.E-05	*N/A*	N/A
A_51_P386648	22.7	5.5.E-03	*Glod5*	NM_027227
A_55_P2030524	21.6	1.7.E-03	*Vldlr*	NM_013703
A_66_P107818	21.2	6.6.E-04	*Gdf3*	NM_008108
A_55_P2006008	20.7	2.1.E-03	*Serpinb1a*	NM_025429

A_52_P574668	19.9	1.0.E-03	*Nt5e*	NM_011851
A_51_P485421	18.8	1.8.E-03	*N/A*	AK088666
A_55_P2060897	18.7	1.9.E-04	*N/A*	AM910933
A_55_P2038347	18.5	8.1.E-03	*Acot3*	NM_134246
A_55_P2059931	18.0	3.4.E-04	*Prom1*	NM_001163577
A_55_P2069907	15.3	1.9.E-02	*Acot3*	NM_134246
A_51_P480119	15.3	5.2.E-04	*Prelid2*	NM_029942
A_52_P113537	14.4	4.2.E-03	*Xist*	NR_001463
A_51_P469951	14.3	7.9.E-04	*Srgap3*	NM_080448
A_55_P1999162	14.2	4.2.E-03	*N/A*	XR_030524
A_55_P2008437	14.1	3.8.E-03	*Cdkn2a*	NM_009877
A_52_P122649	14.0	2.5.E-03	*Dmrta1*	NM_175647
A_52_P235347	13.0	1.5.E-02	*Fgf21*	NM_020013
A_55_P1978666	13.0	4.2.E-04	*Sybu*	NM_176998
A_55_P1980214	12.9	4.6.E-03	*N/A*	N/A
A_51_P212782	12.1	1.9.E-02	*Il1b*	NM_008361
A_55_P2151609	11.9	2.3.E-03	*Sorl1*	NM_011436
A_55_P2001780	11.6	1.5.E-03	*Cyp3a41b*	NM_001105159
A_55_P1998471	11.3	5.4.E-03	*S100a9*	NM_009114
A_51_P271984	11.2	4.2.E-03	*Tmem45b*	NM_144936
A_51_P256827	10.7	5.9.E-03	*S100a8*	NM_013650
A_55_P2058028	10.6	7.7.E-03	*N/A*	X79554
A_65_P07626	10.4	1.9.E-03	*N/A*	U30234
A_51_P150710	10.1	1.2.E-03	*Igj*	NM_152839
A_55_P2126850	10.0	3.8.E-03	*Nudt11*	NM_021431
A_51_P440047	9.7	5.1.E-04	*1110067D22Ril*	NM_173752
A_52_P483336	9.6	2.4.E-03	*Ms4a1*	NM_007641
A_52_P21486	9.6	1.2.E-02	*Hamp2*	NM_183257
A_52_P230167	9.3	3.2.E-03	*Setbp1*	NM_053099
A_66_P122699	9.1	1.3.E-03	*Cux2*	AK139469
A_52_P301602	9.0	4.8.E-02	*Gm4794*	NM_001101452
A_51_P350453	8.8	6.2.E-03	*Pdk4*	NM_013743
A_52_P71686	8.7	6.5.E-03	*Atp6v0d2*	NM_175406
A_55_P2145059	8.4	3.5.E-03	*Gbp5*	AF487898
A_51_P248666	8.4	1.3.E-02	*Cd274*	NM_021893
A_51_P249302	8.4	5.2.E-04	*Abcd2*	NM_011994
A_55_P1983448	8.2	2.9.E-03	*S100a4*	NM_011311
A_55_P1973417	8.2	2.4.E-03	*N/A*	BC048373
A_52_P457567	8.1	4.7.E-03	*Slc4a4*	NM_018760
A_55_P2019601	8.1	2.1.E-03	*Csprs*	NM_033616
A_55_P2156062	8.1	7.9.E-04	*Pick1*	AK090155

A_55_P1987406	8.0	5.1.E-03	*N/A*	AY679096
A_51_P467448	7.9	6.1.E-03	*Pif1*	NM_172453
A_51_P204080	7.9	4.2.E-03	*Hk2*	NM_013820
A_52_P425839	7.9	4.6.E-03	*Retnlg*	NM_181596
A_51_P477121	7.9	3.8.E-03	*Pmaip1*	NM_021451
A_55_P1963134	7.7	6.5.E-03	*N/A*	N/A
A_66_P124179	7.7	5.5.E-03	*Atp6v0d2*	NM_175406
A_51_P281333	7.6	8.4.E-04	*St3gal6*	NM_018784
A_55_P1961736	7.4	2.1.E-03	*Rcan2*	NM_207649
A_51_P311958	7.3	5.4.E-04	*Orm3*	NM_013623
A_55_P2179027	7.3	1.0.E-02	*Gem*	NM_010276
A_52_P468564	7.2	1.2.E-03	*Cyp2c38*	NM_010002
A_51_P488196	7.2	4.6.E-03	*Bmper*	NM_028472
A_55_P2080210	7.2	2.5.E-03	*Crisp4*	NM_030033
A_55_P1982186	7.1	5.7.E-03	*Sgsm1*	NM_172718
A_55_P2091191	6.8	5.3.E-03	*Slc28a2*	NM_172980
A_66_P109397	6.8	7.4.E-03	*Cdh19*	NM_001081386
A_55_P2058040	6.8	8.9.E-03	*N/A*	X06519
A_51_P509573	6.7	2.4.E-03	*Ccl4*	NM_013652
A_55_P2137688	6.7	4.2.E-03	*Cga*	NM_009889
A_52_P449871	6.6	2.5.E-03	*Id4*	NM_031166
A_55_P2044143	6.5	3.0.E-03	*Loxl4*	NM_001164311
A_55_P1971076	6.4	4.1.E-03	*Atp11a*	NM_015804
A_51_P414879	6.4	7.1.E-03	*N/A*	S60874
A_55_P2174836	6.4	3.1.E-03	*Vldlr*	NM_013703
A_55_P2033041	6.4	3.5.E-03	*Sirpb1b*	NM_001173460
A_51_P375146	6.4	1.5.E-03	*Cd36*	NM_007643
A_51_P238576	6.4	4.5.E-03	*Cyp4a14*	NM_007822
A_52_P402786	6.3	2.5.E-03	*Prom1*	NM_008935
A_51_P247637	6.2	1.6.E-03	*Rnf144a*	NM_080563
A_52_P16232	6.1	7.8.E-03	*Gabbr1*	NM_019439
A_55_P2064771	6.0	4.0.E-03	*Ly6c1*	NM_010741
A_55_P2083609	6.0	4.1.E-03	*Dkk3*	NM_015814
A_55_P2103011	5.9	2.6.E-03	*Sema4d*	NM_013660
A_55_P2055158	5.9	4.7.E-03	*Apod*	NM_007470
A_51_P304683	5.9	4.2.E-03	*Clpx*	NM_011802
A_52_P244803	5.9	1.1.E-02	*D630033O11Ri*	AK052711
A_55_P2296088	5.9	1.1.E-02	*Sp3*	NM_001018042
A_55_P2063508	5.9	4.2.E-03	*Gm4131*	BY732610
A_55_P1978033	5.8	2.5.E-03	*N/A*	HQ015080
A_66_P114381	5.8	3.8.E-03	*Ypel2*	NM_001005341

A_55_P1995512	5.8	1.1.E-02	*Tmem139*	NM_175408
A_55_P2130535	5.8	3.5.E-03	*Dnmt3b*	NM_001003961
A_52_P413947	5.8	1.4.E-03	*Mthfr*	NM_010840
A_55_P2070869	5.7	2.1.E-03	*Lcn2*	NM_008491
A_51_P386899	5.6	7.3.E-03	*Mfsd7c*	NM_145447
A_52_P398925	5.6	4.7.E-03	*Stfa2l1*	NM_173869
A_55_P2082929	5.6	8.9.E-03	*H2-Ob*	NM_010389
A_55_P2001274	5.5	4.0.E-03	*N/A*	AY114394
A_51_P509679	5.5	7.3.E-03	*N/A*	XM_003085465
A_55_P1995537	5.4	3.9.E-02	*Mpo*	NM_010824
A_55_P1993807	5.4	4.9.E-03	*Nudt10*	NM_001031664
A_66_P139387	5.4	2.6.E-03	*Prlr*	NM_011169
A_55_P2066505	5.4	1.8.E-02	*Mpo*	AY500847
A_55_P1965836	5.4	6.7.E-03	*Crebbp*	NM_001025432
A_55_P1977850	5.4	1.2.E-03	*Sall1*	NM_021390
A_52_P329367	5.3	4.6.E-03	*Chka*	NM_001025566
A_55_P2112360	5.2	7.0.E-03	*Arhgap17*	NM_144529
A_55_P2041614	5.2	7.9.E-03	*Cerkl*	NM_001048176
A_66_P130887	5.2	1.2.E-02	*Pcdh18*	NM_130448
A_55_P2161055	5.2	6.6.E-03	*Trp63*	NM_001127261
A_55_P2186220	5.1	2.1.E-03	*N/A*	XM_001472058
A_51_P142923	5.1	9.2.E-03	*Chka*	NM_013490
A_55_P2125786	5.0	1.2.E-02	*Lrrc8e*	NM_028175
A_51_P286737	5.0	5.7.E-03	*Ccl2*	NM_011333
A_55_P2186978	5.0	1.5.E-02	*N/A*	U19317
A_55_P2145224	4.9	3.8.E-03	*N/A*	AF290571
A_55_P2049771	4.9	2.3.E-03	*N/A*	N/A
A_55_P2183369	4.8	7.4.E-03	*N/A*	U18577
A_55_P2058023	4.8	1.4.E-02	*N/A*	U18569
A_55_P1962224	4.8	8.6.E-03	*Afap1l2*	NM_146102
A_51_P378789	4.8	6.4.E-03	*Cxcl13*	NM_018866
A_55_P1988994	4.7	5.5.E-03	*N/A*	AY816714
A_51_P345649	4.7	2.5.E-03	*Pdgfra*	NM_011058
A_52_P420500	4.7	1.1.E-02	*Cry1*	NM_007771
A_51_P231320	4.7	4.4.E-02	*Mmp8*	NM_008611
A_51_P267354	4.7	2.5.E-03	*Lrfn3*	NM_175478
A_55_P2058550	4.7	4.7.E-03	*Raet1c*	NM_009018
A_55_P2006499	4.7	8.9.E-03	*Esrrg*	NM_011935
A_51_P199135	4.7	2.9.E-02	*Cd83*	NM_009856
A_66_P122086	4.7	5.6.E-03	*9030619P08Ril*	NM_001039720
A_55_P2077901	4.7	2.0.E-03	*Cd2*	NM_013486

A_51_P440743	4.6	1.0.E-02	*Celsr1*	NM_009886
A_55_P2010271	4.6	7.9.E-03	*Samsn1*	NM_023380
A_55_P1983152	4.6	3.9.E-03	*Gramd4*	NM_001205353
A_66_P101261	4.6	6.3.E-03	*Gm3367*	BX517038
A_51_P507801	4.6	1.0.E-02	*F13a1*	NM_028784
A_55_P2178510	4.6	9.3.E-03	*Mc3r*	NM_008561
A_52_P237077	4.6	8.7.E-03	*Esr1*	NM_007956
A_51_P423709	4.6	1.5.E-02	*Fam84a*	NM_029007
A_55_P1956418	4.5	1.2.E-02	*Efr3b*	NM_001082483
A_55_P1971074	4.5	3.3.E-03	*Atp11a*	NM_015804
A_55_P1964282	4.5	4.2.E-03	*Gm1078*	NM_001200041
A_65_P03874	4.5	9.1.E-03	*Nav2*	NM_175272
A_52_P93910	4.4	4.7.E-03	*Nrp2*	NM_001077403
A_55_P1982902	4.4	4.7.E-03	*Tceal3*	NM_001029978
A_55_P2072005	4.4	4.7.E-03	*Ctsc*	AK152524
A_52_P60353	4.4	4.6.E-03	*Greb1l*	NM_001083628
A_55_P1968433	4.4	4.2.E-03	*Agpat9*	NM_172715
A_55_P2043627	4.3	7.0.E-03	*Fam89a*	NM_001081120
A_52_P15388	4.3	7.9.E-03	*Ltf*	NM_008522
A_52_P413584	4.3	5.0.E-03	*Nrip1*	AK078829
A_55_P2074521	4.3	2.5.E-02	*Psg28*	NM_054063
A_51_P229498	4.3	1.5.E-02	*Lrfn2*	NM_027452
A_55_P2127387	4.3	2.4.E-02	*Olfr1023*	NM_146587
A_51_P302358	4.3	5.5.E-03	*Ltb*	NM_008518
A_51_P169516	4.2	9.2.E-03	*Ppp1r3d*	NM_001085501
A_52_P140005	4.2	8.7.E-03	*Nipal1*	NM_001081205
A_55_P2168048	4.2	1.6.E-02	*Gramd4*	NM_001205353
A_55_P1991079	4.1	4.7.E-03	*Phf21b*	NM_001081166
A_51_P257885	4.1	4.0.E-03	*Mmd2*	NM_175217
A_51_P483576	4.1	3.0.E-03	*N/A*	AF138742
A_55_P2048779	4.1	9.5.E-03	*Fam84b*	NM_001162926
A_51_P315042	4.1	8.9.E-03	*Avpr1a*	NM_016847
A_55_P2107207	4.1	7.0.E-03	*Gpr137b*	AK009736
A_55_P2058433	4.1	6.4.E-03	*Cyp2c68*	NM_001039555
A_52_P321140	4.1	4.2.E-02	*Defb1*	NM_007843
A_55_P1960148	4.0	1.2.E-02	*N/A*	XM_001472091
A_51_P172054	4.0	1.5.E-03	*Gas6*	NM_019521
A_55_P1976743	4.0	1.9.E-02	*N/A*	CJ206873
A_52_P213483	4.0	7.2.E-03	*N/A*	AF045501
A_65_P19089	4.0	6.3.E-03	*Esrrg*	NM_011935
A_55_P1985070	4.0	5.5.E-03	*Robo1*	NM_019413

A_55_P1969650	4.0	5.0.E-03	*Rasgrp1*	NM_011246
A_55_P2102936	3.9	1.3.E-02	*Pnrc1*	NM_001033225
A_55_P2090874	3.9	1.3.E-02	*Ysk4*	NM_011737
A_51_P290074	3.9	1.3.E-02	*Fabp7*	NM_021272
A_55_P2047589	3.9	8.1.E-03	*N/A*	X75105
A_55_P2002963	3.9	2.4.E-03	*Coro1a*	NM_009898
A_51_P439876	3.9	4.6.E-03	*Map4k4*	NM_008696
A_55_P1965045	3.9	1.5.E-02	*Slc47a2*	NM_001033542
A_55_P2079390	3.9	9.3.E-03	*4930579G24Ril*	NM_029482
A_51_P343517	3.9	5.1.E-03	*Ly6d*	NM_010742
A_52_P54280	3.9	1.3.E-02	*Adck3*	NM_023341
A_52_P294510	3.9	7.3.E-03	*Fgl1*	BC029734
A_66_P106148	3.8	8.6.E-03	*Raet1c*	NM_009018
A_55_P1967286	3.8	7.7.E-03	*N/A*	XM_357633
A_55_P2131060	3.8	5.4.E-03	*9530053H05Ril*	AK020608
A_65_P15245	3.8	1.0.E-02	*Nrp2*	NM_001077406
A_51_P136294	3.8	1.7.E-02	*Ms4a4b*	NM_021718
A_52_P195839	3.8	6.3.E-03	*Ctsc*	NM_009982
A_55_P2015670	3.8	5.5.E-03	*Itga6*	NM_008397
A_51_P229602	3.8	8.7.E-03	*Plxna2*	NM_008882
A_65_P08239	3.8	1.9.E-02	*Mef2a*	NM_001033713
A_52_P458000	3.8	4.7.E-03	*AI467606*	NM_178901
A_51_P286748	3.8	1.1.E-02	*Frzb*	NM_011356
A_52_P638459	3.7	8.3.E-03	*Ccl5*	NM_013653
A_55_P1982291	3.7	1.8.E-02	*Clca1*	NM_009899
A_55_P2017914	3.7	6.1.E-03	*Csf1*	NM_001113530
A_51_P498631	3.7	9.5.E-03	*Dfna5*	NM_018769
A_51_P303424	3.7	1.0.E-02	*Itgax*	NM_021334
A_51_P424810	3.7	1.1.E-02	*Ncapg2*	NM_133762
A_52_P357611	3.7	5.8.E-03	*Neu3*	NM_016720
A_52_P335064	3.7	9.2.E-03	*Mustn1*	NM_181390
A_55_P2017362	3.7	6.7.E-03	*BC068157*	NM_207203
A_55_P1955147	3.7	4.3.E-03	*Camk1d*	NM_177343
A_55_P2059110	3.7	9.3.E-03	*Skap1*	NM_001033186
A_55_P2102350	3.6	3.6.E-03	*Stambpl1*	NM_029682
A_51_P151732	3.6	1.1.E-02	*Pkp1*	NM_019645
A_55_P1993001	3.6	1.1.E-02	*N/A*	AF303868
A_55_P2035320	3.6	7.6.E-03	*Nfil3*	NM_017373
A_55_P2095039	3.6	2.0.E-02	*A330049M08Ri*	NM_001168500
A_52_P57622	3.6	7.5.E-03	*Acss3*	NM_198636
A_55_P2111322	3.6	7.3.E-03	*LOC10050535ℓ*	XR_105809

A_66_P100268	3.6	2.7.E-02	*N/A*	AK087888
A_51_P453111	3.6	4.6.E-03	*Hexb*	NM_010422
A_52_P525183	3.6	7.9.E-03	*Acot2*	NM_134188
A_55_P1996983	3.6	9.5.E-03	*4930430D24Ril*	NM_001034856
A_51_P314397	3.6	4.0.E-03	*Crip2*	NM_024223
A_55_P1988148	3.6	5.5.E-03	*Tox*	AK162821
A_55_P2045741	3.6	5.4.E-03	*Fyb*	NM_011815
A_66_P128918	3.6	1.7.E-02	*Hacl1*	NM_019975
A_66_P119034	3.5	3.5.E-03	*Pla2g7*	NM_013737
A_55_P2056542	3.5	2.1.E-02	*Nin*	NM_008697
A_51_P111612	3.5	1.4.E-02	*Arrdc4*	NM_001042592
A_52_P340073	3.5	8.6.E-03	*Efnb2*	NM_010111
A_52_P447439	3.5	6.1.E-03	*Prss43*	NM_199471
A_66_P121446	3.5	3.0.E-02	*Gm7969*	XM_982175
A_55_P2340448	3.5	4.1.E-03	*B230114P17Ri*	AK045415
A_55_P2181271	3.5	7.1.E-03	*Nhsl2*	NM_001163610
A_55_P2350022	3.5	3.2.E-02	*Akap6*	NM_198111
A_66_P107231	3.5	4.8.E-02	*Loxl4*	NM_001164311
A_52_P502849	3.5	4.6.E-03	*N/A*	BC108385
A_55_P1968600	3.5	7.2.E-03	*N/A*	N/A
A_66_P105032	3.5	1.0.E-02	*Gm13889*	NM_001145034
A_51_P515965	3.5	3.0.E-03	*Nfe2*	NM_008685
A_55_P2030756	3.5	1.5.E-02	*Nedd4l*	NM_031881
A_52_P165455	3.5	4.9.E-02	*Wwp1*	NM_177327
A_51_P110381	3.5	7.4.E-03	*Cd207*	NM_144943
A_55_P2115955	3.5	1.3.E-02	*Raet1e*	NM_198193
A_55_P1982201	3.5	2.4.E-03	*Sgsm2*	NM_197943
A_52_P429876	3.4	1.3.E-02	*Tbx20*	NM_194263
A_51_P386539	3.4	1.4.E-02	*Rnf125*	NM_026301
A_55_P1973033	3.4	7.8.E-03	*Ltbp1*	NM_019919
A_55_P2137077	3.4	4.7.E-03	*N/A*	AK139759
A_55_P2176266	3.4	8.8.E-03	*N/A*	N/A
A_55_P2169669	3.4	4.3.E-03	*Csf1*	NM_001113530
A_55_P2033362	3.4	7.3.E-03	*Egr2*	NM_010118
A_55_P2004447	3.4	8.0.E-03	*Fgl1*	NM_145594
A_51_P359603	3.4	1.7.E-02	*Itgb7*	NM_013566
A_55_P2181928	3.4	8.5.E-03	*Slc45a3*	NM_145977
A_55_P1957459	3.4	1.9.E-02	*Lilrb4*	NM_013532
A_66_P139683	3.4	1.2.E-02	*Zbp1*	NM_021394
A_55_P2071970	3.4	1.3.E-02	*Nav2*	NM_175272
A_51_P164296	3.3	6.3.E-03	*Adamdec1*	NM_021475

A_55_P1965659	3.3	2.2.E-02	*Havcr2*	NM_134250
A_66_P119376	3.3	4.1.E-03	*Kctd12*	NM_177715
A_55_P2192662	3.3	1.1.E-02	*Lepr*	NM_001122899
A_52_P765764	3.3	1.9.E-02	*N/A*	AF316510
A_52_P102248	3.3	1.9.E-02	*Mex3b*	NM_175366
A_55_P2334058	3.3	2.1.E-02	*4930401C15Ril*	AK015032
A_51_P391454	3.3	9.1.E-03	*Il7*	NM_008371
A_51_P121915	3.3	6.5.E-03	*BC089597*	NM_145424
A_55_P2175146	3.3	2.8.E-02	*N/A*	N/A
A_52_P305053	3.3	1.1.E-02	*B330016D10Ri*	NR_030695
A_55_P2036007	3.3	4.6.E-03	*Rai2*	NM_198409
A_51_P368496	3.3	6.5.E-03	*Tmem98*	NM_029537
A_55_P2175579	3.3	1.2.E-02	*Olfr1466*	NM_146694
A_55_P2098598	3.3	4.7.E-03	*Btg1*	NM_007569
A_55_P2008016	3.3	1.3.E-02	*Armc3*	NM_001081083
A_51_P157083	3.3	3.9.E-03	*Gas1*	NM_008086
A_51_P317505	3.3	1.5.E-02	*Nat1*	NM_008673
A_55_P1978681	3.3	3.0.E-02	*Tspan8*	NM_146010
A_55_P2039044	3.3	4.6.E-03	*Cyp3a59*	NM_001105160
A_52_P386627	3.3	6.6.E-03	*Irak3*	NM_028679
A_55_P2072661	3.2	4.2.E-03	*Prex1*	NM_177782
A_51_P176156	3.2	1.4.E-02	*LOC10003894*	NM_001173459
A_51_P446512	3.2	2.6.E-02	*2310016G11Ril*	XM_001003421
A_55_P2137611	3.2	1.9.E-02	*Irgm2*	NM_019440
A_51_P479818	3.2	1.3.E-02	*Lonrf3*	NM_028894
A_51_P222475	3.2	4.2.E-03	*Ehbp1l1*	NM_053252
A_55_P2003221	3.2	2.1.E-02	*Gimap3*	NM_031247
A_52_P594756	3.2	4.5.E-03	*Asb4*	NM_023048
A_55_P1990495	3.2	4.5.E-03	*Stat5a*	NM_011488
A_55_P2045451	3.2	1.3.E-02	*Olfr906*	NM_146803
A_52_P215170	3.2	1.5.E-02	*Acot4*	NM_134247
A_52_P161488	3.2	5.3.E-03	*Clec4e*	NM_019948
A_52_P458279	3.2	3.6.E-03	*Prlr*	NM_011169
A_55_P1974845	3.2	6.1.E-03	*Pde1a*	NM_001009978
A_55_P2123841	3.2	1.4.E-02	*Zfhx4*	NM_030708
A_51_P112355	3.2	2.2.E-02	*Igtp*	NM_018738
A_55_P2133225	3.2	3.9.E-03	*Mmgt2*	NM_175002
A_55_P2164085	3.2	1.9.E-02	*Raet1a*	NM_009016
A_55_P2257670	3.2	5.5.E-03	*A030001D16Ri*	AK037146
A_55_P2003483	3.2	7.9.E-03	*Gldc*	NM_138595
A_52_P330289	3.2	1.3.E-02	*Inpp4b*	NM_001024617

A_52_P382149	3.2	2.9.E-02	*Cyp26a1*	NM_007811
A_51_P108459	3.2	1.5.E-02	*Gpr65*	NM_008152
A_51_P181565	3.1	9.4.E-03	*Hbegf*	NM_010415
A_55_P2013236	3.1	2.5.E-02	*S100g*	NM_009789
A_55_P2148071	3.1	1.2.E-02	*N/A*	N/A
A_55_P2185178	3.1	2.4.E-02	*Tmem28*	N/A
A_51_P215077	3.1	4.6.E-03	*Mgst3*	NM_025569
A_55_P1984830	3.1	1.0.E-02	*Fgl1*	NM_145594
A_55_P1955325	3.1	1.2.E-02	*Zfp820*	NM_029281
A_52_P298165	3.1	3.3.E-02	*N/A*	XR_106194
A_55_P2061779	3.1	7.3.E-03	*Zfp456*	NM_001001186
A_51_P235801	3.1	1.7.E-02	*Zfp36l1*	NM_007564
A_55_P1956179	3.1	2.7.E-02	*Git2*	NM_019834
A_55_P1959228	3.1	1.5.E-02	*Slc4a4*	NM_018760
A_55_P2014882	3.1	8.6.E-03	*Dhrs9*	NM_175512
A_51_P144349	3.1	9.1.E-03	*Dtx4*	NM_172442
A_55_P2043718	3.1	1.4.E-02	*Cacna1f*	NM_019582
A_55_P2119772	3.1	1.2.E-02	*Scn3a*	NM_018732
A_55_P2170034	3.1	7.3.E-03	*Rgl1*	NM_016846
A_55_P2150734	3.0	1.5.E-02	*N/A*	JF690609
A_55_P2194958	3.0	1.6.E-02	*Nfia*	NM_010905
A_51_P152797	3.0	2.0.E-02	*2810039B14Ril*	XR_105719
A_55_P2039541	3.0	1.1.E-02	*Arhgap15*	NM_153820
A_55_P1969416	3.0	9.5.E-03	*Sntg1*	NM_027671
A_55_P1967776	3.0	1.1.E-02	*Slc4a4*	NM_018760
A_51_P286488	3.0	2.8.E-02	*Ier3*	NM_133662
A_51_P151126	3.0	6.7.E-03	*Cd52*	NM_013706
A_52_P476731	3.0	8.1.E-03	*Fam110c*	NM_027828
A_55_P2052897	3.0	1.0.E-02	*Arhgap30*	NM_001005508
A_55_P2081116	3.0	5.8.E-03	*Fam89a*	NM_001081120
A_55_P2176325	3.0	8.9.E-03	*Prex1*	NM_177782
A_52_P240542	3.0	4.6.E-02	*Id2*	NM_010496
A_55_P1987186	3.0	2.6.E-02	*Ttll9*	NM_001083618
A_55_P1957249	3.0	7.9.E-03	*Pdgfrb*	NM_001146268
A_55_P2032966	3.0	4.7.E-03	*Hmgcs1*	NM_145942
A_55_P2032388	3.0	1.6.E-02	*Trim24*	NM_145076
A_66_P112305	3.0	1.5.E-02	*Myo1f*	NM_053214
A_55_P2148171	3.0	1.9.E-02	*A330049M08Ri*	NM_001168500
A_51_P337125	3.0	1.3.E-02	*Inpp5d*	NM_010566
A_51_P382849	3.0	5.3.E-03	*Emb*	NM_010330
A_51_P352594	3.0	7.8.E-03	*St5*	NM_001001326

A_55_P1972720	3.0	5.0.E-03	*Pmm1*	NM_013872
A_51_P219888	3.0	7.3.E-03	*Olfr599*	NM_146731
A_51_P497882	3.0	3.4.E-02	*Creb3l4*	NM_030080
A_51_P265806	3.0	2.0.E-02	*Clca2*	NM_030601
A_51_P292008	3.0	2.6.E-02	*Gpx3*	NM_008161
A_55_P1954643	3.0	3.1.E-02	*2310081J21Rik*	AK036683
A_55_P2098734	3.0	9.9.E-03	*Ttc21b*	NM_001047604
A_55_P1953723	3.0	1.4.E-02	*Rab13*	AK080805
A_55_P2027653	3.0	8.3.E-03	*Arhgap25*	NM_001037727
A_51_P424532	3.0	9.3.E-03	*Vnn1*	NM_011704
A_55_P2018611	3.0	1.8.E-02	*Igfbp7*	NM_001159518
A_55_P1978424	3.0	1.7.E-02	*Bcl2a1d*	NM_007536
A_55_P2026748	3.0	1.0.E-02	*Zmat1*	NM_175446
A_52_P48681	2.9	2.8.E-02	*Cldn1*	NM_016674
A_51_P195506	2.9	7.7.E-03	*Csf1*	NM_007778
A_51_P371051	2.9	5.3.E-03	*Glipr1*	NM_028608
A_66_P107578	2.9	8.7.E-03	*Nlrp9c*	AY360472
A_55_P1969506	2.9	1.5.E-02	*Slc17a1*	NM_009198
A_55_P2072035	2.9	1.4.E-02	*Thy1*	NM_009382
A_55_P1962661	2.9	1.2.E-02	*Cyp2c67*	NM_001024719
A_55_P2094611	2.9	5.7.E-03	*N/A*	N/A
A_51_P446825	2.9	1.0.E-02	*6430573F11Rik*	NM_176952
A_55_P1978502	2.9	9.6.E-03	*H2-Q1*	NM_010390
A_55_P2034655	2.9	7.8.E-03	*N/A*	AK007918
A_51_P335770	2.9	1.2.E-02	*Afap1*	NM_027373
A_52_P350537	2.9	3.6.E-02	*Mtmr11*	NM_181409
A_55_P1984391	2.9	4.0.E-02	*Depdc5*	NM_177786
A_66_P106661	2.9	4.2.E-03	*Slc7a1*	NM_007513
A_55_P1974095	2.9	5.0.E-03	*N/A*	N/A
A_55_P1975045	2.9	8.4.E-03	*Sgcg*	NM_011892
A_55_P2093770	2.9	7.9.E-03	*N/A*	N/A
A_55_P2184897	2.9	9.1.E-03	*Lbr*	NM_133815
A_55_P2096917	2.9	8.1.E-03	*Mreg*	NM_001005423
A_51_P424338	2.9	5.4.E-03	*Nqo1*	NM_008706
A_55_P2126940	2.9	2.1.E-02	*N/A*	N/A
A_55_P2027083	2.9	1.1.E-02	*Kcnj10*	NM_001039484
A_52_P883557	2.9	2.3.E-02	*Slc30a10*	NM_001033286
A_55_P2072284	2.9	1.2.E-02	*6030429G01Rik*	NM_001033548
A_51_P451346	2.9	4.3.E-03	*Klf6*	NM_011803
A_51_P364485	2.9	1.0.E-02	*Tnfaip2*	NM_009396
A_52_P175028	2.9	7.7.E-03	*Cachd1*	AK046180

A_55_P1956718	2.9	8.7.E-03	*Rab38*	NM_028238
A_51_P114966	2.9	9.2.E-03	*Prss37*	NM_026317
A_55_P2472435	2.9	2.3.E-02	*Gbp3*	NM_018734
A_55_P1966194	2.9	9.5.E-03	*Plek*	NM_019549
A_51_P339540	2.9	9.3.E-03	*Cdkn1c*	NM_009876
A_55_P2004532	2.9	2.1.E-02	*C530028O21Ri*	NM_175696
A_51_P459108	2.8	6.2.E-03	*Insl6*	NM_013754
A_55_P2059586	2.8	3.3.E-02	*Fmo3*	NM_008030
A_66_P101538	2.8	5.9.E-03	*Lrrk2*	NM_025730
A_55_P1978186	2.8	6.4.E-03	*BC089597*	NM_145424
A_52_P364130	2.8	6.3.E-03	*Map3k14*	NM_016896
A_55_P2099014	2.8	6.5.E-03	*N/A*	AK143337
A_51_P377856	2.8	5.7.E-03	*Gstt3*	NM_133994
A_55_P2126695	2.8	1.9.E-02	*N/A*	XM_619114
A_55_P2022743	2.8	1.9.E-02	*Odz3*	NM_001145937
A_55_P2115836	2.8	5.1.E-03	*Acnat1*	NM_001164565
A_51_P236267	2.8	1.3.E-02	*St8sia4*	NM_009183
A_55_P2003746	2.8	2.0.E-02	*Serpinb3a*	NM_009126
A_51_P239654	2.8	3.1.E-02	*Nr4a1*	NM_010444
A_55_P2073432	2.8	8.9.E-03	*Wnt8a*	NM_009290
A_51_P293938	2.8	5.5.E-03	*Rasl11b*	NM_026878
A_51_P162955	2.8	2.0.E-02	*Serpina7*	NM_177920
A_55_P2007447	2.8	5.5.E-03	*Abhd3*	NM_134130
A_55_P2302848	2.8	1.2.E-02	*9430065F17Rik*	AK021377
A_55_P2048493	2.8	9.9.E-03	*4931440P22Ril*	NR_027955
A_55_P1983968	2.8	3.3.E-02	*Slc24a6*	NM_001177594
A_51_P432520	2.8	5.2.E-03	*Wrnip1*	NM_030215
A_55_P2043657	2.8	1.3.E-02	*Zfp677*	NM_172486
A_51_P422124	2.8	5.5.E-03	*Fam126a*	NM_053090
A_52_P332788	2.8	6.1.E-03	*C130026I21Rik*	AK016231
A_55_P2083919	2.8	1.4.E-02	*Robo2*	NM_175549
A_55_P2175318	2.8	1.8.E-02	*Dfna5*	AK155135
A_55_P2140002	2.8	9.5.E-03	*Tnfaip8*	NM_134131
A_55_P2030186	2.8	6.5.E-03	*Ifna7*	NM_008334
A_55_P2075200	2.8	7.3.E-03	*Tor3a*	NM_023141
A_51_P381260	2.8	6.2.E-03	*Fxyd5*	NM_008761
A_55_P2065074	2.8	9.1.E-03	*N/A*	XM_003084896
A_52_P491244	2.8	4.3.E-03	*Zfp287*	NM_133208
A_52_P308413	2.8	5.3.E-03	*1810011H11Ril*	NM_001163616
A_51_P326685	2.8	3.6.E-02	*Lrtm1*	NM_176920
A_51_P138923	2.8	4.7.E-02	*Peg12*	NM_013788

A_55_P2147315	2.8	9.1.E-03	*Olfr1441*	NM_146683
A_55_P1993955	2.8	1.3.E-02	*Pltp*	NM_011125
A_55_P2048767	2.8	1.2.E-02	*Efemp1*	NM_146015
A_51_P383032	2.8	1.9.E-02	*Clec4d*	NM_010819
A_55_P1994289	2.7	9.1.E-03	*Gm10791*	XM_003085113
A_55_P1974019	2.7	1.2.E-02	*Dapk1*	NM_029653
A_55_P1995195	2.7	1.4.E-02	*Fosl2*	NM_008037
A_55_P2039274	2.7	3.3.E-02	*Hspb3*	NM_019960
A_55_P2111790	2.7	1.9.E-02	*Gem*	NM_010276
A_55_P2072776	2.7	8.0.E-03	*Fpgs*	NM_010236
A_51_P505823	2.7	1.0.E-02	*Endod1*	NM_028013
A_55_P2024046	2.7	1.9.E-02	*Slc16a5*	NM_001080934
A_55_P2031068	2.7	1.4.E-02	*N/A*	N/A
A_52_P650028	2.7	2.4.E-02	*Dock10*	NM_175291
A_52_P497021	2.7	1.0.E-02	*Spred3*	NM_182927
A_55_P1962771	2.7	1.0.E-02	*Cyfip2*	NM_133769
A_55_P2032659	2.7	9.9.E-03	*Tox*	NM_145711
A_55_P2106106	2.7	8.7.E-03	*Gpr77*	NM_176912
A_51_P295315	2.7	1.1.E-02	*Ankrd2*	NM_020033
A_55_P2105858	2.7	9.1.E-03	*Atf5*	NM_030693
A_52_P322141	2.7	1.7.E-02	*Ccdc88b*	NM_001081291
A_55_P1955637	2.7	2.2.E-02	*Trp53i11*	NM_001025246
A_55_P2114133	2.7	1.8.E-02	*Garnl3*	NM_178888
A_51_P375987	2.7	2.6.E-02	*Fign*	NM_021716
A_55_P2178423	2.7	4.1.E-02	*N/A*	N/A
A_51_P142989	2.7	9.2.E-03	*Zfp429*	NM_001080941
A_52_P450934	2.7	1.0.E-02	*Paqr9*	NM_198414
A_55_P2103026	2.7	7.9.E-03	*Sema3d*	NM_028882
A_55_P2098697	2.7	1.6.E-02	*Tnfaip2*	NM_009396
A_51_P513311	2.7	8.6.E-03	*Rxrg*	NM_009107
A_52_P131353	2.7	2.3.E-02	*Camk1d*	NM_177343
A_55_P2292046	2.7	1.1.E-02	*D230018H15Ri*	AK084291
A_55_P2028568	2.7	1.1.E-02	*Gm6993*	XM_894578
A_52_P456750	2.7	1.2.E-02	*Aph1b*	NM_177583
A_52_P246698	2.7	1.9.E-02	*Fam126a*	NM_053090
A_51_P341130	2.7	6.5.E-03	*Iqgap1*	NM_016721
A_51_P143468	2.7	6.5.E-03	*Klhl26*	NM_178771
A_55_P2109857	2.7	1.1.E-02	*Rgs2*	NM_009061
A_55_P2106763	2.7	7.3.E-03	*1300014I06Rik*	NM_025831
A_55_P2293007	2.7	1.2.E-02	*AI662175*	AK139019
A_55_P2060592	2.7	1.1.E-02	*Hoxa1*	NM_010449

A_51_P230624	2.7	1.3.E-02	*2010109K11Rik*	NM_001162903
A_51_P342652	2.6	1.5.E-02	*Cd79b*	NM_008339
A_55_P2143251	2.6	8.9.E-03	*Rims3*	AK163492
A_51_P377452	2.6	7.4.E-03	*Ncf4*	NM_008677
A_51_P483324	2.6	2.7.E-02	*Ptpn22*	NM_008979
A_52_P13815	2.6	3.7.E-02	*Laptm5*	NM_010686
A_52_P596008	2.6	9.1.E-03	*Zeb2*	NM_015753
A_66_P124659	2.6	8.3.E-03	*Mark1*	NM_145515
A_51_P244303	2.6	7.9.E-03	*Smc1b*	NM_080470
A_66_P140699	2.6	1.8.E-02	*Gm6403*	AK032775
A_55_P2024366	2.6	2.2.E-02	*Raver2*	NM_183024
A_55_P1969058	2.6	2.0.E-02	*Epha7*	NM_001122889
A_55_P1960053	2.6	5.0.E-03	*Hvcn1*	NM_001042489
A_55_P2137121	2.6	8.2.E-03	*Sp140*	NM_001013817
A_55_P1982499	2.6	1.5.E-02	*Gldn*	NM_177350
A_55_P2001218	2.6	3.3.E-02	*Cd80*	NM_009855
A_55_P2023314	2.6	6.5.E-03	*Casz1*	NM_001159344
A_55_P2057283	2.6	1.2.E-02	*N/A*	AY170502
A_55_P2113210	2.6	1.0.E-02	*Cyth4*	NM_028195
A_55_P2315012	2.6	1.5.E-02	*4930458D05Rik*	XR_106287
A_55_P1984243	2.6	7.0.E-03	*Naip2*	NM_010872
A_52_P421897	2.6	2.9.E-02	*Morc4*	AK164017
A_52_P199614	2.6	1.7.E-02	*Wdfy4*	NM_001146022
A_51_P295816	2.6	9.0.E-03	*Cep192*	NM_027556
A_55_P1983588	2.6	1.1.E-02	*Pmepa1*	NM_022995
A_55_P2166501	2.6	6.9.E-03	*Cd44*	NM_009851
A_55_P2121456	2.6	1.9.E-02	*Fam43a*	NM_177632
A_52_P304128	2.6	1.1.E-02	*Mmp14*	NM_008608
A_52_P428745	2.6	5.1.E-03	*Camk2d*	NM_001025439
A_55_P1998421	2.6	1.6.E-02	*N/A*	N/A
A_55_P2093143	2.6	2.7.E-02	*Casd1*	NM_145398
A_52_P65077	2.6	3.5.E-02	*Zfp820*	NM_029281
A_52_P99848	2.6	1.7.E-02	*Pik3cd*	NM_008840
A_55_P2214348	2.6	1.7.E-02	*4930469K13Rik*	AK037310
A_51_P228295	2.6	5.5.E-03	*Mpzl1*	NM_001001880
A_55_P2171116	2.6	9.5.E-03	*Lgals3*	NM_001145953
A_51_P382152	2.6	1.5.E-02	*Procr*	NM_011171
A_51_P235123	2.6	9.4.E-03	*Nfkbie*	NM_008690
A_55_P2030752	2.6	8.7.E-03	*Nedd4l*	NM_031881
A_51_P450278	2.6	1.3.E-02	*2010003K11Rik*	NM_027237
A_55_P2085779	2.6	6.8.E-03	*Ifi27l2b*	NM_145449

A_55_P1955308	2.6	7.7.E-03	*Sirpb1a*	NM_001002898
A_51_P435968	2.6	9.9.E-03	*Tnfaip8*	NM_134131
A_55_P2036813	2.6	1.2.E-02	*Hist3h2ba*	NM_030082
A_55_P2130388	2.6	7.5.E-03	*Mical1*	NM_138315
A_55_P1994112	2.6	1.0.E-02	*Sema5b*	NM_013661
A_55_P2007601	2.5	2.1.E-02	*Sftpd*	NM_009160
A_51_P331288	2.5	2.6.E-02	*Akr1b7*	NM_009731
A_55_P1962937	2.5	1.5.E-02	*Trem2*	NM_031254
A_51_P117581	2.5	1.7.E-02	*Cables1*	NM_022021
A_55_P1999648	2.5	1.4.E-02	*Plin2*	NM_007408
A_52_P186937	2.5	6.5.E-03	*Cmpk2*	NM_020557
A_55_P1970788	2.5	7.5.E-03	*Gpr77*	NM_176912
A_52_P489295	2.5	5.3.E-03	*Adamts1*	NM_009621
A_51_P511482	2.5	1.4.E-02	*Ift57*	NM_028680
A_55_P2146177	2.5	8.0.E-03	*Cerk*	NM_145475
A_55_P2337074	2.5	2.2.E-02	*Mid1*	NM_183151
A_55_P2023637	2.5	8.1.E-03	*Prg4*	NM_021400
A_52_P299771	2.5	4.3.E-02	*Bcl2a1c*	NM_007535
A_55_P2065601	2.5	2.6.E-02	*Sytl5*	NM_177704
A_52_P451073	2.5	1.4.E-02	*Tnfrsf21*	NM_178589
A_55_P2140271	2.5	4.5.E-02	*Gm6083*	BG071895
A_51_P383340	2.5	2.2.E-02	*4930513O06Ri*	XM_136059
A_55_P2019098	2.5	1.7.E-02	*N/A*	N/A
A_51_P399071	2.5	4.2.E-02	*Anp32a*	NM_009672
A_52_P24690	2.5	3.3.E-02	*N/A*	AK038006
A_51_P163173	2.5	2.0.E-02	*Rbm12b*	NM_028226
A_55_P2074796	2.5	1.6.E-02	*Cdkn2c*	NM_007671
A_55_P2425437	2.5	2.5.E-02	*D030036P13Ri*	AK083507
A_52_P408736	2.5	2.0.E-02	*Slc16a7*	AK085398
A_55_P1960291	2.5	2.0.E-02	*Tjp3*	NM_013769
A_55_P2184189	2.5	1.3.E-02	*Ncf2*	BC003730
A_55_P2118037	2.5	7.1.E-03	*Sirpa*	NM_007547
A_52_P585124	2.5	4.9.E-02	*Cxcr4*	NM_009911
A_52_P557293	2.5	1.4.E-02	*Tmprss2*	NM_015775
A_55_P1994927	2.5	9.9.E-03	*Rab3il1*	NM_144538
A_55_P2028259	2.5	9.2.E-03	*Rhbdf2*	NM_172572
A_52_P584188	2.5	2.5.E-02	*Frat1*	NM_008043
A_52_P188261	2.5	1.9.E-02	*Camk2d*	NM_001025439
A_51_P301636	2.5	2.1.E-02	*9030409G11Ri*	NM_001109685
A_55_P2181738	2.5	1.5.E-02	*Lyz1*	NM_013590
A_55_P2051656	2.5	7.2.E-03	*Shank2*	NM_001113373

A_52_P467449	2.5	1.2.E-02	*Alox12*	NM_007440
A_55_P2068501	2.5	1.2.E-02	*4921508M14Ri*	XM_001480797
A_52_P11618	2.5	2.5.E-02	*Dusp19*	NM_024438
A_55_P1967500	2.5	2.0.E-02	*Nell1*	NM_001037906
A_51_P520966	2.5	2.5.E-02	*Icosl*	NM_015790
A_51_P511315	2.5	1.7.E-02	*Pstpip1*	NM_011193
A_55_P2055607	2.4	9.7.E-03	*Enpp1*	NM_008813
A_51_P464918	2.4	2.1.E-02	*Mefv*	NM_019453
A_51_P361492	2.4	1.5.E-02	*Pou2f1*	NM_198934
A_55_P1992099	2.4	1.7.E-02	*Ptprj*	NM_008982
A_51_P405167	2.4	6.3.E-03	*Maf*	NM_001025577
A_51_P275591	2.4	9.5.E-03	*Zfp292*	NM_013889
A_51_P183630	2.4	2.1.E-02	*Bcor*	NM_029510
A_52_P607128	2.4	2.0.E-02	*Msr1*	NM_031195
A_51_P111488	2.4	1.8.E-02	*Gfod2*	NM_027469
A_55_P2076085	2.4	1.7.E-02	*N/A*	XM_001480985
A_66_P113268	2.4	9.9.E-03	*Mme*	NM_008604
A_52_P274496	2.4	3.9.E-02	*Tspan18*	NM_183180
A_66_P123167	2.4	3.6.E-02	*Thsd7b*	AK051714
A_55_P2087840	2.4	2.5.E-02	*Gpr50*	NM_010340
A_51_P234956	2.4	1.6.E-02	*Xcl1*	NM_008510
A_55_P2027102	2.4	1.8.E-02	*Rgs3*	NM_134257
A_51_P161830	2.4	8.9.E-03	*Enpep*	NM_007934
A_55_P2021167	2.4	1.5.E-02	*Srpk1*	N/A
A_55_P1997604	2.4	2.9.E-02	*Pla2g4a*	NM_008869
A_55_P2097508	2.4	2.0.E-02	*Mcc*	NM_001085373
A_52_P139788	2.4	8.6.E-03	*Adamtsl1*	AK020115
A_52_P156190	2.4	1.1.E-02	*Ednra*	NM_010332
A_51_P281593	2.4	8.0.E-03	*Ppapdc1b*	NM_028000
A_55_P2146495	2.4	2.5.E-02	*Gm10554*	XM_003085214
A_55_P2071132	2.4	2.8.E-02	*Tnfrsf23*	NM_024290
A_55_P2072373	2.4	8.9.E-03	*Msn*	NM_010833
A_52_P488437	2.4	1.4.E-02	*Plek2*	NM_013738
A_66_P105736	2.4	1.1.E-02	*Net1*	NM_019671
A_55_P2026268	2.4	1.5.E-02	*N/A*	M34528
A_55_P2114994	2.4	2.5.E-02	*N/A*	N/A
A_51_P401668	2.4	2.7.E-02	*Laptm5*	NM_010686
A_55_P2401403	2.4	3.5.E-02	*C030046G05*	AK081287
A_55_P2032818	2.4	2.2.E-02	*Trim2*	NM_030706
A_52_P180373	2.4	8.3.E-03	*Mpeg1*	NM_010821
A_55_P2021565	2.4	1.7.E-02	*Ntf3*	NM_001164034

A_52_P435118	2.4	3.8.E-02	*Stard13*	NM_146258
A_52_P577748	2.4	7.4.E-03	*Lpxn*	NM_134152
A_51_P451588	2.4	3.0.E-02	*Plekhb1*	NM_013746
A_55_P1999102	2.4	7.4.E-03	*Pi16*	NM_023734
A_55_P1972490	2.4	3.6.E-02	*Gm4055*	BC058988
A_55_P1987620	2.4	9.1.E-03	*Cyp4a32*	NM_001100181
A_52_P134639	2.4	1.4.E-02	*Tgoln2*	NM_009444
A_55_P2005552	2.4	1.2.E-02	*Arhgef10l*	NM_172415
A_55_P2129348	2.4	1.2.E-02	*Pilra*	NM_153510
A_51_P515120	2.4	2.0.E-02	*Hs3st3a1*	NM_178870
A_52_P528600	2.4	1.2.E-02	*Hhex*	NM_008245
A_55_P2174661	2.4	1.6.E-02	*Tmsb15l*	NM_207267
A_55_P1972719	2.4	1.7.E-02	*Pmm1*	NM_013872
A_55_P2035843	2.4	1.1.E-02	*N/A*	N/A
A_55_P2091551	2.4	1.5.E-02	*Arhgap9*	NM_146011
A_55_P1959174	2.4	3.0.E-02	*Pmepa1*	NM_022995
A_55_P2051308	2.4	1.7.E-02	*Olfr1357*	NM_001011737
A_55_P1955015	2.4	1.4.E-02	*Myo1g*	NM_178440
A_55_P1963463	2.4	1.7.E-02	*Gabra3*	NM_008067
A_52_P78257	2.4	2.8.E-02	*Plekha2*	NM_031257
A_66_P124755	2.3	1.1.E-02	*Fut8*	NM_016893
A_55_P2056342	2.3	9.1.E-03	*Mgst3*	NM_025569
A_51_P335569	2.3	2.1.E-02	*Slco1a4*	NM_030687
A_55_P2040170	2.3	8.8.E-03	*Pmp22*	NM_008885
A_52_P175242	2.3	3.5.E-02	*Irs1*	NM_010570
A_66_P120612	2.3	2.0.E-02	*Vax2os1*	NR_002873
A_51_P391727	2.3	8.8.E-03	*Slc3a1*	NM_009205
A_55_P2090557	2.3	2.6.E-02	*BC046404*	NM_198861
A_55_P2066230	2.3	2.0.E-02	*Hck*	NM_001172117
A_55_P2146254	2.3	1.2.E-02	*Ifitm1*	NM_001112715
A_55_P2028986	2.3	3.6.E-02	*Lbp*	N/A
A_51_P348456	2.3	1.1.E-02	*Rest*	NM_011263
A_52_P595824	2.3	8.9.E-03	*Cpeb4*	NM_026252
A_55_P2063283	2.3	2.5.E-02	*N/A*	GQ984290
A_55_P2083014	2.3	1.2.E-02	*N/A*	FM179715
A_52_P534810	2.3	4.4.E-02	*Gm597*	NM_001013750
A_66_P101519	2.3	7.7.E-03	*Abcc9*	NM_021041
A_52_P248604	2.3	8.0.E-03	*Cdh5*	NM_009868
A_55_P2082733	2.3	2.1.E-02	*Cybb*	NM_007807
A_52_P185162	2.3	1.2.E-02	*Slc26a3*	BC037066
A_51_P262925	2.3	1.1.E-02	*1700018F24Ril*	NM_027069

A_55_P1981167	2.3	8.9.E-03	*C130026I21Rik*	NM_175219
A_55_P1971938	2.3	1.9.E-02	*Atp2b2*	NM_009723
A_52_P821	2.3	9.1.E-03	*Fli1*	NM_008026
A_51_P479321	2.3	1.1.E-02	*Acss1*	NM_080575
A_55_P1986630	2.3	1.3.E-02	*N/A*	N/A
A_52_P645862	2.3	8.6.E-03	*Agtr1a*	NM_177322
A_51_P469285	2.3	1.9.E-02	*Nrp1*	NM_008737
A_55_P2102065	2.3	2.5.E-02	*Gm10639*	NM_001122660
A_55_P2011111	2.3	1.6.E-02	*Cyp4a10*	NM_010011
A_55_P2016888	2.3	1.1.E-02	*LOC10050387[illegible]*	N/A
A_55_P2064043	2.3	7.4.E-03	*Cd44*	NM_009851
A_55_P2259456	2.3	2.8.E-02	*4933425B07Ri[illegible]*	AK016907
A_52_P469502	2.3	2.4.E-02	*Cda*	NM_028176
A_55_P2135031	2.3	3.1.E-02	*N/A*	N/A
A_55_P1999715	2.3	1.6.E-02	*N/A*	M97158
A_51_P265495	2.3	3.7.E-02	*Ly6a*	NM_010738
A_55_P2077558	2.3	1.6.E-02	*Sod3*	NM_011435
A_55_P1987914	2.3	2.1.E-02	*Map4k4*	NM_008696
A_51_P513568	2.3	2.0.E-02	*Stx11*	NM_029075
A_55_P2250246	2.3	1.6.E-02	*Taok3*	NM_001199685
A_55_P1984406	2.3	2.5.E-02	*Lrrk2*	NM_025730
A_55_P2123706	2.3	3.3.E-02	*Plekhb1*	NM_013746
A_55_P2012201	2.3	1.0.E-02	*App*	NM_001198823
A_55_P1973254	2.3	9.5.E-03	*Cmtm7*	NM_133978
A_55_P1958165	2.3	2.2.E-02	*Ms4a7*	NM_001025610
A_55_P2076631	2.3	9.6.E-03	*Gypc*	NM_001048207
A_55_P2057070	2.3	1.4.E-02	*Magix*	NM_018832
A_55_P2029581	2.3	2.5.E-02	*N/A*	N/A
A_66_P128997	2.3	1.5.E-02	*Pml*	NM_178087
A_52_P244702	2.3	4.1.E-02	*Tcf7*	NM_009331
A_55_P2099760	2.3	1.4.E-02	*N/A*	N/A
A_51_P408584	2.3	3.5.E-02	*Enpp1*	AK014707
A_55_P2135331	2.3	1.6.E-02	*Evl*	NM_001163394
A_55_P2044582	2.3	1.6.E-02	*Iglon5*	NM_001164518
A_51_P257419	2.3	8.9.E-03	*Lhx2*	NM_010710
A_52_P633883	2.3	2.5.E-02	*Zbtb6*	NM_146253
A_55_P2074381	2.3	8.6.E-03	*N/A*	N/A
A_51_P321341	2.3	1.8.E-02	*Sult1a1*	NM_133670
A_52_P471282	2.3	1.6.E-02	*Fmo4*	NM_144878
A_55_P1971951	2.3	2.1.E-02	*Cd86*	NM_019388
A_51_P223929	2.3	1.4.E-02	*Ash1l*	NM_138679

A_52_P52128	2.3	2.6.E-02	*Leo1*	NM_001039522
A_51_P491987	2.3	3.5.E-02	*Ripk3*	NM_019955
A_51_P352968	2.3	1.5.E-02	*Marcks*	NM_008538
A_55_P1963533	2.3	1.5.E-02	*Pcdhac2*	NM_001003672
A_55_P2110758	2.3	1.7.E-02	*B430306N03Ri*	NM_177083
A_55_P1968738	2.3	4.7.E-02	*Nfkb1*	NM_008689
A_52_P235861	2.3	3.5.E-02	*Peg3*	NM_008817
A_51_P333274	2.3	1.6.E-02	*Gzmb*	NM_013542
A_55_P2124976	2.3	4.0.E-02	*Grhl1*	NM_145890
A_55_P2038757	2.3	1.7.E-02	*Fblim1*	NM_133754
A_55_P1994832	2.3	2.1.E-02	*Zbtb42*	NM_001100460
A_55_P2181538	2.3	1.7.E-02	*Sult1d1*	NM_016771
A_55_P2022369	2.3	1.2.E-02	*Lbh*	NM_029999
A_52_P586141	2.3	3.3.E-02	*Adcy7*	NM_001037724
A_51_P304109	2.3	1.6.E-02	*Cyp2c39*	NM_010003
A_52_P547187	2.3	1.1.E-02	*Tab2*	NM_138667
A_51_P336049	2.3	4.9.E-02	*Krt33b*	NM_013570
A_66_P129236	2.3	1.5.E-02	*Raver1*	NM_027911
A_52_P597634	2.3	8.8.E-03	*Fzd1*	NM_021457
A_52_P662098	2.3	3.1.E-02	*Net1*	NM_019671
A_52_P630867	2.3	1.8.E-02	*Abcc4*	NM_001033336
A_66_P111224	2.3	2.7.E-02	*N/A*	N/A
A_55_P2077546	2.2	2.6.E-02	*N/A*	N/A
A_52_P93837	2.2	1.5.E-02	*Mme*	NM_008604
A_51_P461319	2.2	1.9.E-02	*Gatm*	NM_025961
A_55_P2158592	2.2	2.5.E-02	*Sirpb1a*	AK041165
A_55_P2025490	2.2	2.8.E-02	*Tnfrsf18*	NM_009400
A_51_P194658	2.2	4.0.E-02	*Gk2*	NM_010294
A_55_P2036567	2.2	1.8.E-02	*Vav1*	NM_011691
A_55_P2172396	2.2	3.5.E-02	*A530064D06Ri*	NM_178796
A_55_P2075070	2.2	1.3.E-02	*S1pr5*	NM_053190
A_52_P679101	2.2	1.7.E-02	*Tjp2*	NM_011597
A_55_P2128929	2.2	1.8.E-02	*Cc2d2a*	NM_172274
A_55_P1954577	2.2	1.4.E-02	*Sval2*	NM_032542
A_52_P648688	2.2	1.5.E-02	*Zc3h12d*	NM_172785
A_52_P325116	2.2	1.5.E-02	*Gramd1b*	BC070451
A_55_P2116054	2.2	3.1.E-02	*Caprin1*	NM_016739
A_55_P1957353	2.2	1.5.E-02	*N/A*	N/A
A_51_P133562	2.2	2.4.E-02	*Serpina6*	NM_007618
A_51_P374900	2.2	1.2.E-02	*P2ry13*	NM_028808
A_55_P2260094	2.2	2.3.E-02	*Tcf7l2*	NM_001142918

A_51_P392214	2.2	1.4.E-02	*1810048J11Rik*	NM_026327
A_52_P467389	2.2	3.6.E-02	*Slc15a3*	NM_023044
A_55_P2017759	2.2	1.9.E-02	*Evi2a*	NM_001033711
A_55_P2061565	2.2	2.2.E-02	*Zfp229*	NM_001164676
A_55_P2185826	2.2	2.2.E-02	*Cyp3a13*	NM_007819
A_51_P400543	2.2	7.8.E-03	*Aif1*	NM_019467
A_55_P2031043	2.2	1.1.E-02	*Stat5b*	NM_011489
A_55_P2033055	2.2	1.5.E-02	*Pdp1*	NM_001098230
A_55_P2054628	2.2	9.9.E-03	*Nrxn1*	NM_020252
A_55_P2053466	2.2	1.6.E-02	*N/A*	N/A
A_52_P295067	2.2	3.2.E-02	*Snn*	NM_009223
A_55_P2118268	2.2	2.7.E-02	*Chpf*	NM_001001565
A_51_P462428	2.2	2.3.E-02	*Galntl2*	AK019470
A_51_P355753	2.2	2.6.E-02	*Hic1*	NM_010430
A_55_P1983668	2.2	8.7.E-03	*Gm6377*	NM_001037917
A_55_P2006869	2.2	1.5.E-02	*Fyb*	NM_011815
A_52_P220879	2.2	1.3.E-02	*Tgm2*	NM_009373
A_55_P2019699	2.2	4.1.E-02	*Samhd1*	NM_001139520
A_55_P2019312	2.2	3.5.E-02	*Car12*	NM_178396
A_55_P1954061	2.2	4.8.E-02	*Nrxn2*	NM_001205234
A_55_P2128303	2.2	8.7.E-03	*Ids*	NM_010498
A_55_P2024689	2.2	1.9.E-02	*Rsbn1*	NM_172684
A_55_P2079560	2.2	2.4.E-02	*Lilra6*	NM_011090
A_51_P196925	2.2	1.9.E-02	*Cx3cl1*	NM_009142
A_55_P2050426	2.2	2.0.E-02	*Ankrd50*	NM_001167883
A_55_P2137309	2.2	2.5.E-02	*Kctd11*	NM_153143
A_55_P2006158	2.2	1.2.E-02	*N/A*	N/A
A_55_P2042988	2.2	1.5.E-02	*Aoah*	NM_012054
A_55_P2066299	2.2	3.2.E-02	*Gpr137b*	NM_031999
A_51_P294555	2.2	9.4.E-03	*Ifitm6*	NM_001033632
A_55_P2044257	2.2	4.8.E-02	*Crem*	NM_001110851
A_55_P2071466	2.2	1.4.E-02	*Ncf1*	NM_010876
A_55_P2118804	2.2	1.9.E-02	*2310057N15Rik*	NM_027170
A_55_P2080956	2.2	2.6.E-02	*Chka*	NM_013490
A_55_P2027979	2.2	1.2.E-02	*Impg2*	NM_174876
A_51_P309158	2.2	2.3.E-02	*Snx20*	NM_027840
A_55_P2062936	2.2	2.5.E-02	*Abca3*	NM_013855
A_52_P551856	2.2	2.5.E-02	*Rnft1*	NM_029788
A_66_P108965	2.2	1.5.E-02	*Smpdl3b*	NM_133888
A_55_P2273705	2.2	1.8.E-02	*A330044H09*	AK039451
A_51_P203182	2.2	2.1.E-02	*Apobr*	NM_138310

A_55_P2023542	2.2	1.4.E-02	*Ccr7*	NM_007719
A_55_P2162910	2.2	1.1.E-02	*Rtn1*	NM_153457
A_55_P2087265	2.2	1.6.E-02	*Ifitm1*	BC090258
A_55_P2091928	2.2	2.2.E-02	*Raet1b*	NM_009017
A_51_P462918	2.2	1.5.E-02	*Ehhadh*	NM_023737
A_51_P490747	2.2	2.8.E-02	*AI593442*	NM_178906
A_55_P1992959	2.2	1.4.E-02	*Pfkfb4*	NM_173019
A_52_P379337	2.2	2.2.E-02	*Rtn4*	NM_194054
A_55_P2141068	2.2	1.9.E-02	*Ccdc109a*	NM_001033259
A_51_P448814	2.2	1.3.E-02	*Zfp90*	NM_011764
A_55_P2033660	2.2	1.7.E-02	*Crls1*	NM_001024385
A_55_P1996314	2.2	1.7.E-02	*Amy2a5*	NM_001042711
A_55_P2113703	2.2	2.8.E-02	*Spib*	NM_019866
A_55_P2087607	2.2	3.9.E-02	*Cp*	NM_001042611
A_51_P254895	2.2	2.6.E-02	*Cyp4a10*	NM_010011
A_55_P1968703	2.2	1.2.E-02	*Gfra2*	NM_008115
A_55_P1952618	2.2	2.2.E-02	*Ear2*	NM_007895
A_51_P513776	2.2	3.3.E-02	*N/A*	AF296427
A_51_P478172	2.2	2.8.E-02	*N/A*	U07661
A_55_P1966721	2.2	1.9.E-02	*9930105H17Ril*	BC027236
A_52_P377416	2.2	1.0.E-02	*Disp1*	NM_026866
A_52_P354682	2.2	4.0.E-02	*Elovl7*	NM_029001
A_52_P539632	2.2	2.3.E-02	*Plxna3*	NM_008883
A_55_P2073705	2.2	3.3.E-02	*Tfdp2*	NM_178667
A_66_P132446	2.2	4.9.E-02	*Usp15*	NM_027604
A_55_P1958554	2.2	1.7.E-02	*Ar*	NM_013476
A_55_P2074085	2.2	1.9.E-02	*Gimap8*	NM_001077410
A_51_P389751	2.2	1.3.E-02	*Relb*	NM_009046
A_55_P2021266	2.2	1.5.E-02	*Hpse*	NM_152803
A_51_P285749	2.2	1.5.E-02	*Olfr1175-ps*	XM_621555
A_55_P2031045	2.2	1.2.E-02	*Stat5b*	NM_011489
A_55_P2170737	2.1	2.7.E-02	*Igf2bp2*	NM_183029
A_55_P2104487	2.1	1.8.E-02	*Pld1*	NM_001164056
A_51_P167360	2.1	2.9.E-02	*Ptpn7*	NM_177081
A_51_P201480	2.1	1.1.E-02	*Stat3*	NM_213659
A_66_P129800	2.1	1.9.E-02	*Rab8b*	NM_173413
A_55_P2056606	2.1	2.8.E-02	*N/A*	N/A
A_51_P408363	2.1	1.1.E-02	*Cfp*	NM_008823
A_55_P1976869	2.1	1.5.E-02	*Hsdl2*	NM_024255
A_55_P2129172	2.1	2.2.E-02	*Rab40b*	NM_139147
A_52_P359621	2.1	4.2.E-02	*Luzp1*	NM_024452

A_55_P2129000	2.1	1.5.E-02	*Cyyr1*	NM_144853
A_55_P1952235	2.1	1.2.E-02	*Spry1*	NM_011896
A_55_P2005475	2.1	2.3.E-02	*Sult1a1*	NM_133670
A_55_P2105472	2.1	2.1.E-02	*Omg*	NM_019409
A_51_P285206	2.1	4.4.E-02	*Cd3d*	NM_013487
A_51_P167292	2.1	3.9.E-02	*Chi3l3*	NM_009892
A_55_P2111770	2.1	1.0.E-02	*Klhl13*	NM_026167
A_52_P299832	2.1	1.2.E-02	*Plxnc1*	NM_018797
A_55_P2142430	2.1	2.1.E-02	*Bank1*	NM_001033350
A_66_P120567	2.1	1.5.E-02	*Anxa1*	NM_010730
A_55_P2004179	2.1	1.0.E-02	*Col2a1*	NM_001113515
A_52_P282905	2.1	1.4.E-02	*Ces1b*	NM_001081372
A_52_P487362	2.1	2.1.E-02	*Ppp4r4*	NM_028980
A_55_P1976224	2.1	1.7.E-02	*Ckb*	NM_021273
A_51_P270899	2.1	2.6.E-02	*Zfp61*	NM_009561
A_55_P2059010	2.1	4.5.E-02	*Rbp1*	NM_011254
A_55_P1981455	2.1	1.6.E-02	*N/A*	N/A
A_51_P176752	2.1	1.7.E-02	*4930483J18Ri*	NR_015603
A_51_P104418	2.1	4.1.E-02	*Dusp10*	NM_022019
A_55_P1960197	2.1	2.1.E-02	*P2ry14*	NM_133200
A_55_P2133266	2.1	1.2.E-02	*Cxx1c*	NM_028375
A_51_P288138	2.1	2.5.E-02	*Fpr2*	NM_008039
A_55_P2061737	2.1	1.6.E-02	*Tmsb4x*	NM_021278
A_55_P1989698	2.1	2.6.E-02	*Mmp11*	NM_008606
A_55_P2127884	2.1	2.1.E-02	*Btbd1*	NM_146193
A_51_P161037	2.1	2.5.E-02	*Cep170*	NM_001099637
A_51_P275496	2.1	1.7.E-02	*BC026762*	BC029971
A_66_P106497	2.1	3.7.E-02	*N/A*	U29575
A_55_P2027999	2.1	1.7.E-02	*Hk1*	NM_001146100
A_51_P389988	2.1	1.4.E-02	*Slc40a1*	NM_016917
A_55_P2009001	2.1	1.8.E-02	*Gprc5d*	NM_001205396
A_51_P297679	2.1	1.9.E-02	*Hcls1*	NM_008225
A_55_P2081488	2.1	2.6.E-02	*Pglyrp1*	NM_009402
A_51_P338878	2.1	1.3.E-02	*P2ry12*	NM_027571
A_55_P2149896	2.1	4.7.E-02	*BC046404*	NM_198861
A_55_P2051879	2.1	2.1.E-02	*4930473A06Ri*	NM_001081012
A_55_P1962305	2.1	1.7.E-02	*Plac8*	NM_139198
A_51_P240269	2.1	3.9.E-02	*Fam199x*	NM_146261
A_55_P1980421	2.1	1.1.E-02	*Wdr81*	NM_138950
A_55_P2134022	2.1	4.4.E-02	*Cftr*	NM_021050
A_52_P106766	2.1	4.7.E-02	*Gk5*	NM_177352

A_55_P2140118	2.1	1.0.E-02	*Qpct*	NM_027455
A_66_P101600	2.1	2.3.E-02	*Lamb1*	NM_008482
A_55_P1993719	2.1	1.3.E-02	*Tmem216*	NM_026798
A_51_P170911	2.1	1.7.E-02	*Ptpn9*	NM_019651
A_51_P302566	2.1	1.1.E-02	*Maob*	NM_172778
A_55_P2038747	2.1	1.6.E-02	*Ano1*	NM_178642
A_55_P1994132	2.1	1.3.E-02	*Tmem184a*	AK148454
A_55_P2050628	2.1	2.9.E-02	*Cyp4a31*	NM_201640
A_52_P527800	2.1	3.6.E-02	*Emilin2*	NM_145158
A_55_P2143446	2.1	2.1.E-02	*N/A*	N/A
A_55_P1971278	2.1	2.7.E-02	*Kdm2a*	NM_001001984
A_55_P2031989	2.1	2.8.E-02	*Rin2*	NM_028724
A_55_P2066827	2.1	4.5.E-02	*Hexim2*	NM_027658
A_55_P2031167	2.1	2.5.E-02	*Efna1*	NM_010107
A_55_P2030938	2.1	2.0.E-02	*Trim59*	NM_025863
A_55_P2093889	2.1	1.5.E-02	*Arhgef19*	NM_172520
A_66_P104422	2.1	2.9.E-02	*Ckap4*	NM_175451
A_55_P1953938	2.1	1.9.E-02	*1700057K13Ril*	NM_028540
A_51_P505493	2.1	1.5.E-02	*Elovl5*	NM_134255
A_52_P89477	2.1	3.8.E-02	*Bcl9*	AK147659
A_51_P447866	2.1	3.0.E-02	*Sash3*	NM_028773
A_52_P456640	2.1	1.2.E-02	*Fgr*	NM_010208
A_52_P240796	2.1	1.6.E-02	*Rdh16*	NM_009040
A_55_P1980262	2.1	1.0.E-02	*Cxx1b*	NM_001018063
A_55_P2148828	2.1	2.1.E-02	*Pfpl*	NM_019540
A_55_P2269894	2.1	3.9.E-02	*4833417C18Ril*	AK133925
A_55_P2134236	2.1	2.8.E-02	*Foxa2*	NM_010446
A_55_P2167347	2.1	2.9.E-02	*N/A*	X53355
A_55_P2011385	2.1	2.5.E-02	*N/A*	N/A
A_55_P2034864	2.1	1.5.E-02	*Tubb2b*	NM_023716
A_51_P319022	2.1	2.8.E-02	*Cxcr3*	NM_009910
A_55_P2079579	2.1	2.1.E-02	*Pira7*	NM_011094
A_52_P325443	2.0	1.5.E-02	*Arhgap29*	NM_172525
A_55_P1964483	2.0	1.1.E-02	*Cyp2c37*	NM_010001
A_52_P641758	2.0	1.9.E-02	*Ggct*	NM_026637
A_52_P591166	2.0	1.5.E-02	*Dpysl2*	NM_009955
A_55_P2073754	2.0	3.0.E-02	*Adam23*	NM_011780
A_55_P2113165	2.0	1.7.E-02	*Camkk1*	NM_018883
A_55_P2096314	2.0	2.0.E-02	*Rnf130*	N/A
A_55_P2170454	2.0	1.8.E-02	*Gsta2*	NM_008182
A_51_P442719	2.0	1.7.E-02	*Usp6nl*	NM_181399

A_52_P496655	2.0	4.3.E-02	*Leprotl1*	NM_026609
A_55_P2010152	2.0	1.9.E-02	*Sell*	NM_001164059
A_55_P2055257	2.0	1.4.E-02	*Maoa*	NM_173740
A_52_P441974	2.0	4.1.E-02	*Evc2*	NM_145920
A_55_P1983523	2.0	3.8.E-02	*Cd300ld*	NM_145437
A_55_P2185821	2.0	1.8.E-02	*Cyp3a13*	NM_007819
A_52_P597461	2.0	1.1.E-02	*Skil*	NM_011386
A_52_P62444	2.0	2.1.E-02	*Syn2*	NM_013681
A_55_P2048348	2.0	1.3.E-02	*Aig1*	NM_025446
A_51_P413916	2.0	1.8.E-02	*Pgrmc2*	NM_027558
A_55_P2256646	2.0	1.6.E-02	*C130078N14*	AK081811
A_52_P94521	2.0	1.4.E-02	*2510009E07Rik*	NM_001001881
A_55_P2038101	2.0	3.5.E-02	*Dock11*	NM_001009947
A_52_P408025	2.0	3.1.E-02	*Mpped2*	NM_001143683
A_55_P1961270	2.0	2.0.E-02	*Cd72*	NM_001110320
A_55_P2038767	2.0	1.2.E-02	*Fam49a*	NM_029758
A_55_P1999958	2.0	3.5.E-02	*N/A*	XM_003086868
A_51_P121891	2.0	1.7.E-02	*Rac2*	NM_009008
A_66_P124677	2.0	4.3.E-02	*Ipcef1*	NM_001170801
A_55_P1991773	2.0	2.7.E-02	*Icos*	NM_017480
A_52_P527917	2.0	1.3.E-02	*Slc16a10*	NM_028247
A_55_P2084308	2.0	1.9.E-02	*Nid1*	NM_010917
A_55_P2398788	2.0	4.1.E-02	*Rnf112*	NM_009548
A_55_P2082989	2.0	2.6.E-02	*5430435G22Rik*	NM_145509
A_55_P2061991	2.0	1.7.E-02	*Pnldc1*	NM_001034866
A_55_P1958246	2.0	2.5.E-02	*Tcof1*	NM_001198984
A_55_P2027259	2.0	3.3.E-02	*Zcchc2*	NM_001122675
A_51_P327796	2.0	2.2.E-02	*Itgb5*	NM_010580
A_55_P2030383	2.0	4.7.E-02	*Frmd4a*	NM_001177843
A_55_P1979341	2.0	1.2.E-02	*Cyba*	NM_007806
A_52_P244496	2.0	2.9.E-02	*2610101N10Rik*	NM_001114977
A_55_P2067772	2.0	2.1.E-02	*2410089E03Rik*	NM_001162906
A_55_P2030899	2.0	4.6.E-02	*N/A*	N/A
A_66_P108709	2.0	1.4.E-02	*Gm8439*	NM_001101603
A_51_P148037	2.0	2.6.E-02	*Sh3pxd2b*	NM_177364
A_52_P326150	2.0	1.9.E-02	*Samd15*	XM_138106
A_51_P490817	2.0	1.6.E-02	*Me2*	NM_145494
A_51_P211131	2.0	2.7.E-02	*Traf3ip2*	NM_134000
A_51_P282144	2.0	1.4.E-02	*Adam23*	NM_011780
A_51_P316553	2.0	1.5.E-02	*Kdr*	NM_010612
A_55_P2072493	2.0	4.0.E-02	*F8*	NM_001161374

A_51_P498882	2.0	1.3.E-02	*Cyp2c37*	NM_010001
A_66_P138137	2.0	2.6.E-02	*Pnp*	NM_013632
A_55_P2245852	2.0	2.1.E-02	*Tspan32*	NM_001128080
A_55_P2026753	2.0	1.8.E-02	*Zmat1*	NM_175446
A_55_P1966608	2.0	2.5.E-02	*LOC10050500*[illegible]	XM_003086781
A_55_P2020128	2.0	1.2.E-02	*Dhrs3*	NM_011303
A_55_P2014690	2.0	3.1.E-02	*Taok3*	N/A
A_55_P2070194	2.0	1.8.E-02	*Caprin1*	NM_016739
A_55_P2088385	2.0	1.9.E-02	*Tnnt1*	NM_011618
A_66_P118592	2.0	2.1.E-02	*Atrx*	NM_009530
A_51_P334308	2.0	2.0.E-02	*Tspan14*	NM_145928
A_55_P2158404	2.0	2.0.E-02	*Cmpk2*	NM_020557
A_52_P209311	2.0	1.3.E-02	*Snx12*	NM_001110310
A_55_P2184796	2.0	4.1.E-02	*Pcdhb18*	NM_053143
A_55_P2047155	2.0	1.9.E-02	*Meis2*	NM_001159569
A_55_P2078735	2.0	3.7.E-02	*AI662270*	NR_015519
A_55_P1960416	2.0	2.6.E-02	*Lrrc33*	NM_146069
A_55_P2177911	2.0	1.7.E-02	*Lepr*	NM_010704
A_66_P102163	2.0	1.6.E-02	*N/A*	N/A
A_55_P2088425	2.0	1.2.E-02	*Tcn2*	NM_015749
A_55_P1980180	2.0	2.9.E-02	*Tardbp*	NM_145556
A_51_P293982	2.0	2.9.E-02	*Plekho2*	NM_153119
A_66_P118600	2.0	1.5.E-02	*Lama1*	NM_008480
A_55_P1977473	2.0	1.9.E-02	*Dab2*	NM_023118
A_51_P196158	2.0	1.6.E-02	*Btd*	NM_025295
A_52_P263095	2.0	3.5.E-02	*Ahnak*	NM_009643
A_55_P2126805	2.0	1.5.E-02	*Wdr81*	NM_138950
A_55_P2098911	2.0	3.9.E-02	*Lrrcc1*	NM_028915
A_51_P119429	2.0	1.4.E-02	*Nckap1l*	NM_153505
A_52_P307961	2.0	1.8.E-02	*Abca5*	NM_147219
A_52_P354823	2.0	2.5.E-02	*Irf8*	NM_008320
A_55_P2046348	2.0	1.4.E-02	*Itpripl2*	NM_001033380
A_55_P1999269	2.0	2.8.E-02	*Adrbk1*	NM_130863
A_55_P2129316	2.0	3.1.E-02	*N/A*	N/A
A_52_P515057	2.0	1.5.E-02	*Slc25a24*	NM_172685
A_52_P563617	2.0	1.3.E-02	*Ssbp4*	NM_133772
A_55_P1970474	2.0	2.4.E-02	*Tmem67*	NM_177861
A_51_P288295	2.0	1.9.E-02	*N/A*	FM179740
A_55_P1981035	2.0	2.6.E-02	*N/A*	N/A
A_55_P2035509	2.0	1.9.E-02	*Pyhin1*	NM_175026
A_55_P1970665	2.0	3.1.E-02	*Kdm3b*	NM_001081256

A_55_P1993358	2.0	3.3.E-02	*N/A*	XR_030524
A_51_P118763	2.0	1.9.E-02	*Ahctf1*	NM_026375
A_51_P270558	2.0	4.1.E-02	*2210409D07Ril*	AK008868
A_52_P114282	2.0	2.4.E-02	*Armcx5*	NM_001009575
A_66_P105801	2.0	2.0.E-02	*Igf1r*	NM_010513
A_51_P487918	2.0	2.1.E-02	*Rinl*	NM_177158
A_52_P552589	2.0	1.8.E-02	*Map4k1*	NM_008279
A_51_P204740	2.0	2.9.E-02	*Cd34*	NM_133654
A_51_P239737	2.0	1.3.E-02	*Pigr*	NM_011082
A_66_P117251	2.0	3.8.E-02	*Snx6*	NM_026998
A_51_P219505	2.0	2.0.E-02	*Slc41a2*	NM_177388
A_55_P1995337	2.0	3.5.E-02	*Btnl5*	NR_004051
A_51_P445473	2.0	2.3.E-02	*Fut7*	NM_013524
A_52_P624107	2.0	2.8.E-02	*Gm5039*	NR_003647
A_55_P2038362	2.0	2.6.E-02	*Acot5*	NM_145444
A_55_P2364738	2.0	4.7.E-02	*Plxdc1*	NM_001163608
A_51_P126437	2.0	3.4.E-02	*Enc1*	NM_007930
A_52_P110070	2.0	1.5.E-02	*5730416F02Rik*	NR_033596
A_55_P2000833	2.0	3.3.E-02	*E2f8*	NM_001013368
A_51_P406204	2.0	3.4.E-02	*Olfr822*	NM_146671
A_51_P418420	2.0	2.3.E-02	*Ddx3x*	NM_010028
A_65_P06572	2.0	2.9.E-02	*Smad5*	NM_001164041
A_52_P17146	2.0	1.3.E-02	*Bmpr1a*	NM_009758
A_51_P349888	2.0	2.9.E-02	*Ang2*	NM_007449
A_55_P1956812	2.0	2.6.E-02	*Fam83g*	NM_178618
A_52_P277104	2.0	1.5.E-02	*Bank1*	NM_001033350
A_55_P2072980	2.0	2.1.E-02	*Spna2*	NM_001177668
A_51_P418560	2.0	1.9.E-02	*Lnx2*	NM_080795
A_55_P1992572	1.9	1.9.E-02	*Celf4*	NM_001146292
A_51_P311785	1.9	2.3.E-02	*Man2b2*	NM_008550
A_55_P1984886	1.9	1.3.E-02	*Hcst*	NM_011827
A_51_P312437	1.9	2.6.E-02	*Dhrs7*	NM_025522
A_66_P110914	1.9	2.8.E-02	*9930032O22Ril*	NM_177162
A_52_P305995	1.9	4.0.E-02	*Ammecr1*	NM_019496
A_51_P390538	1.9	3.3.E-02	*Mpeg1*	NM_010821
A_55_P2235911	1.9	3.5.E-02	*0610009E02Rik*	AK075572
A_52_P8922	1.9	2.8.E-02	*Sntb2*	NM_009229
A_55_P2229098	1.9	2.1.E-02	*LOC629206*	AK090054
A_51_P114616	1.9	1.4.E-02	*Batf*	NM_016767
A_51_P143190	1.9	2.0.E-02	*Lyl1*	NM_008535
A_51_P225427	1.9	1.9.E-02	*Pkp2*	NM_026163

A_55_P2106404	1.9	2.3.E-02	*N/A*	N/A
A_52_P367520	1.9	1.3.E-02	*Nexn*	NM_199465
A_55_P1984730	1.9	1.3.E-02	*Sgpl1*	NM_009163
A_52_P1020860	1.9	2.0.E-02	*AW112010*	NM_001177351
A_55_P2056926	1.9	2.5.E-02	*Mtss1l*	NM_198625
A_66_P136801	1.9	2.9.E-02	*Peg13*	NR_002864
A_55_P2132902	1.9	3.7.E-02	*Gprasp1*	NM_026081
A_55_P2452259	1.9	2.1.E-02	*Gls*	NM_001113383
A_55_P1976097	1.9	1.7.E-02	*Fads3*	AK162624
A_51_P246066	1.9	1.7.E-02	*Slamf9*	NM_029612
A_66_P105175	1.9	2.2.E-02	*Bche*	NM_009738
A_55_P2042356	1.9	2.7.E-02	*Rftn1*	NM_181397
A_51_P246653	1.9	2.3.E-02	*Clec7a*	NM_020008
A_51_P256384	1.9	1.7.E-02	*Atp2b2*	NM_009723
A_52_P51548	1.9	2.9.E-02	*Pard3*	NM_033620
A_55_P2064955	1.9	2.7.E-02	*Gm3728*	AK053416
A_55_P1954258	1.9	4.7.E-02	*Zfp652*	NM_201609
A_51_P215995	1.9	2.4.E-02	*Zswim6*	NM_145456
A_51_P162676	1.9	1.6.E-02	*Armc7*	NM_177778
A_66_P104296	1.9	3.3.E-02	*1700112E06Rik*	NM_028275
A_51_P389885	1.9	3.4.E-02	*Spic*	NM_011461
A_55_P2124736	1.9	2.8.E-02	*Col14a1*	NM_181277
A_55_P2035018	1.9	1.6.E-02	*Clec4n*	NM_020001
A_51_P405606	1.9	3.3.E-02	*Ndrg1*	NM_008681
A_55_P1953311	1.9	3.0.E-02	*Stx2*	NM_007941
A_55_P2128853	1.9	4.0.E-02	*Ear12*	NM_001012766
A_51_P112734	1.9	1.6.E-02	*Slc7a8*	NM_016972
A_52_P391983	1.9	3.1.E-02	*Nt5c2*	NM_029810
A_55_P1973046	1.9	2.0.E-02	*Cttnbp2nl*	NM_030249
A_52_P143477	1.9	3.8.E-02	*Tgoln1*	NM_009443
A_51_P449325	1.9	1.6.E-02	*H2-Oa*	NM_008206
A_55_P2001048	1.9	4.2.E-02	*Rom1*	NM_009073
A_55_P2110037	1.9	2.8.E-02	*Akap7*	NM_018747
A_55_P2024654	1.9	3.8.E-02	*Trrap*	NM_001081362
A_55_P1954086	1.9	2.9.E-02	*Postn*	NM_001198766
A_55_P2126391	1.9	2.8.E-02	*Srl*	NM_175347
A_55_P2067518	1.9	4.5.E-02	*Slc13a3*	NM_054055
A_52_P559975	1.9	3.1.E-02	*Cxcr2*	NM_009909
A_55_P1993463	1.9	4.1.E-02	*N/A*	N/A
A_55_P2019300	1.9	1.5.E-02	*Aim1l*	NM_001162970
A_51_P487690	1.9	3.3.E-02	*Ifi44*	NM_133871

A_55_P2108678	1.9	4.7.E-02	*N/A*	N/A
A_55_P2066173	1.9	4.8.E-02	*N/A*	S77453
A_55_P2065991	1.9	2.0.E-02	*S100a11*	NM_016740
A_52_P637282	1.9	1.5.E-02	*Wipf1*	NM_153138
A_55_P2128492	1.9	2.6.E-02	*Rap1a*	NM_145541
A_52_P305279	1.9	1.7.E-02	*Spata13*	NM_001033272
A_51_P240864	1.9	2.2.E-02	*Sppl3*	NM_029012
A_55_P2012021	1.9	2.9.E-02	*Olfr1370*	NM_146535
A_51_P334104	1.9	2.5.E-02	*Dcn*	NM_007833
A_52_P238902	1.9	3.2.E-02	*1200011M11Ri*	NM_024262
A_55_P2008860	1.9	2.5.E-02	*N/A*	AK077358
A_55_P2059958	1.9	1.8.E-02	*Snx7*	NM_029655
A_55_P2162152	1.9	3.6.E-02	*N/A*	N/A
A_55_P2410586	1.9	2.6.E-02	*9330156P08Ril*	AK034105
A_55_P2118799	1.9	2.4.E-02	*Rmi1*	NM_001168248
A_51_P388478	1.9	1.8.E-02	*Efnb1*	NM_010110
A_51_P185247	1.9	2.9.E-02	*Gdf10*	NM_145741
A_51_P205779	1.9	3.4.E-02	*Cd5l*	NM_009690
A_51_P501803	1.9	2.4.E-02	*Hoxa2*	NM_010451
A_55_P1961084	1.9	2.8.E-02	*Map3k1*	NM_011945
A_51_P343913	1.9	3.4.E-02	*Parp4*	NM_001145978
A_55_P2065866	1.9	1.9.E-02	*Cygb*	NM_030206
A_52_P550843	1.9	2.0.E-02	*Pppde1*	NM_024282
A_55_P2107347	1.9	4.9.E-02	*Trove2*	NM_013835
A_51_P423008	1.9	2.2.E-02	*Klhl7*	NM_026448
A_65_P18948	1.9	3.8.E-02	*Ppm1h*	NM_001110218
A_55_P2060922	1.9	3.9.E-02	*Unc5a*	NM_153131
A_66_P134474	1.9	2.7.E-02	*Ang3*	NM_001123394
A_55_P2043782	1.9	4.5.E-02	*Trpm1*	NM_001039104
A_55_P2011084	1.9	3.6.E-02	*LOC10004663*	XM_001476227
A_51_P264695	1.9	2.6.E-02	*Crym*	NM_016669
A_55_P2034998	1.9	2.1.E-02	*Tjp1*	NM_009386
A_55_P1986053	1.9	3.0.E-02	*Pcnx*	NM_018814
A_51_P212754	1.9	2.1.E-02	*Tgfbi*	NM_009369
A_55_P2181976	1.9	4.1.E-02	*Akap11*	NM_001164503
A_51_P519251	1.9	3.0.E-02	*Nupr1*	NM_019738
A_51_P253481	1.9	3.0.E-02	*Ces1g*	NM_021456
A_55_P2048398	1.9	2.4.E-02	*Suv420h1*	NM_001167885
A_55_P2169694	1.9	2.7.E-02	*N/A*	N/A
A_55_P2212733	1.9	4.1.E-02	*C80012*	BG079846
A_51_P222467	1.9	3.6.E-02	*Abcg1*	NM_009593

A_51_P354165	1.9	1.9.E-02	*Apcs*	NM_011318
A_55_P2090798	1.9	3.6.E-02	*Mtus1*	NM_001005863
A_55_P2151685	1.9	3.0.E-02	*Pira11*	NM_011088
A_55_P2450933	1.9	1.8.E-02	*N4bp2l2*	NM_201369
A_55_P2022678	1.9	4.8.E-02	*C1qtnf1*	NM_001204129
A_55_P1987396	1.9	2.0.E-02	*Tbl1xr1*	NM_030732
A_52_P551707	1.9	2.4.E-02	*Kdm6a*	NM_009483
A_51_P382970	1.9	3.2.E-02	*Itga9*	NM_133721
A_55_P2013833	1.8	3.4.E-02	*Ddx3x*	NM_010028
A_52_P302071	1.8	2.7.E-02	*N/A*	N/A
A_55_P2081055	1.8	4.4.E-02	*1700056E22Ril*	NM_028516
A_55_P2128672	1.8	3.5.E-02	*Ikbip*	NM_027078
A_55_P2028268	1.8	3.3.E-02	*Pik3cd*	NM_001164051
A_55_P2123381	1.8	3.5.E-02	*Fga*	NM_010196
A_55_P2142499	1.8	3.3.E-02	*N/A*	FM179586
A_51_P100625	1.8	3.0.E-02	*Apon*	NM_133996
A_55_P2051229	1.8	2.9.E-02	*Gpr132*	NM_019925
A_51_P308844	1.8	1.8.E-02	*Nrn1*	NM_153529
A_51_P489452	1.8	2.3.E-02	*Cdo1*	NM_033037
A_55_P2068233	1.8	3.3.E-02	*Phactr1*	NM_001005748
A_55_P2074972	1.8	2.0.E-02	*Zswim6*	NM_145456
A_51_P246166	1.8	2.6.E-02	*Expi*	NM_007969
A_55_P2087295	1.8	2.9.E-02	*Gm4022*	AK015147
A_55_P2182273	1.8	2.4.E-02	*N/A*	N/A
A_66_P110798	1.8	3.1.E-02	*N/A*	AK145507
A_55_P2062602	1.8	2.7.E-02	*4932438A13Ril*	NM_172679
A_55_P2107192	1.8	2.1.E-02	*N/A*	N/A
A_51_P323531	1.8	2.1.E-02	*Fam71e1*	NM_028169
A_52_P672803	1.8	2.8.E-02	*Ctsa*	NM_008906
A_55_P2046550	1.8	4.8.E-02	*Adamts8*	NM_013906
A_55_P1954216	1.8	2.1.E-02	*Ugt3a1*	NM_207216
A_55_P1999561	1.8	3.6.E-02	*Pram1*	NM_001002842
A_55_P2033282	1.8	3.0.E-02	*Cept1*	NM_133869
A_51_P249215	1.8	2.5.E-02	*Ptger2*	NM_008964
A_52_P311853	1.8	2.5.E-02	*Ddit4l*	NM_030143
A_55_P2109382	1.8	1.6.E-02	*Adora2a*	NM_009630
A_55_P1992475	1.8	2.3.E-02	*N/A*	N/A
A_52_P99888	1.8	2.0.E-02	*Cxcl16*	NM_023158
A_52_P177293	1.8	1.9.E-02	*Snx24*	NM_029394
A_52_P275627	1.8	2.6.E-02	*Kpnb1*	NM_008379
A_55_P1997837	1.8	1.9.E-02	*Osbp2*	NM_152818

A_55_P2011937	1.8	4.4.E-02	*Fignl1*	NM_001163359
A_55_P1955632	1.8	2.9.E-02	*Trp53i11*	NM_001025246
A_55_P1962011	1.8	4.5.E-02	*Clec1b*	NM_019985
A_52_P653966	1.8	2.8.E-02	*Atf7ip*	NM_019426
A_55_P1957413	1.8	2.4.E-02	*Lsp1*	NM_019391
A_55_P2067366	1.8	2.9.E-02	*Zfp827*	BC094552
A_51_P142153	1.8	1.9.E-02	*Filip1l*	NM_001040397
A_55_P2146034	1.8	2.2.E-02	*Abca4*	NM_007378
A_55_P2171658	1.8	3.2.E-02	*Stx3*	NM_152220
A_55_P1971483	1.8	2.9.E-02	*4932438A13Ri*	NM_172679
A_52_P459929	1.8	2.4.E-02	*Itga1*	NM_001033228
A_66_P107038	1.8	2.5.E-02	*Prcp*	NM_028243
A_55_P1994042	1.8	2.1.E-02	*Zbp1*	NM_001139519
A_52_P289213	1.8	4.4.E-02	*Notch2*	NM_010928
A_52_P97670	1.8	4.0.E-02	*Atad2b*	NM_001099628
A_55_P2010003	1.8	2.1.E-02	*Zfp113*	NM_019747
A_55_P2189461	1.8	3.1.E-02	*LOC10050469*	XR_104814
A_55_P2104327	1.8	3.5.E-02	*Pxdn*	NM_181395
A_52_P111963	1.8	2.9.E-02	*1810030O07Ri*	NM_175141
A_66_P105689	1.8	4.9.E-02	*Trim34a*	NM_030684
A_51_P455647	1.8	2.5.E-02	*Car2*	NM_009801
A_52_P506529	1.8	3.4.E-02	*Rabgap1l*	NM_001038621
A_55_P1954569	1.8	2.4.E-02	*Hmgcs1*	AK031297
A_55_P2109033	1.8	2.0.E-02	*Hmgcs2*	NM_008256
A_55_P2115364	1.8	4.1.E-02	*N/A*	N/A
A_51_P192089	1.8	3.3.E-02	*Pinx1*	NM_028228
A_55_P2163928	1.8	3.6.E-02	*Cxx1c*	NM_028375
A_52_P637812	1.8	2.6.E-02	*Prrg4*	NM_178695
A_55_P2000623	1.8	2.4.E-02	*Stag1*	NM_009282
A_55_P2007964	1.8	2.9.E-02	*Cx3cr1*	NM_009987
A_52_P66199	1.8	2.2.E-02	*Fbxo11*	NM_001081034
A_55_P1960097	1.8	2.1.E-02	*Epb4.1l3*	NM_013813
A_52_P465129	1.8	3.2.E-02	*N/A*	N/A
A_55_P2024461	1.8	2.5.E-02	*Tbkbp1*	NM_198100
A_55_P2005145	1.8	4.2.E-02	*Zfp455*	NM_001048204
A_51_P410918	1.8	3.9.E-02	*A830007P12Ri*	NM_146115
A_55_P2159600	1.8	2.9.E-02	*Herc1*	NM_145617
A_55_P1991219	1.8	2.5.E-02	*Stat3*	NM_011486
A_55_P1966863	1.8	3.5.E-02	*Mad2l2*	NM_027985
A_55_P2021465	1.8	1.9.E-02	*Phldb2*	NM_153412
A_51_P402193	1.8	3.5.E-02	*Map3k1*	NM_011945

A_55_P1990346	1.8	3.8.E-02	*Adamdec1*	NM_021475
A_52_P539161	1.8	4.2.E-02	*Rdh11*	NM_021557
A_55_P1960566	1.8	2.4.E-02	*Csda*	NM_139117
A_66_P108770	1.8	3.1.E-02	*Oxct1*	NM_024188
A_55_P2058942	1.8	2.6.E-02	*Aldh3b1*	NM_026316
A_55_P2024953	1.8	4.2.E-02	*Tbc1d4*	NM_001081278
A_52_P95910	1.8	2.2.E-02	*Ugcg*	NM_011673
A_51_P227392	1.8	3.2.E-02	*Rhou*	NM_133955
A_55_P2159294	1.8	2.7.E-02	*N/A*	N/A
A_55_P2048279	1.8	3.1.E-02	*Tlr13*	NM_205820
A_55_P1965892	1.8	4.1.E-02	*Gnpda1*	NM_011937
A_51_P423578	1.8	1.9.E-02	*Slfn2*	NM_011408
A_55_P2018994	1.8	3.0.E-02	*Elk3*	NM_013508
A_55_P2054420	1.8	3.1.E-02	*Tcf4*	NM_013685
A_51_P131358	1.8	3.0.E-02	*Selplg*	NM_009151
A_51_P230439	1.8	2.7.E-02	*Ppfibp2*	NM_008905
A_52_P600531	1.8	3.5.E-02	*Bahd1*	NM_001045523
A_66_P108345	1.8	2.8.E-02	*Sgcb*	NM_011890
A_55_P2097670	1.8	3.1.E-02	*N/A*	XR_035219
A_55_P2313033	1.8	2.3.E-02	*Ptprb*	NM_029928
A_55_P2113837	1.8	2.6.E-02	*Tbc1d1*	NM_019636
A_51_P307316	1.8	3.0.E-02	*Syde1*	NM_027875
A_55_P1970910	1.8	3.5.E-02	*Stard13*	NM_001163493
A_51_P410650	1.8	3.3.E-02	*Dpp9*	NM_172624
A_52_P183038	1.8	3.0.E-02	*Impdh1*	NM_011829
A_55_P2023797	1.8	3.1.E-02	*Ccdc46*	NM_029606
A_51_P354038	1.8	3.3.E-02	*Tmc6*	NM_145439
A_55_P2148321	1.8	2.0.E-02	*Gm15401*	XM_003084665
A_51_P202050	1.8	3.2.E-02	*Dtx1*	NM_008052
A_55_P2163774	1.8	3.9.E-02	*Crip1*	NM_007763
A_55_P2007249	1.8	3.0.E-02	*Spdef*	NM_013891
A_55_P1952216	1.8	3.5.E-02	*N/A*	N/A
A_55_P2001970	1.8	4.3.E-02	*6330408A02Ril*	NM_177312
A_51_P290018	1.8	5.0.E-02	*Ddx3x*	U42386
A_52_P493091	1.8	4.1.E-02	*Fmr1*	NM_008031
A_55_P2431118	1.8	5.0.E-02	*Itsn1*	NM_001110275
A_55_P1971963	1.8	3.8.E-02	*Tmem176b*	NM_023056
A_55_P2038525	1.8	2.6.E-02	*C3*	NM_009778
A_52_P680761	1.8	2.4.E-02	*Tdrd6*	NM_198418
A_52_P684138	1.8	4.7.E-02	*Dpf3*	NM_058212
A_52_P234729	1.8	3.9.E-02	*Pkd2*	NM_008861

A_55_P2119985	1.8	2.6.E-02	*Baiap2*	NM_130862
A_55_P2091985	1.8	4.2.E-02	*Gpr157*	NM_177366
A_51_P259009	1.8	2.7.E-02	*Cacnb3*	NM_007581
A_55_P2061076	1.8	4.8.E-02	*Gm3219*	NR_027380
A_55_P2169259	1.8	3.2.E-02	*Cyp3a25*	NM_019792
A_51_P104891	1.8	3.5.E-02	*Ept1*	NM_027652
A_55_P2079425	1.8	2.0.E-02	*Spred1*	NM_033524
A_51_P267239	1.8	2.7.E-02	*Litaf*	NM_019980
A_55_P2152487	1.7	4.0.E-02	*Gapdhs*	NM_008085
A_55_P1968668	1.7	3.3.E-02	*1700020O03Ri*	NM_027405
A_51_P365019	1.7	3.7.E-02	*Gclc*	NM_010295
A_51_P202033	1.7	2.6.E-02	*Wls*	NM_026582
A_52_P682745	1.7	3.4.E-02	*Dock4*	NM_172803
A_55_P2010567	1.7	4.1.E-02	*N/A*	N/A
A_55_P2341468	1.7	2.3.E-02	*Rbms3*	NM_001172123
A_51_P337230	1.7	3.0.E-02	*Galnt14*	NM_027864
A_51_P144160	1.7	3.3.E-02	*Colec10*	NM_173422
A_51_P112627	1.7	3.3.E-02	*St6galnac2*	NM_009180
A_55_P2007871	1.7	3.3.E-02	*Ptpdc1*	NM_207232
A_51_P494675	1.7	3.5.E-02	*Cotl1*	NM_028071
A_51_P234113	1.7	2.7.E-02	*Nod1*	NM_172729
A_55_P2021114	1.7	4.6.E-02	*Ier5*	NM_010500
A_52_P89425	1.7	3.0.E-02	*Pcnt*	NM_008787
A_66_P116299	1.7	2.6.E-02	*Pias1*	NM_019663
A_51_P342877	1.7	2.6.E-02	*Scn1b*	NM_011322
A_55_P2155002	1.7	3.4.E-02	*Ccdc23*	NM_001038998
A_55_P2066180	1.7	4.4.E-02	*C630004H02Ri*	NM_175454
A_55_P2078670	1.7	2.6.E-02	*Cbs*	NM_144855
A_52_P350148	1.7	3.3.E-02	*Celsr2*	NM_001004177
A_55_P1988882	1.7	2.6.E-02	*9-Sep*	NM_001113487
A_55_P1958678	1.7	3.2.E-02	*Brd2*	NM_001204973
A_66_P135165	1.7	2.5.E-02	*Nfib*	NM_001113209
A_55_P1980486	1.7	4.1.E-02	*N/A*	N/A
A_52_P681310	1.7	3.1.E-02	*Plaur*	NM_011113
A_55_P2089804	1.7	2.8.E-02	*Atf7*	NM_146065
A_51_P135340	1.7	3.8.E-02	*Panx1*	NM_019482
A_52_P70796	1.7	2.6.E-02	*Cxcr5*	NM_007551
A_51_P279100	1.7	2.5.E-02	*Ptgs1*	NM_008969
A_55_P2083988	1.7	4.5.E-02	*Lmo2*	NM_008505
A_66_P133642	1.7	4.3.E-02	*N/A*	N/A
A_51_P309740	1.7	3.6.E-02	*Usp40*	NM_001033291

A_52_P595954	1.7	4.5.E-02	*Whsc2*	NM_011914
A_51_P182993	1.7	4.7.E-02	*Map3k2*	NM_011946
A_55_P2034110	1.7	3.9.E-02	*Tgm2*	NM_009373
A_51_P394788	1.7	2.6.E-02	*Actn4*	NM_021895
A_55_P1984635	1.7	4.5.E-02	*Parp4*	NM_001145978
A_55_P2136970	1.7	4.8.E-02	*Gm7269*	XM_993534
A_52_P354744	1.7	4.0.E-02	*Slc2a3*	NM_011401
A_52_P618379	1.7	3.9.E-02	*Slc35a5*	NM_028756
A_55_P2088571	1.7	2.3.E-02	*Repin1*	NM_001079901
A_55_P2007372	1.7	3.9.E-02	*N/A*	N/A
A_52_P269158	1.7	2.6.E-02	*Pid1*	NM_001003948
A_55_P2008740	1.7	3.8.E-02	*Fcgr1*	NM_010186
A_55_P2269497	1.7	2.9.E-02	*Pdp1*	NM_001098230
A_55_P2028894	1.7	3.7.E-02	*Gata4*	NM_008092
A_51_P414653	1.7	4.2.E-02	*Plvap*	NM_032398
A_55_P2079561	1.7	2.8.E-02	*Lilra6*	NM_011090
A_55_P2008061	1.7	4.0.E-02	*Itpr2*	NM_019923
A_55_P2081530	1.7	2.9.E-02	*Sh3bgrl3*	NM_080559
A_51_P187082	1.7	4.1.E-02	*G6pdx*	NM_008062
A_55_P2081656	1.7	4.5.E-02	*N/A*	N/A
A_55_P2149931	1.7	3.5.E-02	*Arap2*	NM_178407
A_55_P1973848	1.7	2.9.E-02	*Sema4b*	NM_013659
A_55_P2136204	1.7	4.1.E-02	*Rnf38*	NM_175201
A_55_P2067533	1.7	4.7.E-02	*Foxa2*	NM_010446
A_51_P383991	1.7	4.6.E-02	*4-Sep*	NM_011129
A_52_P127130	1.7	3.8.E-02	*Msr1*	NM_031195
A_52_P6798	1.7	3.7.E-02	*Rbm22*	NM_025776
A_55_P2069306	1.7	3.0.E-02	*Ptpn18*	NM_011206
A_51_P342567	1.7	4.9.E-02	*Akap12*	NM_031185
A_51_P370350	1.7	2.9.E-02	*Galnt2*	NM_139272
A_55_P2275279	1.7	4.4.E-02	*Mid2*	NM_011845
A_55_P2108255	1.7	2.9.E-02	*Lgmn*	NM_011175
A_55_P1963724	1.7	3.5.E-02	*N/A*	N/A
A_51_P150722	1.7	4.8.E-02	*Lyst*	NM_010748
A_66_P122559	1.7	3.5.E-02	*Myct1*	NM_026793
A_55_P2023818	1.7	3.0.E-02	*Cysltr1*	NM_021476
A_55_P2129354	1.7	4.7.E-02	*Pilra*	NM_153510
A_51_P234788	1.7	3.7.E-02	*Cxxc5*	NM_133687
A_55_P2180071	1.7	2.6.E-02	*Zfand5*	NM_009551
A_55_P1983868	1.7	3.3.E-02	*4933428M09Ri*	XM_001473792
A_55_P2096827	1.7	3.1.E-02	*Art3*	NM_181728

A_55_P2093439	1.7	3.0.E-02	*Mex3d*	NM_198615
A_55_P2001920	1.7	3.1.E-02	*Flna*	NM_010227
A_51_P405476	1.7	3.9.E-02	*Fcer1g*	NM_010185
A_55_P2049572	1.7	3.9.E-02	*Pld2*	NM_008876
A_51_P363749	1.7	3.1.E-02	*Irf6*	NM_016851
A_66_P118233	1.7	3.5.E-02	*Hnrnpa0*	NM_029872
A_55_P1990324	1.7	2.8.E-02	*Ptprc*	NM_001111316
A_55_P2069231	1.7	4.8.E-02	*AI429214*	NM_001039220
A_52_P364140	1.7	4.9.E-02	*Itga5*	NM_010577
A_51_P246773	1.7	3.3.E-02	*Sesn3*	NM_030261
A_55_P2122688	1.7	4.1.E-02	*Zmym3*	NM_019831
A_55_P2106820	1.7	4.8.E-02	*N/A*	N/A
A_55_P2024041	1.7	4.5.E-02	*Psg19*	NM_011964
A_55_P2043382	1.7	3.0.E-02	*Llgl2*	NM_145438
A_55_P2154709	1.7	2.8.E-02	*Pter*	NM_008961
A_55_P1963002	1.7	4.5.E-02	*Ppfia4*	NM_001144855
A_51_P465148	1.7	3.1.E-02	*Ctsb*	NM_007798
A_51_P210082	1.7	3.1.E-02	*Ercc4*	NM_015769
A_52_P479269	1.7	3.9.E-02	*Sdc1*	NM_011519
A_55_P2125229	1.7	4.4.E-02	*Arhgef2*	NM_001198911
A_66_P118513	1.7	3.6.E-02	*11-Sep*	NM_001009818
A_55_P1988183	1.7	4.3.E-02	*Cdkn2b*	NM_007670
A_55_P2009567	1.7	4.6.E-02	*Rod1*	NM_144904
A_51_P487501	1.7	4.9.E-02	*Mobkl1b*	AK167515
A_51_P474169	1.7	3.5.E-02	*5430407P10Rik*	NM_144883
A_52_P473953	1.7	4.7.E-02	*Ctdspl*	NM_133710
A_51_P104569	1.7	4.3.E-02	*Olfr148*	NM_146505
A_52_P612382	1.7	3.8.E-02	*Cdc25b*	NM_023117
A_55_P2043987	1.7	4.3.E-02	*Luzp1*	NM_024452
A_55_P2077666	1.7	3.4.E-02	*Porcn*	NM_023638
A_55_P2007876	1.7	4.2.E-02	*Rnf144b*	NM_146042
A_51_P433026	1.7	4.2.E-02	*Ppapdc2*	NM_028922
A_55_P2133100	1.7	2.9.E-02	*Ccdc50*	NM_026202
A_55_P2060107	1.7	3.3.E-02	*Pkm2*	NM_011099
A_52_P197627	1.7	3.9.E-02	*Umps*	NM_009471
A_51_P440238	1.7	3.3.E-02	*Ggt6*	NM_027819
A_51_P382484	1.7	4.6.E-02	*Itgav*	NM_008402
A_55_P1992045	1.7	4.4.E-02	*Msi2*	NM_054043
A_55_P2148708	1.7	3.0.E-02	*N/A*	N/A
A_55_P2072631	1.7	4.0.E-02	*Prr5l*	NM_001083810
A_55_P2181341	1.7	3.6.E-02	*Ecel1*	NM_021306

A_51_P357573	1.7	3.9.E-02	*Cald1*	NM_145575
A_51_P150678	1.7	3.5.E-02	*Tnfaip8l2*	NM_027206
A_55_P2098071	1.6	3.6.E-02	*Trim25*	NM_009546
A_55_P2063661	1.6	3.9.E-02	*Tmem168*	NM_028990
A_55_P2120055	1.6	3.5.E-02	*Fgfr4*	NM_008011
A_55_P1984158	1.6	3.1.E-02	*Slc44a2*	NM_152808
A_52_P302395	1.6	3.3.E-02	*Lnpep*	NM_172827
A_52_P622442	1.6	3.2.E-02	*Pdcl3*	NM_026850
A_51_P327874	1.6	4.3.E-02	*Pth1r*	NM_011199
A_51_P371091	1.6	2.9.E-02	*Rcsd1*	NM_178593
A_55_P1964228	1.6	4.2.E-02	*Elac1*	NM_053255
A_52_P114722	1.6	4.3.E-02	*Ptpn6*	NM_001077705
A_55_P2026863	1.6	4.4.E-02	*Trim32*	NM_053084
A_55_P1994052	1.6	3.9.E-02	*Asxl1*	NM_001039939
A_51_P480928	1.6	3.3.E-02	*Zfyve16*	NM_173392
A_51_P178828	1.6	4.7.E-02	*Mbl2*	NM_010776
A_51_P132081	1.6	3.9.E-02	*Rsl1*	NM_001013769
A_51_P415809	1.6	3.8.E-02	*Tusc1*	NM_026954
A_55_P2137299	1.6	4.6.E-02	*Dnmbp*	NM_028029
A_55_P2041011	1.6	4.1.E-02	*Slc2a13*	NM_001033633
A_55_P2176753	1.6	3.2.E-02	*N/A*	N/A
A_55_P1970763	1.6	4.3.E-02	*Pilrb2*	NM_001024932
A_55_P1957633	1.6	3.5.E-02	*Gpsm3*	NM_134116
A_55_P2160094	1.6	4.4.E-02	*Plscr2*	NM_001195084
A_55_P2089310	1.6	4.1.E-02	*2900097C17Ril*	NR_024329
A_65_P15812	1.6	4.8.E-02	*Pum1*	NM_030722
A_55_P2275817	1.6	4.4.E-02	*C430042M11Ri*	AK049571
A_55_P1998506	1.6	4.2.E-02	*Aplf*	NM_001170489
A_55_P2097560	1.6	3.2.E-02	*N/A*	N/A
A_55_P1961863	1.6	3.8.E-02	*Pcdhga9*	NM_033592
A_51_P394735	1.6	4.6.E-02	*Arhgef18*	NM_133962
A_55_P1995045	1.6	4.4.E-02	*Zc3hav1*	NM_028864
A_55_P2098880	1.6	4.7.E-02	*Galnt10*	NM_134189
A_55_P2074831	1.6	4.7.E-02	*Fam168a*	NM_178764
A_55_P2130525	1.6	3.5.E-02	*2700007P21Ril*	AK165082
A_55_P2024337	1.6	4.2.E-02	*Tmem53*	NM_026837
A_55_P2183208	1.6	4.7.E-02	*Prl2c1*	NM_001045532
A_55_P2044932	1.6	4.5.E-02	*Gpr84*	NM_030720
A_55_P2162880	1.6	4.8.E-02	*Cyp3a57*	NM_001100180
A_51_P329928	1.6	4.9.E-02	*Phlda3*	NM_013750
A_51_P157255	1.6	4.8.E-02	*Sdc2*	NM_008304

A_55_P1952720	1.6	4.0.E-02	*Kctd2*	NM_183285
A_51_P156927	1.6	3.9.E-02	*Arid4a*	NM_001081195
A_52_P354298	1.6	3.5.E-02	*Ap2a2*	NM_007459
A_51_P279163	1.6	4.1.E-02	*Plcg2*	NM_172285
A_55_P1966109	1.6	4.8.E-02	*Epn2*	NM_010148
A_55_P2024530	1.6	3.6.E-02	*Siah2*	NM_009174
A_55_P2075996	1.6	3.6.E-02	*Cpne1*	NM_170588
A_55_P2130178	1.6	4.9.E-02	*Fn1*	NM_010233
A_55_P1990964	1.6	3.8.E-02	*Osbpl11*	NM_176840
A_55_P2094852	1.6	4.9.E-02	*Rabep1*	NM_019400
A_55_P1980583	1.6	4.3.E-02	*Leprot*	NM_175036
A_55_P1960187	1.6	4.3.E-02	*N/A*	N/A
A_55_P2107957	1.6	4.5.E-02	*Fam175a*	NM_172405
A_51_P124236	1.6	5.0.E-02	*Olfr381*	NM_147022
A_51_P282227	1.6	4.3.E-02	*As3mt*	NM_020577
A_55_P1989981	1.6	4.5.E-02	*Fam129b*	NM_146119
A_55_P2181752	1.6	4.1.E-02	*Apol7b*	N/A
A_51_P258409	1.6	4.1.E-02	*Hey1*	NM_010423
A_55_P2167984	1.6	4.9.E-02	*Mmd*	N/A
A_55_P2049479	1.6	4.5.E-02	*Sh3bp5*	AK165033
A_51_P127297	1.6	4.5.E-02	*Hsd11b1*	NM_008288
A_52_P491861	1.6	4.2.E-02	*Stag1*	NM_009282
A_55_P2083889	1.6	4.4.E-02	*Pea15a*	NM_011063
A_55_P2038597	1.6	4.7.E-02	*N/A*	N/A
A_52_P216613	1.6	4.4.E-02	*Gpr18*	NM_182806
A_55_P1959425	1.6	4.5.E-02	*Slc16a3*	NM_030696
A_51_P378298	1.6	4.6.E-02	*Faim3*	NM_026976
A_55_P2228918	1.6	4.4.E-02	*Rslcan18*	AK041843
A_55_P2185605	1.6	4.4.E-02	*Cd48*	NM_007649
A_51_P509971	1.6	4.6.E-02	*Plekho1*	NM_023320
A_55_P2053206	1.6	4.7.E-02	*Kdm4c*	NM_144787
A_55_P1983006	1.6	4.3.E-02	*Hdac5*	NM_001077696
A_51_P383629	1.6	4.5.E-02	*Vps4a*	NM_126165
A_55_P1962429	1.6	4.4.E-02	*Maf*	AK151738
A_55_P2102533	1.5	4.6.E-02	*Fuca2*	NM_025799
A_55_P1988246	1.5	4.6.E-02	*N/A*	N/A
A_65_P04284	1.5	4.9.E-02	*Erap1*	NM_030711
A_55_P2022364	1.5	4.5.E-02	*Nxn*	NM_008750
A_52_P556281	1.5	4.3.E-02	*G3bp2*	NM_001080795
A_55_P2130575	1.5	4.6.E-02	*N/A*	N/A
A_55_P2139181	1.5	4.7.E-02	*Cald1*	NM_145575

A_55_P2023384	1.5	4.5.E-02	*N/A*	N/A
A_55_P1982249	1.5	4.7.E-02	*Phactr2*	NM_001195065
A_55_P2033650	1.5	4.6.E-02	*Cpt2*	NM_009949
A_66_P125437	1.5	4.8.E-02	*Rpl30*	NM_009083
A_55_P2040011	1.5	4.7.E-02	*Itga7*	NM_008398
A_51_P517096	1.5	4.7.E-02	*Casp8ap2*	NM_011997

Downregulated entities

Agilent probe ID	Fold change	p-value (Corr)	Gene Symbol	Genbank Accession
A_55_P2111980	3295.7	2.8.E-06	*Hsd3b5*	NM_008295
A_55_P2152607	242.8	2.0.E-05	*Cyp4a12b*	NM_172306
A_55_P2011877	238.7	2.7.E-05	*Eif2s3y*	NM_012011
A_55_P2004606	177.1	1.1.E-03	*Serpina4-ps1*	NR_002861
A_52_P2710	152.3	1.7.E-05	*Cml5*	NM_023493
A_55_P1997003	151.7	1.7.E-03	*Serpina4-ps1*	BC031891
A_51_P402994	149.8	6.1.E-05	*Ddx3y*	NM_012008
A_55_P2151956	123.2	2.3.E-05	*N/A*	N/A
A_52_P320193	111.0	2.5.E-03	*Clec2h*	NM_053165
A_55_P2043083	99.9	1.2.E-04	*Cyp4a12a*	NM_177406
A_55_P2032081	97.9	7.1.E-05	*Dbp*	NM_016974
A_55_P2032079	52.1	3.3.E-04	*Dbp*	NM_016974
A_55_P2041350	47.5	1.4.E-04	*Cyp2d34*	NM_145474
A_55_P2078494	40.7	3.3.E-04	*Cib3*	NM_001080812
A_52_P63343	39.8	1.6.E-03	*Gm129*	NM_001033302
A_55_P1989658	35.8	2.2.E-05	*Slco1a1*	NM_013797
A_55_P1954393	30.0	8.4.E-04	*Susd4*	NM_144796
A_55_P2156219	27.1	1.5.E-03	*Gm10549*	AK141051
A_55_P2111331	25.2	6.8.E-05	*Klhl32*	NM_001163020
A_55_P2121886	23.9	1.1.E-03	*Map3k9*	NM_001174107
A_55_P2093705	23.9	4.3.E-04	*Meig1*	NM_008579
A_51_P342926	22.4	8.4.E-04	*Omd*	NM_012050
A_52_P213889	21.2	8.9.E-03	*Tmc7*	NM_172476
A_51_P456208	20.4	2.5.E-03	*Tff3*	NM_011575
A_66_P128963	19.1	2.5.E-04	*Cyp2d9*	NM_010006
A_55_P2136788	17.8	3.0.E-03	*N/A*	N/A
A_55_P2325038	17.4	6.1.E-03	*Acsm2*	NM_001177977
A_55_P2008913	17.2	7.4.E-03	*BC051665*	NM_199148
A_55_P2069679	16.9	2.0.E-04	*Olfr746*	NM_146298
A_52_P566840	16.4	1.5.E-03	*Gpr110*	NM_133776
A_55_P1971744	16.2	4.7.E-03	*4921511M17Ri*	NM_001201358

A_55_P2135153	15.4	2.3.E-03	*Lcn13*	NM_153558
A_55_P2116165	15.1	5.0.E-04	*Pfkfb3*	NM_001177753
A_51_P391897	14.3	4.3.E-04	*Olfr125*	NM_146290
A_55_P2122479	14.1	1.8.E-03	*N/A*	DV656125
A_55_P1999187	14.0	6.7.E-03	*Cdk12*	NM_026952
A_55_P2153570	13.7	3.5.E-04	*Zfp655*	NM_001083958
A_51_P188271	13.1	2.5.E-04	*Cd248*	NM_054042
A_51_P218953	13.0	2.2.E-03	*Zfp536*	NM_172385
A_52_P136914	12.9	2.2.E-03	*Nudt7*	NM_024437
A_55_P2093704	12.2	5.5.E-03	*Meig1*	NM_008579
A_55_P1985788	12.2	1.4.E-02	*Usp2*	NM_198092
A_55_P1979753	11.9	1.4.E-03	*6430411K18Rik*	NR_002848
A_55_P2033947	11.7	2.5.E-03	*Fnd3c2*	NM_001033424
A_52_P248013	11.4	1.9.E-02	*Chsy3*	NM_001081328
A_55_P1959748	11.2	1.4.E-02	*Asns*	NM_012055
A_52_P306357	11.2	1.7.E-02	*Prok1*	BC042707
A_55_P2311855	11.1	2.2.E-03	*Gm10509*	EL606049
A_55_P2086522	11.0	8.8.E-04	*Gm6797*	XM_003085249
A_55_P2107070	10.8	2.4.E-03	*Cabyr*	NM_027687
A_52_P347942	10.7	2.1.E-02	*Olfr1384*	NM_146472
A_55_P1953103	10.7	4.2.E-03	*Nudt7*	NM_024446
A_55_P2134725	10.7	1.9.E-03	*N/A*	N/A
A_51_P243418	10.7	1.6.E-02	*Clec2g*	NM_027562
A_51_P449995	10.6	4.6.E-04	*C6*	NM_016704
A_55_P1986341	10.5	8.0.E-04	*Gm4956*	NR_002858
A_55_P2103033	10.5	7.7.E-03	*N/A*	N/A
A_55_P1994147	10.1	7.0.E-03	*Zar1*	NM_174877
A_55_P2063654	10.0	3.8.E-03	*Mup20*	NM_001012323
A_52_P238175	9.9	4.2.E-03	*Odf3l1*	NM_198673
A_51_P510891	9.9	3.2.E-03	*Afp*	NM_007423
A_55_P2169247	9.8	2.0.E-04	*Gm15056*	NM_001177471
A_55_P1954116	9.6	7.3.E-03	*C8b*	NM_133882
A_55_P2152225	9.6	2.0.E-02	*Ihh*	NM_010544
A_52_P262930	9.5	9.5.E-04	*2310081J21Rik*	AK036683
A_66_P129763	9.5	1.3.E-02	*Gm6329*	AK144087
A_55_P2356263	9.4	1.7.E-02	*Anks1b*	AK036288
A_55_P2143683	9.3	1.7.E-03	*Gm9927*	AK034060
A_55_P2158663	9.3	9.1.E-03	*Cldn7*	N/A
A_51_P327451	9.2	2.8.E-04	*Alas2*	NM_009653
A_55_P2007646	9.2	8.0.E-03	*Cryaa*	NM_013501
A_52_P278497	9.1	4.7.E-03	*Eif4ebp3*	NM_201256

A_55_P2391185	9.0	8.4.E-04	*AI195381*	DV044254
A_55_P2064333	9.0	9.4.E-03	*Lama3*	NM_010680
A_55_P2092219	9.0	7.7.E-03	*Serpina9*	NM_027997
A_55_P2125791	8.9	1.0.E-03	*Gm15535*	XR_104840
A_55_P2178327	8.8	3.6.E-03	*Tll1*	NM_009390
A_55_P2091413	8.8	8.9.E-03	*N/A*	XM_978485
A_55_P2207186	8.7	6.3.E-03	*Slc30a7*	NM_023214
A_51_P324633	8.6	1.3.E-02	*Elovl3*	NM_007703
A_55_P1952935	8.6	1.2.E-02	*Olfr151*	NM_207664
A_66_P122621	8.6	4.7.E-03	*Gm9889*	AK038322
A_51_P228159	8.4	1.0.E-02	*4930430O22Ri*	XM_003085348
A_55_P2122300	8.4	8.0.E-03	*Cir1*	NM_025854
A_55_P2051476	8.4	4.7.E-03	*C8a*	NM_146148
A_55_P2019989	8.3	9.0.E-04	*N/A*	N/A
A_55_P2031668	8.3	8.4.E-04	*Gstp1*	NM_013541
A_55_P2085955	8.3	1.1.E-03	*Dnajb11*	NM_026400
A_55_P2018666	8.3	4.0.E-03	*Thrsp*	NM_009381
A_55_P2008704	8.2	5.3.E-04	*Gstp2*	NM_181796
A_51_P461429	8.1	3.3.E-03	*Cyp7b1*	NM_007825
A_55_P2023114	8.0	8.4.E-04	*Hsd3b2*	NM_153193
A_55_P1975682	8.0	5.5.E-03	*Rhox2e*	NM_001085348
A_55_P1994733	7.9	6.2.E-03	*Tmem14a*	AK017734
A_55_P2106068	7.9	7.7.E-03	*Myh2*	NM_001039545
A_55_P2058962	7.7	1.5.E-03	*Mcm10*	NM_027290
A_55_P2027708	7.7	4.7.E-03	*5830416P10Ril*	NR_028427
A_55_P1956862	7.7	5.7.E-03	*Egfr*	NM_007912
A_66_P126877	7.7	8.7.E-03	*Vmn1r214*	NM_134214
A_52_P340669	7.6	4.2.E-03	*Bhlha15*	NM_010800
A_55_P2132921	7.6	7.8.E-03	*N/A*	N/A
A_55_P2057430	7.6	6.1.E-03	*Lipn*	NM_027340
A_55_P2123012	7.5	2.2.E-02	*AA667203*	XM_003085206
A_52_P653825	7.5	9.2.E-03	*Keg1*	NM_029550
A_55_P1966635	7.5	3.8.E-03	*Vmn2r75*	NM_001102578
A_51_P228971	7.5	4.7.E-03	*Slc5a4b*	NM_023219
A_55_P2033326	7.5	4.2.E-03	*N/A*	AW012817
A_55_P2111267	7.5	8.8.E-03	*Lhx9*	NM_001042577
A_55_P2025343	7.5	1.0.E-02	*Mup21*	NM_001009550
A_55_P1955871	7.4	1.7.E-02	*Nlrp4f*	NM_175290
A_55_P1981197	7.4	7.9.E-03	*N/A*	BI247616
A_52_P259817	7.4	1.2.E-02	*Upp2*	NM_029692
A_55_P2018594	7.4	8.9.E-03	*N/A*	XM_888852

A_55_P2083474	7.4	3.0.E-02	*Lpin1*	NM_001130412
A_51_P343474	7.4	8.6.E-03	*Krt2*	NM_010668
A_55_P2075127	7.3	2.4.E-02	*Pax2*	NM_011037
A_55_P2013793	7.2	1.2.E-03	*N/A*	CF911099
A_55_P1973447	7.1	1.1.E-02	*Ybx2*	N/A
A_55_P2069760	7.1	1.1.E-02	*Sprr2b*	NM_011469
A_52_P562612	7.0	2.3.E-03	*Srd5a1*	AK082819
A_51_P151246	7.0	1.0.E-02	*Olfr924*	NM_207560
A_55_P2228122	7.0	4.3.E-03	*BC024137*	BC024137
A_51_P479230	6.9	6.9.E-04	*Nat8*	NM_023455
A_52_P514352	6.9	1.5.E-03	*Kcnk5*	NM_021542
A_66_P122730	6.9	8.0.E-03	*N/A*	AK138367
A_55_P2106241	6.9	1.8.E-03	*4930432K21Rik*	NM_029045
A_52_P329398	6.9	1.0.E-02	*Atp12a*	NM_138652
A_55_P2100281	6.8	5.0.E-03	*Defb30*	DQ141309
A_55_P2167269	6.7	1.5.E-02	*Pcsk4*	NM_008793
A_55_P1969665	6.7	1.9.E-03	*Hsd3b4*	NM_001111336
A_52_P253567	6.7	2.0.E-03	*Hsd3b6*	NM_013821
A_51_P137336	6.7	4.1.E-03	*Cdh1*	NM_009864
A_51_P184681	6.6	5.0.E-03	*Olfr675*	NM_001011848
A_51_P374464	6.6	9.5.E-04	*Gstp1*	NM_013541
A_55_P2221236	6.6	1.1.E-02	*AU024180*	AK085976
A_66_P109802	6.5	8.3.E-03	*Ces3b*	NM_001159415
A_66_P136186	6.5	1.6.E-02	*Wee1*	NM_009516
A_55_P1958798	6.5	1.7.E-03	*Mobp*	NM_008614
A_55_P1997345	6.5	2.5.E-03	*Gm11568*	NM_001205030
A_55_P2082225	6.5	6.0.E-03	*N/A*	N/A
A_55_P2201866	6.3	1.5.E-03	*2310047N11Rik*	AK019096
A_55_P2186302	6.3	2.2.E-03	*Gm9866*	XR_035276
A_55_P2097773	6.2	1.1.E-02	*Msh4*	NM_031870
A_55_P2051159	6.2	1.7.E-02	*Upp2*	NM_029692
A_51_P409637	6.2	2.1.E-03	*4930505A04Rik*	NM_001100394
A_51_P516119	6.2	8.4.E-04	*Olfr1148*	NM_001011519
A_55_P2094108	6.1	9.2.E-03	*N/A*	N/A
A_66_P130650	6.1	1.3.E-02	*Gm9979*	XM_003085861
A_55_P2095513	6.1	8.4.E-04	*Try5*	NM_001003405
A_51_P189733	6.1	8.7.E-03	*2810007J24Rik*	NM_175250
A_66_P108059	6.1	1.2.E-03	*Ttc39c*	NM_028341
A_55_P2167171	6.1	6.1.E-03	*N/A*	N/A
A_55_P2007816	6.0	2.4.E-02	*Mup4*	NM_008648
A_55_P2083186	6.0	3.8.E-03	*LOC10000961*	NM_001081452

A_55_P2055834	6.0	6.7.E-03	*N/A*	AK090145
A_55_P2177371	6.0	5.8.E-03	*Gm3086*	NR_036607
A_55_P2141801	5.9	8.9.E-03	*N/A*	N/A
A_55_P1981195	5.9	1.9.E-02	*N/A*	N/A
A_51_P223776	5.9	1.7.E-03	*Nr1d1*	NM_145434
A_66_P132787	5.9	7.9.E-03	*N/A*	M11859
A_55_P2156780	5.9	4.7.E-03	*N/A*	CJ236021
A_55_P2013470	5.9	4.3.E-03	*Gm14743*	NM_001126321
A_55_P1971139	5.9	3.5.E-03	*Fbll1*	NM_001004147
A_55_P1956863	5.9	5.4.E-03	*Egfr*	NM_007912
A_51_P420415	5.8	3.3.E-03	*Srd5a1*	NM_175283
A_55_P2086730	5.8	1.3.E-02	*Olfr910*	NM_146811
A_51_P443819	5.8	1.8.E-03	*2610034M16Ri*	NM_027001
A_55_P1989738	5.7	3.0.E-02	*4930426L09Rik*	NR_024323
A_51_P335480	5.6	5.6.E-03	*1810055G02Ri*	NM_028077
A_55_P1987117	5.5	8.0.E-03	*Slc22a16*	NM_027572
A_55_P2128511	5.5	2.1.E-03	*Ociad2*	NM_026950
A_55_P2083307	5.5	1.1.E-02	*Capn8*	NM_130890
A_52_P529660	5.5	5.0.E-03	*Lrrc52*	NM_001013382
A_55_P2005501	5.4	4.5.E-03	*Olfr392*	NM_147006
A_51_P467668	5.4	5.5.E-03	*Ick*	NM_019987
A_55_P2022778	5.4	5.7.E-03	*Trpc2*	NM_011644
A_55_P2023988	5.4	1.5.E-02	*Gm8008*	XM_001477516
A_55_P2026360	5.4	1.1.E-02	*Fbxw20*	NM_001008428
A_55_P2116674	5.4	2.8.E-02	*N/A*	N/A
A_55_P2046671	5.4	2.6.E-02	*N/A*	N/A
A_55_P2335723	5.4	6.8.E-03	*2900009J06Rik*	AA986756
A_55_P2032423	5.4	3.3.E-03	*N/A*	N/A
A_52_P275069	5.4	4.0.E-03	*Gm6792*	NM_001177416
A_55_P2092501	5.3	1.5.E-02	*Med1*	NM_134027
A_52_P150988	5.3	4.3.E-03	*Txndc11*	NM_029582
A_52_P213807	5.3	2.6.E-02	*Olfr228*	NM_146405
A_51_P211351	5.3	1.5.E-02	*Olfr677*	NM_146358
A_55_P1958227	5.3	4.2.E-03	*N/A*	AK135304
A_51_P130727	5.3	1.8.E-03	*Fkbp11*	NM_024169
A_55_P2012439	5.3	1.6.E-03	*Tnfrsf19*	NM_013869
A_66_P108979	5.2	1.4.E-02	*Olfr1535*	NM_207572
A_55_P2344593	5.2	6.5.E-03	*N/A*	AK016119
A_55_P2061104	5.2	1.7.E-02	*Mup6*	NM_001081285
A_55_P1979893	5.2	4.7.E-03	*Tef*	NM_017376
A_55_P2128113	5.1	4.3.E-03	*Tbc1d12*	NM_145952

A_52_P669682	5.1	3.0.E-02	*Gm7225*	XM_003086063
A_55_P2058761	5.1	8.2.E-03	*G6pc2*	NM_021331
A_51_P282760	5.0	4.3.E-03	*Per2*	NM_011066
A_55_P2187076	5.0	8.0.E-03	*Sncg*	NM_011430
A_55_P2129309	5.0	6.1.E-03	*Foxo3*	AK143198
A_52_P402127	5.0	3.3.E-02	*Mup9*	NM_001126319
A_66_P138178	5.0	1.4.E-02	*Gm4107*	AK041437
A_52_P628212	5.0	2.0.E-02	*Esrrb*	AK044339
A_51_P204247	5.0	7.2.E-03	*C8a*	NM_146148
A_51_P287198	4.8	9.3.E-03	*Krt23*	NM_033373
A_51_P256093	4.8	1.8.E-02	*Map2k6*	NM_011943
A_55_P2058220	4.8	5.6.E-03	*Prlhr*	NM_201615
A_51_P517145	4.8	1.2.E-02	*Sort1*	NM_019972
A_55_P2044847	4.8	2.5.E-02	*Nhlrc1*	NM_175340
A_51_P208987	4.7	1.5.E-02	*Pgm3*	NM_028352
A_52_P106259	4.7	1.7.E-02	*Egfr*	NM_207655
A_66_P120732	4.7	1.8.E-03	*Gpr45*	NM_053107
A_55_P2051044	4.7	7.4.E-03	*N/A*	N/A
A_51_P514405	4.7	1.6.E-02	*Slc2a5*	NM_019741
A_66_P106421	4.7	9.8.E-03	*Ccdc30*	NM_029286
A_52_P536494	4.6	2.1.E-02	*Mycn*	NM_008709
A_55_P2234889	4.6	5.5.E-03	*C78704*	CA882486
A_55_P2070938	4.6	5.8.E-03	*N/A*	N/A
A_55_P2000543	4.6	5.4.E-03	*Cd209f*	NM_026956
A_55_P1958804	4.6	4.2.E-03	*Hsd3b3*	NM_001161742
A_51_P122855	4.6	2.1.E-03	*Pax5*	NM_008782
A_55_P2154200	4.6	4.6.E-03	*Olfr612*	NM_001200027
A_51_P408946	4.6	3.9.E-03	*Ccne1*	NM_007633
A_51_P261164	4.6	1.5.E-02	*F2rl2*	NM_010170
A_55_P2099947	4.6	8.5.E-03	*Gdf1*	NM_001163282
A_55_P2068125	4.6	6.0.E-03	*Myct1*	NM_026793
A_51_P311904	4.6	1.7.E-03	*Ptk2b*	NM_172498
A_51_P245503	4.6	4.1.E-03	*Ugt2b1*	NM_152811
A_55_P2132800	4.5	1.2.E-02	*Zfp367*	AK041361
A_55_P2006930	4.5	9.7.E-03	*2610005L07Rik*	BC025151
A_55_P2065671	4.5	1.1.E-02	*Ccnb1*	NM_172301
A_55_P2033272	4.5	5.3.E-03	*Treh*	NM_021481
A_55_P2230154	4.5	6.1.E-03	*A730046J19Ri*	NR_040271
A_55_P1996344	4.5	2.9.E-02	*N/A*	N/A
A_55_P2118604	4.5	1.6.E-02	*St6galnac1*	NM_011371
A_51_P500661	4.4	2.6.E-03	*Rnf182*	NM_183204

A_52_P159490	4.4	1.0.E-02	*Ppp1r9a*	NM_181595
A_66_P112495	4.4	7.3.E-03	*Scn4b*	NM_001013390
A_55_P2122896	4.4	4.9.E-03	*N/A*	N/A
A_55_P2019520	4.4	4.0.E-03	*Cma2*	NM_001024714
A_52_P303891	4.4	1.9.E-03	*Nr1d2*	NM_011584
A_52_P193236	4.4	4.7.E-03	*1600002H07Ril*	NM_028056
A_55_P1961458	4.4	3.3.E-02	*Speer1-ps1*	NR_001586
A_55_P2159717	4.4	7.4.E-03	*Gm5591*	NM_001013810
A_52_P604629	4.4	5.1.E-03	*Csrnp1*	NM_153287
A_55_P2012779	4.4	7.9.E-03	*Rnf167*	NM_027445
A_55_P2147276	4.4	4.1.E-03	*Orai1*	NM_175423
A_52_P217240	4.4	3.3.E-03	*Ppme1*	NM_028292
A_55_P2053704	4.4	2.0.E-02	*Pglyrp4*	NM_001165968
A_55_P2256393	4.4	3.2.E-03	*1700030L22Rik*	BU961976
A_55_P2143851	4.4	1.1.E-02	*Gm996*	NM_001005424
A_66_P120125	4.3	3.0.E-02	*D0H4S114*	NM_053078
A_55_P2036547	4.3	2.3.E-03	*Hsd3b2*	NM_153193
A_55_P2369400	4.3	1.0.E-02	*1700066O22Ri*	NR_015541
A_55_P2122653	4.3	8.6.E-03	*Dnahc3*	BC051401
A_55_P2054342	4.3	4.5.E-03	*2810408I11Rik*	NR_038009
A_55_P2156727	4.3	1.3.E-02	*Gm16509*	XM_001473385
A_55_P2141008	4.3	1.8.E-02	*Siglech*	AK080658
A_55_P2007703	4.3	1.7.E-03	*Nanogpd*	NM_001080945
A_55_P2009988	4.3	2.3.E-03	*Trib3*	NM_175093
A_55_P2110542	4.2	3.3.E-02	*N/A*	N/A
A_51_P353524	4.2	8.0.E-03	*4930568D16Ril*	NM_029463
A_55_P2024634	4.2	1.0.E-02	*N/A*	XR_104688
A_55_P2145977	4.2	5.0.E-03	*Fbxw15*	NM_199036
A_52_P315369	4.2	1.9.E-02	*Cyb5r1*	NM_028057
A_51_P417876	4.2	6.4.E-03	*Smyd5*	NM_144918
A_52_P59711	4.2	1.7.E-02	*Fhl4*	NM_010214
A_55_P2425801	4.2	9.1.E-03	*Fmn1*	NM_010230
A_66_P125741	4.2	2.5.E-03	*Ripply2*	NM_001037907
A_55_P2157537	4.2	3.2.E-02	*Gm10866*	XM_003085086
A_55_P2061219	4.2	1.1.E-02	*Ces3a*	NM_198672
A_66_P101561	4.2	1.5.E-02	*Gm2176*	NR_028424
A_51_P486217	4.2	1.5.E-02	*Wnk4*	NM_175638
A_55_P2142222	4.2	1.2.E-02	*Serpina3h*	NR_033450
A_51_P229664	4.2	2.5.E-03	*Cd27*	NM_001033126
A_51_P429366	4.2	4.1.E-03	*Hes6*	NM_019479
A_55_P1952156	4.1	1.7.E-02	*N/A*	N/A

A_55_P2000007	4.1	1.3.E-02	*N/A*	N/A
A_51_P491667	4.1	1.2.E-02	*Derl3*	NM_024440
A_55_P2013710	4.1	1.0.E-02	*Apoc3*	N/A
A_55_P2033020	4.1	3.8.E-02	*Clcn1*	NM_013491
A_55_P2070366	4.1	4.4.E-03	*Nlrp1a*	AY355339
A_55_P2364755	4.0	1.1.E-02	*N/A*	XM_003086948
A_51_P401501	4.0	1.2.E-02	*Tmem213*	NM_029921
A_51_P189746	4.0	1.1.E-02	*Pim3*	NM_145478
A_51_P330144	4.0	4.3.E-03	*Ift140*	NM_134126
A_66_P112260	4.0	7.3.E-03	*Gm10688*	XM_003084786
A_51_P355829	4.0	1.0.E-02	*Ifna4*	NM_010504
A_55_P2246665	4.0	3.5.E-02	*4833422M21Ri*	AK014752
A_55_P2150328	4.0	9.1.E-03	*N/A*	N/A
A_52_P345539	4.0	8.9.E-03	*Uty*	NM_009484
A_52_P11402	4.0	1.6.E-02	*Lrrc24*	NM_198119
A_52_P362917	4.0	6.1.E-03	*Pfkfb3*	NM_133232
A_55_P2014021	4.0	1.2.E-02	*N/A*	N/A
A_55_P2018377	4.0	2.5.E-03	*N/A*	N/A
A_66_P127161	4.0	5.9.E-03	*Cyp2u1*	NM_027816
A_51_P224593	4.0	3.6.E-03	*Arl8a*	NM_026823
A_55_P2173288	4.0	1.1.E-02	*Pcdhb1*	NM_053126
A_55_P2422074	4.0	1.9.E-03	*BC025933*	BC025933
A_55_P2126269	3.9	9.5.E-03	*Nmb*	NM_026523
A_51_P225723	3.9	3.8.E-03	*Olfr908*	NM_146872
A_55_P1960793	3.9	5.6.E-03	*N/A*	N/A
A_55_P2000289	3.9	7.3.E-03	*Mab21l2*	NM_011839
A_55_P2116794	3.9	1.0.E-02	*N/A*	N/A
A_55_P1973926	3.9	9.6.E-03	*Ipo13*	NM_146152
A_55_P2045859	3.9	9.6.E-03	*N/A*	N/A
A_55_P2208463	3.9	8.3.E-03	*6820426E19Rik*	XR_105017
A_51_P305532	3.9	1.7.E-03	*Eif2s3x*	NM_012010
A_52_P204618	3.9	1.1.E-02	*Rnf222*	NM_177060
A_51_P250571	3.9	2.4.E-02	*Tas2r140*	NM_021562
A_55_P2104587	3.9	1.4.E-02	*N/A*	N/A
A_66_P103260	3.9	4.5.E-03	*Gm15234*	AK039813
A_55_P2406079	3.8	6.8.E-03	*AU024546*	AU024546
A_55_P2041668	3.8	1.1.E-02	*Foxl2*	NM_012020
A_55_P2179271	3.8	7.7.E-03	*Ccdc30*	NM_029286
A_51_P167843	3.8	4.7.E-03	*Timm17a*	NM_011590
A_55_P2026385	3.8	8.9.E-03	*N/A*	N/A
A_55_P2059732	3.8	9.1.E-03	*N/A*	N/A

A_52_P319093	3.8	1.9.E-02	*Serpina3k*	NM_011458
A_55_P2089840	3.8	1.1.E-02	*Eif2d*	NM_001136070
A_55_P2082454	3.8	4.9.E-03	*N/A*	N/A
A_55_P1972001	3.8	8.7.E-03	*LOC10003918*	XM_003086064
A_55_P2085518	3.8	1.6.E-02	*N/A*	N/A
A_55_P2244355	3.8	5.8.E-03	*2410024N13Ri*	AK010586
A_51_P392429	3.7	7.5.E-03	*Mrgpre*	NM_175534
A_51_P450505	3.7	1.6.E-02	*Olfr835*	NM_001012266
A_51_P185763	3.7	9.6.E-03	*Slc46a2*	NM_021053
A_55_P2033381	3.7	1.9.E-02	*1810041L15Ri*	NM_001163145
A_66_P134539	3.7	3.9.E-03	*A430110L20Ri*	XM_003084469
A_55_P2130438	3.7	4.0.E-03	*6530409C15Ri*	XM_987873
A_55_P2064741	3.7	1.1.E-02	*Nmb*	NM_026523
A_55_P2188237	3.7	5.5.E-03	*C530008M17R*	AK013453
A_55_P2130792	3.7	7.9.E-03	*Tuft1*	NM_011656
A_55_P2182467	3.7	3.9.E-03	*Slamf6*	NM_030710
A_52_P18765	3.7	1.1.E-02	*Hsbp1l1*	NM_001136181
A_55_P2036585	3.6	2.7.E-02	*Gm13547*	AK133213
A_55_P2134967	3.6	3.6.E-03	*Uty*	NM_009484
A_55_P2037930	3.6	5.3.E-03	*N/A*	N/A
A_66_P112593	3.6	6.2.E-03	*Sec61b*	NM_024171
A_51_P109258	3.6	2.5.E-02	*Cys1*	NM_138686
A_55_P2032868	3.6	3.1.E-02	*4932412H11Ri*	NM_172879
A_55_P2041082	3.6	4.3.E-03	*N/A*	N/A
A_55_P2040305	3.6	4.7.E-03	*N/A*	N/A
A_55_P1976934	3.5	3.1.E-03	*Unc13d*	BC046519
A_55_P1961408	3.5	2.1.E-02	*Gm15308*	NM_001177521
A_52_P676255	3.5	6.1.E-03	*Itpripl1*	NM_001163527
A_55_P2103703	3.5	2.0.E-02	*Mup-ps21*	XM_621624
A_55_P2067669	3.5	6.2.E-03	*Adam28*	AK081736
A_55_P2131048	3.5	9.7.E-03	*N/A*	N/A
A_55_P1989149	3.5	4.2.E-02	*Olfr394*	NM_147007
A_55_P2014249	3.5	3.3.E-02	*Sema3a*	NM_009152
A_55_P2109407	3.5	6.8.E-03	*Rnase12*	NM_001011875
A_55_P2148534	3.5	2.2.E-03	*Nr1d2*	BC096461
A_52_P278354	3.5	5.5.E-03	*Bmp7*	NM_007557
A_55_P2055112	3.5	7.9.E-03	*Prss44*	AB047758
A_55_P2030076	3.5	6.7.E-03	*Ifnab*	NM_008336
A_52_P109232	3.5	2.8.E-02	*N/A*	N/A
A_55_P1971124	3.5	6.2.E-03	*Mbd1*	NM_013594
A_55_P2378030	3.5	7.0.E-03	*4933439N14Ri*	AK019859

A_55_P2307496	3.5	7.0.E-03	*LOC620306*	AK041010
A_52_P99807	3.5	7.1.E-03	*Dpy19l3*	NM_178704
A_51_P288010	3.5	7.7.E-03	*Chrng*	NM_009604
A_55_P2038882	3.5	4.7.E-03	*Niacr1*	NM_030701
A_55_P2181176	3.5	4.7.E-03	*Nipal3*	NM_028995
A_55_P2302195	3.5	5.1.E-03	*0610040F04Rik*	AK039720
A_55_P2124712	3.4	8.0.E-03	*Ces2c*	NM_145603
A_52_P627269	3.4	7.9.E-03	*Ces2b*	NM_198171
A_55_P2125557	3.4	4.2.E-02	*Irx2*	NM_010574
A_51_P221823	3.4	8.7.E-03	*Krtap16-7*	NM_028621
A_55_P2180347	3.4	2.5.E-03	*N/A*	N/A
A_51_P214663	3.4	4.1.E-03	*Tpmt*	NM_016785
A_52_P395242	3.4	1.6.E-02	*Map2k6*	AK086722
A_55_P1971237	3.4	1.6.E-02	*Mup3*	NM_001039544
A_55_P2059864	3.4	1.5.E-02	*Igsf1*	NM_177591
A_55_P2275249	3.4	8.8.E-03	*Th*	NM_009377
A_55_P2058606	3.4	2.0.E-02	*C130079G13Ri*	NM_177661
A_66_P135403	3.4	7.3.E-03	*AI481877*	AK135748
A_55_P2073094	3.4	1.5.E-02	*Trex2*	NM_011907
A_55_P1952394	3.4	7.7.E-03	*Cphx*	NM_175342
A_55_P2076749	3.4	2.4.E-02	*Cad*	NM_023525
A_55_P2001628	3.4	9.2.E-03	*Rps4y2*	NR_003634
A_51_P444954	3.4	2.0.E-02	*Serpina5*	NM_172953
A_52_P520341	3.4	7.8.E-03	*N/A*	AK088084
A_66_P104751	3.3	2.2.E-02	*2210404J11Rik*	AK019108
A_66_P129145	3.3	3.1.E-02	*1700012O15Ri*	AK005929
A_55_P2142789	3.3	9.9.E-03	*AI463229*	XR_105529
A_55_P2089488	3.3	8.7.E-03	*Coq10b*	NM_001039710
A_55_P2154809	3.3	1.0.E-02	*Morn3*	NM_029112
A_52_P318361	3.3	7.2.E-03	*Ces2c*	NM_145603
A_55_P2152240	3.3	5.2.E-03	*Gm10383*	AK156988
A_55_P2107178	3.3	2.0.E-02	*Gm12886*	NM_001144948
A_55_P1985351	3.3	2.6.E-03	*Slc35f2*	NM_028060
A_51_P340668	3.3	4.8.E-03	*Bcl9l*	NM_030256
A_55_P2440441	3.3	4.8.E-03	*Wdhd1*	NM_172598
A_52_P15377	3.3	4.5.E-03	*Wnt9b*	NM_011719
A_52_P495869	3.3	3.1.E-02	*Mafb*	NM_010658
A_55_P2220748	3.3	7.0.E-03	*Mep1b*	NM_008586
A_55_P2037737	3.3	6.7.E-03	*N/A*	N/A
A_55_P2143623	3.3	3.7.E-02	*Vmn2r48*	NM_001105152
A_55_P2255449	3.3	2.8.E-02	*AI663975*	AK033778

A_55_P2124676	3.3	3.2.E-02	*Epm2aip1*	NM_175266
A_51_P420577	3.3	8.5.E-03	*Olfr983*	NM_146827
A_55_P2000598	3.3	6.0.E-03	*8030411F24Rik*	NM_030135
A_51_P452890	3.2	9.6.E-03	*Ssxb1*	NM_026492
A_51_P417053	3.2	1.1.E-02	*Tuft1*	NM_011656
A_51_P111455	3.2	2.6.E-02	*Wdr77*	NM_027432
A_51_P460643	3.2	1.6.E-02	*Hoxb3*	NM_001079869
A_55_P2268945	3.2	1.7.E-02	*Gm17244*	AK081449
A_66_P138469	3.2	1.7.E-02	*Gm11563*	NM_001126320
A_55_P2039514	3.2	6.5.E-03	*N/A*	N/A
A_55_P2105413	3.2	4.7.E-03	*Mug1*	NM_008645
A_55_P2103756	3.2	8.1.E-03	*Hic2*	NM_178922
A_55_P2371642	3.2	1.2.E-02	*B230104I21Rik*	XR_104907
A_51_P401343	3.2	4.9.E-03	*Cldn14*	NM_019500
A_55_P1991001	3.2	8.9.E-03	*Lman1l*	NM_199222
A_55_P2108165	3.2	1.0.E-02	*Gm6907*	NM_001164289
A_52_P294977	3.2	1.4.E-02	*Cd6*	NM_009852
A_55_P2181904	3.2	7.0.E-03	*Cd164l2*	NM_027152
A_55_P2008443	3.2	3.3.E-02	*Mybpc1*	NM_175418
A_66_P105270	3.2	7.3.E-03	*Hao1*	NM_010403
A_55_P2082519	3.2	6.5.E-03	*Olfr883*	NM_146419
A_55_P2053372	3.2	1.5.E-02	*Rbfox3*	NM_001039167
A_55_P2034033	3.2	5.0.E-03	*Il12rb1*	NM_008353
A_55_P2071731	3.2	6.1.E-03	*Olfr75-ps1*	Y15524
A_55_P2170836	3.2	5.8.E-03	*N/A*	XM_003084513
A_55_P2083257	3.2	1.5.E-02	*A3galt2*	NM_001009819
A_55_P1952404	3.2	9.1.E-03	*N/A*	N/A
A_55_P2077741	3.2	1.9.E-02	*Slc38a11*	NM_177074
A_55_P2139221	3.2	1.5.E-02	*4930594M22Ri*	XR_035095
A_55_P1995135	3.1	4.0.E-03	*Casz1*	NM_027195
A_52_P404341	3.1	8.8.E-03	*Tdo2*	NM_019911
A_55_P2004767	3.1	6.1.E-03	*Gm5591*	NM_001013810
A_51_P216702	3.1	1.1.E-02	*A130022J15Ri*	NM_175313
A_55_P2068306	3.1	8.3.E-03	*Polr1b*	AK052672
A_55_P2117559	3.1	2.6.E-02	*Siglec15*	NM_001101038
A_55_P2099363	3.1	1.0.E-02	*Stac2*	NM_146028
A_66_P117022	3.1	5.5.E-03	*Atp6v1e1*	NM_007510
A_55_P2044947	3.1	5.3.E-03	*N/A*	BY352782
A_55_P1953256	3.1	2.9.E-02	*Ctnnd1*	BC046589
A_55_P2026295	3.1	9.5.E-03	*Syne1*	NM_153399
A_51_P478881	3.1	5.3.E-03	*Ces4a*	NM_146213

A_55_P2034285	3.1	9.3.E-03	*Etnk2*	AK031665
A_52_P16752	3.1	3.8.E-03	*Aox3*	NM_023617
A_55_P2166069	3.1	8.9.E-03	*2310079G19Ri*	NM_027173
A_55_P2026125	3.1	1.2.E-02	*Srrm2*	NM_175229
A_51_P153423	3.1	1.1.E-02	*Fndc1*	NM_001081416
A_55_P1965821	3.1	5.5.E-03	*Uprt*	NM_001081189
A_55_P1967443	3.1	4.1.E-03	*N/A*	N/A
A_55_P2087345	3.1	6.5.E-03	*Htr5a*	NM_008314
A_52_P131559	3.1	2.0.E-02	*Srprb*	NM_009275
A_55_P1972040	3.1	4.1.E-03	*Nox4*	NM_015760
A_55_P2422164	3.1	2.6.E-02	*C130093G08Ri*	AI851140
A_52_P466853	3.1	4.1.E-03	*Bmyc*	NM_023326
A_55_P2054708	3.0	4.8.E-03	*N/A*	AK143362
A_55_P2266295	3.0	9.2.E-03	*Fzd3*	NM_021458
A_55_P2077263	3.0	3.8.E-03	*Cenpk*	NM_021790
A_51_P502872	3.0	1.5.E-02	*2200002D01Ri*	NM_028179
A_55_P2038126	3.0	4.5.E-03	*Tpmt*	NM_016785
A_55_P2021423	3.0	1.5.E-02	*Morn3*	NM_029112
A_55_P2175110	3.0	2.1.E-02	*Ankdd1a*	XM_357954
A_55_P2169775	3.0	1.7.E-02	*Ano3*	N/A
A_51_P455897	3.0	1.8.E-02	*Fam64a*	NM_144526
A_52_P222230	3.0	2.3.E-02	*N/A*	AK040896
A_51_P502119	3.0	9.2.E-03	*F11*	NM_028066
A_55_P2199457	3.0	1.0.E-02	*B230378P21Ri*	NR_040277
A_55_P2068560	3.0	4.0.E-03	*Lyzl4*	NM_026915
A_51_P300709	3.0	5.9.E-03	*Srm*	NM_009272
A_55_P1961400	3.0	4.2.E-03	*Grid2ip*	NM_133355
A_55_P1958185	3.0	1.6.E-02	*Hoxd11*	NM_008273
A_55_P2337706	3.0	3.0.E-02	*1110065H08Ri*	AK004374
A_55_P2186395	3.0	8.8.E-03	*Gm6340*	XM_887100
A_55_P2037919	3.0	4.1.E-03	*AA684185*	AK139675
A_55_P2141058	3.0	1.0.E-02	*LOC10050471*	NM_001201389
A_52_P272145	3.0	1.3.E-02	*N/A*	N/A
A_55_P2071344	3.0	2.1.E-02	*Anapc1*	AK054305
A_55_P2180949	2.9	7.0.E-03	*Lcp2*	AK088552
A_52_P682996	2.9	1.0.E-02	*N/A*	N/A
A_55_P2125588	2.9	5.3.E-03	*Pdgfa*	NM_008808
A_55_P1974917	2.9	1.2.E-02	*Olfr368*	NM_146374
A_55_P2186262	2.9	2.2.E-02	*2400006E01Ri*	AK019103
A_55_P2182112	2.9	4.7.E-03	*4833439L19Ri*	NM_133797
A_55_P2416494	2.9	1.7.E-02	*8430426J06Ri*	XR_105749

A_55_P2088028	2.9	1.2.E-02	*Mtss1*	NM_144800
A_55_P2086525	2.9	1.5.E-02	*N/A*	N/A
A_55_P2322620	2.9	7.0.E-03	*N/A*	AK046125
A_51_P121962	2.9	3.4.E-02	*Lphn3*	AK051766
A_55_P2109327	2.9	1.2.E-02	*Ache*	NM_009599
A_55_P2195202	2.9	7.3.E-03	*1700016K05Ril*	BU938278
A_55_P2024679	2.9	8.9.E-03	*Gm10765*	XM_003084505
A_55_P2099870	2.9	1.8.E-02	*1190007F08Rik*	NM_001163721
A_55_P2015169	2.9	8.7.E-03	*N/A*	HQ875060
A_51_P227004	2.9	7.5.E-03	*Cks1b*	NM_016904
A_55_P2007886	2.9	9.1.E-03	*Prss51*	N/A
A_55_P2127194	2.9	8.1.E-03	*N/A*	N/A
A_51_P434101	2.9	5.3.E-03	*Herpud1*	NM_022331
A_55_P2131238	2.9	1.5.E-02	*Ttc39a*	NM_153392
A_55_P2143708	2.9	1.1.E-02	*N/A*	N/A
A_55_P2183884	2.9	9.1.E-03	*Mc4r*	NM_016977
A_55_P2040250	2.9	2.5.E-02	*Raph1*	N/A
A_51_P187262	2.9	1.6.E-02	*Mmp25*	NM_001033339
A_55_P1994487	2.9	1.6.E-02	*Mamdc4*	NM_001081199
A_55_P1963006	2.9	1.0.E-02	*N/A*	N/A
A_55_P2101696	2.9	1.4.E-02	*Gnat2*	NM_008141
A_66_P114449	2.9	9.6.E-03	*Vps39*	NM_147153
A_55_P1956598	2.9	1.3.E-02	*N/A*	BC019489
A_55_P1996504	2.9	4.2.E-03	*Abcg2*	NM_011920
A_55_P2124361	2.8	1.9.E-02	*N/A*	N/A
A_55_P2088355	2.8	7.0.E-03	*N/A*	N/A
A_55_P2067747	2.8	1.0.E-02	*Psg21*	NM_027403
A_55_P1964613	2.8	2.8.E-02	*Dnttip1*	AK168315
A_55_P2029732	2.8	5.0.E-03	*C730027H18Ri*	NR_038040
A_51_P316199	2.8	2.9.E-02	*Olfr73*	NM_054090
A_66_P112024	2.8	1.3.E-02	*Gm6816*	XM_892675
A_51_P517051	2.8	1.5.E-02	*Gatsl3*	NM_028022
A_55_P2058988	2.8	1.3.E-02	*N/A*	N/A
A_55_P2347459	2.8	3.0.E-02	*1110013I04Rik*	BY726277
A_55_P2169445	2.8	8.1.E-03	*Mfsd6*	NM_133829
A_55_P1992555	2.8	4.5.E-02	*Gys2*	NM_145572
A_55_P2061495	2.8	1.0.E-02	*Cks1b*	NM_016904
A_55_P1978895	2.8	1.5.E-02	*Skint3*	NM_177578
A_55_P2024282	2.8	2.8.E-02	*Gm16441*	XM_001474528
A_52_P344290	2.8	8.0.E-03	*F2r*	NM_010169
A_55_P2361652	2.8	2.9.E-02	*C230085N15Ri*	AK088035

A_55_P2131168	2.8	1.5.E-02	*Sv2c*	NM_029210
A_66_P107437	2.8	4.9.E-02	*Sfxn5*	NM_178639
A_55_P1979019	2.8	1.5.E-02	*Etnk2*	NM_175443
A_55_P2173398	2.8	5.0.E-03	*N/A*	N/A
A_55_P2220899	2.8	3.4.E-02	*B230303O12Ri*	XR_107757
A_55_P2090025	2.8	7.1.E-03	*Mest*	NM_008590
A_55_P2290378	2.8	2.7.E-02	*AU019176*	BM228091
A_55_P2083909	2.8	4.1.E-02	*N/A*	N/A
A_55_P2206254	2.8	2.9.E-02	*LOC627096*	AK147870
A_55_P2048588	2.8	4.8.E-02	*Cdk1*	NM_007659
A_55_P1967391	2.8	3.4.E-02	*Antxr1*	AK013005
A_55_P2269700	2.8	3.5.E-02	*Ccdc163*	NM_026714
A_51_P271068	2.8	9.5.E-03	*Klf17*	NM_029416
A_55_P2160691	2.8	4.0.E-02	*N/A*	CK031974
A_55_P2050722	2.7	1.1.E-02	*N/A*	N/A
A_55_P2079569	2.7	1.5.E-02	*Gm10555*	XM_003086208
A_55_P2424921	2.7	2.9.E-02	*1300014J16Rik*	AK005005
A_55_P1968664	2.7	1.1.E-02	*N/A*	N/A
A_51_P276235	2.7	2.0.E-02	*Pnpla7*	NM_146251
A_55_P2182586	2.7	2.1.E-02	*Esrp1*	NM_194055
A_51_P162162	2.7	1.0.E-02	*Inmt*	NM_009349
A_55_P1991851	2.7	4.8.E-02	*Speg*	NM_001085370
A_55_P2120000	2.7	4.0.E-02	*N/A*	AK161418
A_55_P2091153	2.7	4.2.E-03	*BC051142*	NM_001163855
A_55_P2150392	2.7	2.9.E-02	*Cpne5*	NM_153166
A_55_P2317480	2.7	6.3.E-03	*E130104P22Ril*	AK053520
A_66_P134265	2.7	9.2.E-03	*Fam47e*	NM_001170572
A_52_P31543	2.7	3.4.E-02	*Btg2*	NM_007570
A_55_P2151675	2.7	7.0.E-03	*N/A*	AK164979
A_51_P328818	2.7	2.9.E-02	*Olfr100*	NM_207673
A_55_P2157952	2.7	5.3.E-03	*Gm10755*	XM_003086133
A_51_P408199	2.7	2.6.E-02	*Krtap4-2*	NM_026807
A_52_P278336	2.7	2.2.E-02	*Fbln5*	NM_011812
A_55_P2053714	2.7	3.1.E-02	*AA619741*	NR_033627
A_51_P201338	2.7	1.5.E-02	*Mtss1*	NM_144800
A_51_P491378	2.7	1.0.E-02	*Csn1s1*	NM_007784
A_66_P107482	2.7	5.8.E-03	*Arhgef33*	NM_001145452
A_52_P174884	2.7	3.3.E-02	*Gabrq*	NM_020488
A_55_P2006762	2.7	1.0.E-02	*Bzw1*	NM_025824
A_51_P102122	2.6	3.6.E-02	*Myog*	NM_031189
A_55_P2112419	2.6	9.5.E-03	*N/A*	N/A

A_55_P2148684	2.6	2.5.E-02	*Best1*	NM_011913
A_55_P2090254	2.6	4.4.E-02	*Sntg2*	NM_172951
A_55_P1972322	2.6	2.6.E-02	*Btg3*	NM_009770
A_55_P2061899	2.6	7.9.E-03	*N/A*	N/A
A_55_P1963915	2.6	1.1.E-02	*Gm7298*	XM_001480274
A_55_P1960366	2.6	1.1.E-02	*Fam47e*	NM_001033478
A_55_P2097763	2.6	1.9.E-02	*N/A*	N/A
A_55_P2263728	2.6	2.6.E-02	*AU045094*	AU043288
A_51_P401184	2.6	3.7.E-02	*Rarres1*	NM_001164763
A_55_P2217851	2.6	2.3.E-02	*4930453L07Rik*	AK015455
A_55_P1957038	2.6	9.5.E-03	*Gstp2*	NM_181796
A_55_P2175065	2.6	1.6.E-02	*N/A*	BC025829
A_55_P2111649	2.6	1.3.E-02	*Mbd1*	NM_013594
A_55_P2182392	2.6	9.1.E-03	*Adat3*	NM_001100606
A_51_P372550	2.6	8.6.E-03	*Cgref1*	NM_026770
A_55_P2377645	2.6	4.9.E-02	*LOC10050494*	XM_003086852
A_51_P392967	2.6	2.9.E-02	*Zmynd10*	NM_053253
A_55_P2000182	2.6	2.0.E-02	*Slc5a6*	NM_001177621
A_55_P1967227	2.6	1.5.E-02	*Tmem158*	NM_001002267
A_51_P220062	2.6	4.1.E-02	*Mmp15*	NM_008609
A_66_P103052	2.6	1.9.E-02	*N/A*	N/A
A_55_P2044054	2.6	2.6.E-02	*AA465934*	BB850827
A_55_P2036367	2.6	1.5.E-02	*Ipcef1*	NM_001170800
A_55_P2007470	2.6	1.0.E-02	*Pdgfa*	NM_008808
A_51_P226527	2.6	2.5.E-02	*Vmn1r85*	NM_145847
A_55_P1967231	2.6	9.5.E-03	*Stbd1*	NM_175096
A_55_P2002893	2.6	2.2.E-02	*Pfkfb1*	NM_008824
A_51_P181423	2.6	1.9.E-02	*Olfr807*	NM_146929
A_55_P2011922	2.6	6.1.E-03	*March9*	NM_001033262
A_55_P2020410	2.6	8.7.E-03	*Mapk8*	NM_016700
A_51_P193336	2.6	7.4.E-03	*Nucb2*	NM_016773
A_55_P2128195	2.6	4.4.E-02	*Pm20d2*	NM_001034867
A_55_P2098865	2.6	7.5.E-03	*Accn5*	NM_021370
A_55_P1961014	2.6	7.8.E-03	*Selenbp1*	NM_009150
A_51_P179258	2.6	4.1.E-02	*Kif26b*	NM_001161665
A_55_P2092194	2.6	4.0.E-02	*1700106N22Rik*	NM_028576
A_55_P2051566	2.6	1.4.E-02	*N/A*	CD768206
A_55_P2060303	2.6	7.5.E-03	*Mras*	NM_008624
A_55_P2074045	2.6	7.3.E-03	*N/A*	N/A
A_55_P2364971	2.6	3.8.E-02	*C77798*	C77798
A_55_P2018244	2.5	1.4.E-02	*Olfr1093*	NM_146366

A_55_P2016670	2.5	3.5.E-02	*Alpk2*	NM_001037294
A_52_P512575	2.5	1.1.E-02	*Hopx*	NM_175606
A_52_P130787	2.5	1.1.E-02	*Kcna6*	NM_013568
A_51_P461191	2.5	2.9.E-02	*Got1l1*	NM_029674
A_51_P222386	2.5	5.5.E-03	*Tbc1d10a*	NM_134023
A_55_P1960659	2.5	9.2.E-03	*Fer1l6*	XM_905719
A_55_P2137576	2.5	2.6.E-02	*Nphp3*	NM_028721
A_55_P1962680	2.5	1.4.E-02	*LOC10004023*	XM_001474135
A_55_P2087647	2.5	2.0.E-02	*Grhpr*	NM_080289
A_55_P2044803	2.5	2.8.E-02	*N/A*	AK085886
A_55_P2045549	2.5	8.8.E-03	*Dpy19l1*	NM_172920
A_55_P2012572	2.5	5.6.E-03	*Negr1*	NM_001039094
A_55_P2064486	2.5	2.7.E-02	*Trim58*	NM_001039047
A_55_P2139878	2.5	9.6.E-03	*N/A*	N/A
A_55_P2002376	2.5	9.5.E-03	*Srm*	NM_009272
A_55_P1979536	2.5	4.3.E-02	*Hras1*	NM_001130444
A_55_P2144090	2.5	3.8.E-02	*N/A*	N/A
A_55_P2015088	2.5	1.7.E-02	*N/A*	N/A
A_52_P233305	2.5	2.0.E-02	*Adamts12*	NM_175501
A_51_P514256	2.5	3.8.E-02	*Tubb2b*	NM_023716
A_55_P2085835	2.5	7.9.E-03	*5330417C22Ri*	NM_001033304
A_51_P310649	2.5	8.5.E-03	*Arsa*	NM_009713
A_55_P2008996	2.5	3.3.E-02	*Gm10034*	AK164250
A_51_P521052	2.5	7.2.E-03	*Ly6k*	NM_029627
A_55_P2158384	2.5	1.2.E-02	*Aanat*	NM_009591
A_55_P2062171	2.5	1.6.E-02	*Odf3*	AK005883
A_55_P2146789	2.5	1.8.E-02	*N/A*	N/A
A_55_P2107647	2.5	7.9.E-03	*N/A*	N/A
A_55_P2053556	2.5	7.7.E-03	*Alpk3*	NM_054085
A_51_P344552	2.5	2.7.E-02	*Irs4*	NM_010572
A_55_P1972039	2.5	1.0.E-02	*Nox4*	AK149398
A_55_P1987953	2.5	1.5.E-02	*Gm5938*	NM_001085534
A_52_P615401	2.5	1.6.E-02	*Tpra1*	NM_011906
A_51_P108629	2.5	1.7.E-02	*Prl3b1*	NM_008865
A_55_P2185367	2.5	2.8.E-02	*N/A*	XM_003084895
A_55_P2019428	2.5	7.9.E-03	*Htr1d*	NM_008309
A_51_P193000	2.5	1.0.E-02	*Bbs4*	NM_175325
A_51_P117794	2.5	1.3.E-02	*Bik*	NM_007546
A_52_P537545	2.5	4.5.E-02	*Smpd3*	NM_021491
A_66_P123683	2.5	1.5.E-02	*Obfc2a*	NM_028696
A_51_P259318	2.5	5.6.E-03	*Fbrs*	NM_010183

A_55_P1959891	2.5	1.1.E-02	*N/A*	N/A
A_51_P418725	2.5	1.4.E-02	*Plekhf1*	NM_024413
A_55_P1988373	2.5	2.1.E-02	*Mlf1ip*	NM_027973
A_55_P2019133	2.4	1.4.E-02	*Fmn1*	NM_001043322
A_55_P2179974	2.4	4.7.E-02	*Spsb1*	NM_029035
A_66_P128442	2.4	9.6.E-03	*Rimbp3*	NM_001033338
A_52_P361081	2.4	4.9.E-02	*Arhgef16*	NM_001112744
A_55_P2170514	2.4	2.8.E-02	*Sfn*	NM_018754
A_55_P1953194	2.4	4.0.E-02	*Uchl3*	AK050278
A_55_P2145506	2.4	3.0.E-02	*Flt3l*	U04807
A_55_P1989150	2.4	4.5.E-02	*Olfr294*	NM_001011750
A_51_P358316	2.4	6.8.E-03	*Chga*	NM_007693
A_51_P201308	2.4	1.8.E-02	*Moxd2*	NM_139296
A_55_P1987269	2.4	2.1.E-02	*Olfr1257*	NM_146982
A_55_P2057777	2.4	1.6.E-02	*Fgfr1*	NM_010206
A_55_P2032955	2.4	5.0.E-02	*Pabpn1l*	NM_001007462
A_55_P2142334	2.4	1.5.E-02	*Ly6h*	NM_011837
A_51_P129962	2.4	1.8.E-02	*5930434B04Ril*	AK083582
A_51_P386810	2.4	2.0.E-02	*Gmppb*	NM_177910
A_55_P2199463	2.4	3.4.E-02	*Tcfap2a*	NM_011547
A_52_P78875	2.4	1.5.E-02	*Phf12*	NM_174852
A_51_P143446	2.4	3.2.E-02	*Olfr1010*	NM_207149
A_55_P1967395	2.4	2.8.E-02	*N/A*	N/A
A_51_P134923	2.4	1.0.E-02	*Rnf148*	NM_027754
A_51_P413216	2.4	3.7.E-02	*Olfr1016*	NM_001011758
A_55_P2233547	2.4	8.7.E-03	*9130214F15Ril*	AK020279
A_52_P678393	2.4	1.8.E-02	*Slc22a14*	NM_001037749
A_55_P2318955	2.4	3.1.E-02	*4930417H01Ril*	AK144823
A_55_P2167482	2.4	8.1.E-03	*N/A*	N/A
A_55_P2090484	2.4	1.7.E-02	*Pde10a*	NM_011866
A_52_P436238	2.4	1.2.E-02	*Odc1*	NM_013614
A_55_P2154740	2.4	3.7.E-02	*N/A*	N/A
A_55_P2098494	2.4	2.2.E-02	*N/A*	BC058985
A_52_P273821	2.4	1.3.E-02	*Abhd5*	NM_026179
A_55_P2138422	2.4	1.3.E-02	*Obox1*	NM_027802
A_55_P1961320	2.4	4.2.E-02	*Tes*	NM_207176
A_51_P230537	2.4	4.3.E-02	*Ccdc114*	NM_001033243
A_55_P2059961	2.4	3.8.E-02	*N/A*	N/A
A_55_P1959072	2.4	1.0.E-02	*Clec2e*	NM_153506
A_55_P1976539	2.4	2.2.E-02	*Nup62cl*	NM_001081668
A_55_P2143494	2.4	2.9.E-02	*Doc2b*	NM_007873

A_51_P356842	2.4	2.1.E-02	*Olfr320*	NM_207230
A_52_P342159	2.4	7.8.E-03	*Nfatc4*	NM_023699
A_55_P2118866	2.4	1.9.E-02	*Cmah*	NM_001111110
A_51_P161554	2.4	6.4.E-03	*Arid3b*	NM_019689
A_55_P2146678	2.4	1.9.E-02	*N/A*	N/A
A_55_P1962124	2.4	9.9.E-03	*N/A*	N/A
A_55_P2096174	2.4	7.7.E-03	*LOC665792*	XM_003085443
A_55_P2074766	2.4	1.5.E-02	*Mug2*	NM_008646
A_55_P2087157	2.4	9.8.E-03	*N/A*	AK131168
A_66_P135700	2.4	9.3.E-03	*Cyp2d11*	NM_001104531
A_66_P133151	2.4	7.6.E-03	*N/A*	AK020435
A_55_P2149774	2.4	3.5.E-02	*Vasn*	NM_139307
A_55_P1972436	2.3	1.2.E-02	*Themis*	NM_178666
A_55_P2169784	2.3	1.1.E-02	*Plod1*	NM_011122
A_55_P2016976	2.3	3.2.E-02	*A530054K11Ri*	NM_183146
A_51_P262489	2.3	1.7.E-02	*Sst*	NM_009215
A_51_P413147	2.3	1.0.E-02	*Klk1b3*	NM_008693
A_51_P377812	2.3	2.2.E-02	*1700011E24Rik*	NM_029298
A_55_P1980550	2.3	1.5.E-02	*Kifc5c-ps*	XM_001000571
A_51_P240136	2.3	3.5.E-02	*Gng8*	NM_010320
A_55_P2000658	2.3	9.1.E-03	*Gm15645*	NR_033578
A_51_P219483	2.3	4.2.E-02	*Tsku*	NM_001024619
A_52_P368306	2.3	1.8.E-02	*Tmem100*	NM_026433
A_55_P2099952	2.3	2.2.E-02	*Car1*	NM_009799
A_52_P625215	2.3	1.5.E-02	*Wfikkn2*	NM_181819
A_55_P2423518	2.3	1.5.E-02	*B230369F24Ri*	AK050536
A_52_P52637	2.3	2.2.E-02	*N/A*	N/A
A_52_P578634	2.3	2.7.E-02	*Cela1*	NM_033612
A_55_P2044788	2.3	9.1.E-03	*N/A*	N/A
A_55_P1963041	2.3	1.7.E-02	*Gm4861*	NM_177665
A_55_P1977096	2.3	1.0.E-02	*Elk4*	NM_007923
A_55_P2036942	2.3	2.7.E-02	*Gm4535*	NM_001101644
A_55_P2105563	2.3	1.2.E-02	*1700104L18Rik*	AK007120
A_51_P335981	2.3	1.3.E-02	*Narf*	NM_026272
A_51_P408071	2.3	1.2.E-02	*Kntc1*	NM_001042421
A_55_P2137840	2.3	7.4.E-03	*4833442J19Rik*	NM_177101
A_51_P238183	2.3	1.2.E-02	*Etnk2*	NM_175443
A_55_P1991069	2.3	7.4.E-03	*Gm885*	NM_001033435
A_55_P1978777	2.3	9.5.E-03	*Zfp64*	NM_009564
A_52_P587738	2.3	2.4.E-02	*P2ry2*	NM_008773
A_55_P2131506	2.3	1.7.E-02	*N/A*	XR_105460

A_55_P1978885	2.3	1.9.E-02	*Gm10700*	AK172359
A_55_P1990648	2.3	7.8.E-03	*Ndufaf4*	NM_026742
A_52_P431859	2.3	1.7.E-02	*2010111I01Rik*	AK167772
A_55_P2112986	2.3	1.2.E-02	*Klk1b22*	NM_010114
A_52_P92472	2.3	1.6.E-02	*N/A*	N/A
A_55_P2083649	2.3	1.7.E-02	*Alas1*	NM_020559
A_66_P137048	2.3	2.4.E-02	*Men1*	NM_008583
A_55_P2095153	2.3	1.3.E-02	*Pdf*	NM_026513
A_55_P1998797	2.3	1.0.E-02	*N/A*	N/A
A_55_P2093168	2.3	7.9.E-03	*Rell2*	BC038500
A_55_P2046563	2.3	1.5.E-02	*Cym*	NM_001111143
A_55_P2165839	2.3	2.9.E-02	*Cebpe*	NM_207131
A_52_P190630	2.3	3.1.E-02	*1700011F03Rik*	NM_028825
A_51_P413545	2.3	1.3.E-02	*Txndc11*	NM_029582
A_52_P81693	2.3	1.0.E-02	*LOC10050259*	XM_003084652
A_55_P1956998	2.3	1.0.E-02	*Dis3l2*	NM_001172157
A_55_P1953984	2.3	3.9.E-02	*N/A*	AK144838
A_55_P2162432	2.3	1.6.E-02	*D630029K05Ri*	NR_027847
A_51_P277006	2.3	4.0.E-02	*Chst8*	NM_175140
A_55_P2172182	2.3	4.0.E-02	*Olfr1138*	NM_146639
A_55_P2160686	2.3	2.3.E-02	*Tsc22d1*	NM_207652
A_55_P2021953	2.3	2.1.E-02	*Olfr700*	NM_146600
A_55_P2005883	2.2	1.6.E-02	*Foxr1*	NM_001033469
A_55_P2009439	2.2	1.4.E-02	*N/A*	N/A
A_55_P2013751	2.2	1.9.E-02	*Tsga10*	NM_207228
A_51_P208870	2.2	3.0.E-02	*Zdhhc24*	NM_027476
A_55_P2136817	2.2	1.4.E-02	*Coro2b*	NM_175484
A_55_P2192961	2.2	9.1.E-03	*Trim30b*	NM_175648
A_55_P1964237	2.2	1.5.E-02	*4932418E24Rik*	NM_177841
A_55_P2126528	2.2	1.8.E-02	*N/A*	N/A
A_51_P440568	2.2	1.5.E-02	*Stk19*	NM_019442
A_55_P1960522	2.2	2.8.E-02	*N/A*	BY109983
A_55_P1992034	2.2	1.3.E-02	*Epm2a*	NM_010146
A_55_P1989991	2.2	2.0.E-02	*Fam149b*	AK031973
A_52_P18922	2.2	1.1.E-02	*Chid1*	NM_026522
A_55_P2148340	2.2	2.6.E-02	*N/A*	N/A
A_55_P2145029	2.2	2.6.E-02	*Gm826*	NM_001033411
A_51_P497379	2.2	9.9.E-03	*Olfr30*	NM_146878
A_55_P2026340	2.2	3.8.E-02	*Fmo5*	NM_001161765
A_55_P2051577	2.2	3.8.E-02	*N/A*	N/A
A_51_P192964	2.2	1.1.E-02	*Clec12b*	NM_001204223

A_51_P502152	2.2	1.9.E-02	*Slc19a1*	NM_031196
A_51_P448784	2.2	1.6.E-02	*Nrsn1*	NM_009513
A_55_P2115582	2.2	2.6.E-02	*Slc20a1*	NM_015747
A_55_P1958532	2.2	3.1.E-02	*Hr*	NM_021877
A_55_P2116744	2.2	1.3.E-02	*Xirp1*	NM_011724
A_55_P1964174	2.2	1.8.E-02	*Nme1*	NM_008704
A_55_P2065424	2.2	1.4.E-02	*Sstr2*	NM_009217
A_51_P313503	2.2	1.8.E-02	*Olfr577*	NM_147109
A_55_P2032543	2.2	1.1.E-02	*Cblc*	NM_001161844
A_52_P366047	2.2	1.5.E-02	*Rpp40*	NM_145938
A_66_P127160	2.2	1.7.E-02	*Eif2b3*	NM_001111277
A_55_P2043877	2.2	2.1.E-02	*2300003K06Ril*	NM_001195383
A_52_P333825	2.2	1.7.E-02	*Tas2r118*	NM_207022
A_55_P1992495	2.2	2.4.E-02	*Gpc4*	X83577
A_52_P339742	2.2	4.8.E-02	*Cyb5r3*	NM_029787
A_55_P2054437	2.2	1.3.E-02	*Ttpal*	NM_029512
A_55_P2143765	2.2	3.5.E-02	*Ugt1a6b*	NM_201410
A_55_P2161410	2.2	4.4.E-02	*Serpina3m*	NM_009253
A_52_P467232	2.2	1.0.E-02	*Il1rap*	NM_134103
A_52_P321751	2.2	4.1.E-02	*Narg2*	AK046121
A_55_P2049687	2.2	2.6.E-02	*Efna2*	NM_007909
A_55_P2096545	2.2	4.1.E-02	*R3hdml*	NM_001099331
A_65_P18756	2.2	3.1.E-02	*Klf17*	NM_029416
A_55_P1966094	2.2	1.7.E-02	*N/A*	N/A
A_55_P2020286	2.2	1.5.E-02	*Tmem179*	NM_178915
A_66_P112436	2.2	4.6.E-02	*Prkar2b*	NM_011158
A_51_P297968	2.2	2.9.E-02	*Pdia6*	NM_027959
A_51_P284716	2.2	1.9.E-02	*4930407I10Rik*	NM_001166475
A_55_P2008722	2.2	3.3.E-02	*Gm13157*	NM_001127189
A_66_P115959	2.2	1.4.E-02	*1700016H13Ril*	NM_001163550
A_55_P1974522	2.2	1.1.E-02	*A530099J19Ri*	NM_175688
A_55_P1995133	2.2	1.9.E-02	*N/A*	N/A
A_55_P1991960	2.2	2.8.E-02	*Nwd1*	NM_176940
A_52_P523368	2.1	1.4.E-02	*Psapl1*	NM_175249
A_55_P2205760	2.1	2.7.E-02	*C88045*	C86380
A_55_P2039289	2.1	1.3.E-02	*Hspb6*	NM_001012401
A_55_P1973352	2.1	1.1.E-02	*LOC10050391(*	XR_106152
A_55_P1982699	2.1	1.4.E-02	*Gm16442*	NM_001167148
A_55_P2021490	2.1	2.1.E-02	*Gm16386*	AK144317
A_55_P2009684	2.1	3.3.E-02	*Zmiz1*	AK169397
A_51_P164203	2.1	2.9.E-02	*Nme4*	NM_019731

A_52_P210909	2.1	4.0.E-02	*Pkd2l2*	NM_016927
A_55_P2041738	2.1	9.8.E-03	*Il15ra*	NM_008358
A_55_P2011286	2.1	2.6.E-02	*Hopx*	NM_175606
A_55_P2219581	2.1	1.4.E-02	*C76332*	C76332
A_55_P2099910	2.1	1.8.E-02	*F730043M19Ri*	NR_015602
A_55_P2126951	2.1	1.7.E-02	*Zfp467*	NM_001085417
A_55_P2168118	2.1	2.0.E-02	*4933406C10Ri*	AK041289
A_55_P2005611	2.1	1.3.E-02	*Olfr476*	NM_146924
A_66_P119350	2.1	1.1.E-02	*2310040G24Ri*	NR_040293
A_55_P2176270	2.1	1.9.E-02	*Gucy2e*	NM_008192
A_55_P2027852	2.1	1.9.E-02	*Ccl25*	NR_033527
A_55_P1979014	2.1	1.1.E-02	*Zfp648*	NM_001204908
A_51_P115229	2.1	1.6.E-02	*Vmn1r81*	NM_134210
A_55_P2040567	2.1	1.5.E-02	*N/A*	N/A
A_55_P2398995	2.1	1.3.E-02	*Lelp1*	NM_027042
A_55_P2409038	2.1	1.6.E-02	*7630403G23Ri*	AK020190
A_55_P2139703	2.1	1.8.E-02	*Krtap5-4*	NM_015809
A_51_P162098	2.1	1.5.E-02	*Ndufaf4*	NM_026742
A_52_P373694	2.1	4.3.E-02	*Jph4*	NM_177049
A_51_P392090	2.1	1.1.E-02	*Tmem19*	NM_133683
A_51_P214319	2.1	1.4.E-02	*Cox11*	NM_199008
A_51_P378699	2.1	1.1.E-02	*Sohlh2*	NM_028937
A_55_P2158181	2.1	1.9.E-02	*Olfr39*	NM_146825
A_55_P2165504	2.1	1.7.E-02	*N/A*	AK153764
A_52_P600822	2.1	1.8.E-02	*Prkcz*	NM_008860
A_51_P124126	2.1	2.6.E-02	*Cyp2d22*	NM_019823
A_55_P2162269	2.1	1.8.E-02	*Bri3*	NM_018772
A_55_P1967478	2.1	4.7.E-02	*Hbb-y*	NM_008221
A_55_P2067757	2.1	4.8.E-02	*Psg27*	NM_001037168
A_51_P501018	2.1	2.8.E-02	*Nek2*	NM_010892
A_55_P2414924	2.1	1.3.E-02	*Luc7l*	NM_028190
A_55_P1969131	2.1	3.6.E-02	*Cidec*	NM_178373
A_51_P219722	2.1	1.4.E-02	*2310021H06Ri*	NM_025990
A_51_P433778	2.1	1.6.E-02	*Tert*	NM_009354
A_55_P2162204	2.1	3.9.E-02	*Kctd15*	NM_146188
A_55_P2290388	2.1	1.3.E-02	*Rorb*	NM_001043354
A_52_P188593	2.1	2.8.E-02	*N/A*	AK049058
A_55_P2020331	2.1	2.8.E-02	*Camk1g*	NM_144817
A_51_P270005	2.1	2.9.E-02	*Lrguk*	NM_028886
A_55_P2035400	2.1	3.5.E-02	*Ripk4*	NM_023663
A_55_P2083233	2.1	4.2.E-02	*N/A*	N/A

A_55_P2066234	2.1	1.7.E-02	*Arfip2*	NM_029802
A_52_P252737	2.1	2.7.E-02	*Rangap1*	NM_011241
A_55_P2143976	2.1	3.8.E-02	*Kcnd1*	N/A
A_55_P2142878	2.1	1.2.E-02	*K230010J24Ril*	XM_484483
A_55_P2175245	2.0	1.9.E-02	*Tusc3*	NM_030254
A_51_P351137	2.0	2.6.E-02	*Tbce*	NM_178337
A_55_P1982034	2.0	2.7.E-02	*N/A*	N/A
A_66_P111322	2.0	2.8.E-02	*Gm5567*	NM_001004182
A_52_P295755	2.0	2.4.E-02	*A830005F24Ril*	XM_001473764
A_51_P158061	2.0	2.8.E-02	*Dalrd3*	NM_026378
A_52_P24631	2.0	3.6.E-02	*Azin1*	NM_018745
A_51_P408082	2.0	2.5.E-02	*Apoa1*	NM_009692
A_55_P2045658	2.0	2.3.E-02	*Nme6*	NM_018757
A_55_P2146590	2.0	3.2.E-02	*1810011O10Ril*	NM_026931
A_55_P2041713	2.0	2.8.E-02	*Gas2*	AK142903
A_51_P261204	2.0	2.6.E-02	*Foxi2*	NM_183193
A_55_P2125972	2.0	1.8.E-02	*Gorasp1*	NM_028976
A_55_P1964717	2.0	2.4.E-02	*Gm10398*	AK131726
A_55_P1970126	2.0	1.5.E-02	*Heatr5b*	NM_001081179
A_55_P2142724	2.0	4.6.E-02	*N/A*	N/A
A_66_P135194	2.0	1.5.E-02	*Yrdc*	NM_153566
A_55_P2167123	2.0	2.0.E-02	*Gm12839*	NR_033575
A_55_P2153391	2.0	1.9.E-02	*Palm2*	NM_172868
A_55_P2104119	2.0	2.2.E-02	*N/A*	N/A
A_55_P2283207	2.0	1.6.E-02	*Maz*	NM_010772
A_55_P1970062	2.0	4.0.E-02	*Igsf9b*	NM_001033323
A_55_P2075459	2.0	1.8.E-02	*Dtnb*	AJ003007
A_51_P358700	2.0	4.4.E-02	*Olfr1221*	NM_146902
A_55_P2006832	2.0	3.8.E-02	*Xkr4*	NM_001011874
A_55_P2107357	2.0	3.5.E-02	*LOC10004204!*	NR_004442
A_55_P2119962	2.0	3.0.E-02	*1110034G24Ril*	NM_028637
A_51_P428578	2.0	2.8.E-02	*Fam134b*	NM_025459
A_55_P1959985	2.0	1.9.E-02	*Alas1*	NM_020559
A_51_P449614	2.0	3.1.E-02	*1700006A11Ril*	NM_027939
A_52_P456158	2.0	1.9.E-02	*Fchsd1*	NM_175684
A_55_P2177233	2.0	3.0.E-02	*Abhd5*	NM_026179
A_55_P2050019	2.0	2.4.E-02	*Scml2*	NM_133194
A_51_P251357	2.0	2.8.E-02	*Ctps*	NM_016748
A_55_P2103831	2.0	1.4.E-02	*Gm6653*	XR_035409
A_51_P267783	2.0	2.6.E-02	*Il11*	NM_008350
A_55_P1970949	2.0	1.6.E-02	*N/A*	N/A

A_51_P387868	2.0	1.7.E-02	*Tekt4*	NM_027951
A_55_P1977303	2.0	3.1.E-02	*Pde11a*	AK050924
A_55_P2046744	2.0	3.9.E-02	*Cspg5*	NM_001166273
A_55_P2153923	2.0	1.6.E-02	*Slc17a5*	NM_172773
A_51_P403610	2.0	2.6.E-02	*Olfr1339*	NM_146852
A_51_P247542	2.0	2.0.E-02	*Ttll6*	NM_172799
A_55_P2032363	2.0	2.2.E-02	*Tomm40l*	NM_001037170
A_55_P1968808	2.0	2.7.E-02	*Wipi1*	NM_145940
A_55_P2130448	2.0	1.5.E-02	*N/A*	N/A
A_66_P125005	2.0	1.7.E-02	*Gm9648*	AK085591
A_55_P2026420	2.0	2.3.E-02	*Pou6f1*	NM_010127
A_52_P424462	2.0	2.0.E-02	*Ero1lb*	NM_026184
A_55_P2113078	2.0	2.6.E-02	*Rem1*	NM_009047
A_55_P2181216	2.0	2.6.E-02	*N/A*	N/A
A_51_P198835	2.0	2.2.E-02	*Dnajb9*	NM_013760
A_51_P484978	2.0	2.4.E-02	*Cblc*	NM_023224
A_51_P228171	2.0	2.3.E-02	*Cenpp*	NM_025495
A_55_P2107334	2.0	1.5.E-02	*Trim14*	NM_029077
A_55_P2413069	2.0	2.5.E-02	*N/A*	AK016955
A_55_P2412329	2.0	2.2.E-02	*4930581F22Rik*	AK157006
A_55_P1953411	2.0	1.7.E-02	*Efcab3*	AK162207
A_55_P1969560	2.0	1.7.E-02	*N/A*	N/A
A_55_P2036176	2.0	1.9.E-02	*Mcf2l*	NM_178076
A_55_P2257381	2.0	4.3.E-02	*LOC10050361*	XR_105802
A_55_P2100928	2.0	4.4.E-02	*Ptgds*	NM_008963
A_52_P583155	2.0	1.9.E-02	*Ykt6*	NM_019661
A_55_P2173199	2.0	3.2.E-02	*Vmn2r4*	NM_001104615
A_55_P2001494	2.0	1.6.E-02	*Kitl*	NM_013598
A_55_P2000210	2.0	4.3.E-02	*N/A*	AK141402
A_55_P2279777	2.0	2.8.E-02	*2810404M03Ri*	AK012985
A_52_P350111	2.0	2.4.E-02	*Cyth1*	NM_011180
A_55_P2127243	2.0	3.8.E-02	*Tpsb2*	NM_010781
A_55_P2130393	2.0	4.0.E-02	*Pnpla1*	NM_001034885
A_51_P385351	2.0	3.1.E-02	*Slc44a3*	NM_145394
A_55_P2072872	1.9	1.6.E-02	*Lpin2*	AK033662
A_51_P306047	1.9	2.3.E-02	*Sec13*	NM_024206
A_51_P385598	1.9	1.6.E-02	*Slc37a4*	NM_008063
A_55_P1995173	1.9	1.8.E-02	*Odc1*	NM_013614
A_55_P2059890	1.9	3.2.E-02	*Fdx1*	NM_007996
A_55_P2029252	1.9	1.3.E-02	*N/A*	N/A
A_55_P2096722	1.9	1.9.E-02	*N/A*	CF621068

A_55_P2151797	1.9	1.4.E-02	*Dis3l2*	NM_001172157
A_51_P291766	1.9	2.2.E-02	*Olfr521*	NM_146356
A_55_P1978651	1.9	2.2.E-02	*Wasf3*	NM_145155
A_51_P240801	1.9	1.6.E-02	*Tmem173*	NM_028261
A_55_P2171493	1.9	2.4.E-02	*BC030867*	NM_153544
A_51_P163578	1.9	2.0.E-02	*Ugt2b35*	NM_172881
A_55_P2064635	1.9	4.7.E-02	*N/A*	N/A
A_55_P2184385	1.9	2.5.E-02	*N/A*	N/A
A_55_P2136289	1.9	4.2.E-02	*Ripk4*	BC057871
A_55_P2422650	1.9	2.9.E-02	*5031425E22Rik*	AK017143
A_55_P2181348	1.9	2.9.E-02	*N/A*	BY742470
A_51_P161691	1.9	1.8.E-02	*Coq7*	NM_009940
A_52_P508277	1.9	1.4.E-02	*Pramel*	XM_885719
A_55_P1956624	1.9	1.8.E-02	*Gm14431*	NM_001177406
A_52_P520408	1.9	3.0.E-02	*Serp1*	NM_030685
A_66_P111594	1.9	3.3.E-02	*V1ra8*	NM_053223
A_55_P1982524	1.9	2.9.E-02	*Cyhr1*	NM_019396
A_55_P2025073	1.9	2.2.E-02	*Csf1r*	NM_001037859
A_51_P429472	1.9	2.1.E-02	*Fam188a*	NM_024185
A_55_P1966874	1.9	3.7.E-02	*Rtbdn*	NM_144929
A_55_P2130109	1.9	1.8.E-02	*Nmt2*	NM_008708
A_51_P382805	1.9	2.4.E-02	*Zpbp2*	NM_027061
A_55_P2116889	1.9	2.0.E-02	*Hax1*	NM_011826
A_55_P2010858	1.9	2.4.E-02	*Mrps23*	NM_024174
A_55_P1978825	1.9	2.8.E-02	*Gm10556*	XR_107098
A_55_P2029846	1.9	2.4.E-02	*BC031353*	NM_001113283
A_51_P208603	1.9	2.7.E-02	*Dio2*	NM_010050
A_66_P108451	1.9	1.7.E-02	*N/A*	N/A
A_55_P2082806	1.9	4.4.E-02	*Trib1*	NM_144549
A_51_P490678	1.9	3.4.E-02	*Trim68*	NM_198012
A_55_P1967350	1.9	3.3.E-02	*Ugt2b35*	NM_172881
A_55_P2341950	1.9	4.2.E-02	*Crebzf*	NM_145151
A_55_P1964363	1.9	1.6.E-02	*Kctd14*	NM_001012434
A_55_P2002743	1.9	4.4.E-02	*N/A*	N/A
A_51_P438555	1.9	4.4.E-02	*4930412D23Rik*	XM_001473476
A_55_P2109337	1.9	3.1.E-02	*Gba2*	NM_172692
A_51_P421804	1.9	1.6.E-02	*Timm9*	NM_013896
A_55_P2157751	1.9	3.1.E-02	*Stk35*	NM_183262
A_52_P473419	1.9	4.2.E-02	*Epb4.1l4a*	NM_013512
A_55_P2045697	1.9	2.7.E-02	*Sirt7*	NM_153056
A_55_P1961700	1.9	1.7.E-02	*Ush2a*	DQ463440

A_51_P319379	1.9	2.4.E-02	*Tmx4*	NM_029148
A_55_P2106514	1.9	1.7.E-02	*Scn2a1*	NM_001099298
A_66_P138053	1.9	3.8.E-02	*Gm9917*	XR_105317
A_51_P271503	1.9	3.4.E-02	*Il1r1*	NM_008362
A_55_P2210315	1.9	2.0.E-02	*Get4*	NM_026269
A_51_P362959	1.9	3.1.E-02	*Fbxo36*	NM_025386
A_55_P2081035	1.9	2.6.E-02	*Gpr25*	NM_001101516
A_52_P56636	1.9	1.9.E-02	*Chia*	NM_023186
A_55_P2035102	1.9	1.7.E-02	*Odc1*	BC083122
A_55_P1964752	1.9	3.6.E-02	*Slc23a3*	NM_194333
A_51_P377376	1.9	3.5.E-02	*Gnl3*	NM_153547
A_55_P2027392	1.9	2.9.E-02	*Gpr146*	NM_030258
A_55_P2053251	1.9	3.1.E-02	*Gabrg3*	NM_008074
A_51_P187901	1.9	3.2.E-02	*Nop56*	NM_024193
A_52_P538470	1.9	3.0.E-02	*Spats2l*	NM_144882
A_55_P2039530	1.9	3.9.E-02	*Gm10724*	XM_003086271
A_55_P2066927	1.9	3.1.E-02	*Galt*	AK088379
A_52_P322421	1.9	2.9.E-02	*Mpzl2*	NM_007962
A_51_P386270	1.9	2.5.E-02	*Cyp2d12*	NM_201360
A_55_P2298158	1.9	3.2.E-02	*D8Ertd620e*	BB618401
A_55_P2019964	1.9	2.5.E-02	*Lancl1*	NM_001190985
A_55_P2170978	1.9	2.2.E-02	*Ankrd13d*	NM_026720
A_55_P2177614	1.9	2.1.E-02	*Srsf3*	NM_013663
A_55_P2044942	1.9	3.8.E-02	*N/A*	N/A
A_51_P295034	1.9	1.9.E-02	*Klk1b4*	NM_010915
A_55_P2092125	1.9	2.5.E-02	*N/A*	N/A
A_51_P318856	1.9	2.1.E-02	*Glyat*	NM_145935
A_55_P2067076	1.9	2.9.E-02	*N/A*	AK135112
A_55_P1992934	1.9	3.5.E-02	*N/A*	N/A
A_55_P2131428	1.9	2.4.E-02	*Tlr5*	NM_016928
A_55_P1994550	1.9	1.7.E-02	*Shank1*	NM_001034115
A_51_P349281	1.9	2.7.E-02	*Nckap5l*	NM_001001884
A_55_P2067116	1.9	1.7.E-02	*Dclre1c*	NM_175683
A_66_P113708	1.9	3.6.E-02	*Nespas*	NR_002846
A_66_P106711	1.9	3.5.E-02	*Hhipl1*	NM_001044380
A_51_P253984	1.9	1.8.E-02	*Pcp4*	NM_008791
A_55_P2250164	1.9	2.9.E-02	*Pcdhgc5*	NM_033583
A_55_P2158866	1.9	4.1.E-02	*Nme6*	NM_018757
A_55_P2183015	1.8	2.0.E-02	*P2rx4*	NM_011026
A_52_P26953	1.8	3.8.E-02	*Ergic2*	NM_026355
A_55_P1978471	1.8	1.9.E-02	*N/A*	N/A

A_66_P130560	1.8	1.7.E-02	*N/A*	N/A
A_66_P114985	1.8	3.0.E-02	*N/A*	N/A
A_55_P2022258	1.8	4.7.E-02	*N/A*	AF012163
A_51_P414889	1.8	2.5.E-02	*Ifi35*	NM_027320
A_55_P1959595	1.8	3.2.E-02	*Fmo5*	NM_001161765
A_55_P2076941	1.8	3.0.E-02	*Lrr1*	NM_001081406
A_51_P510939	1.8	2.4.E-02	*Chchd7*	NM_181391
A_51_P391616	1.8	2.9.E-02	*Agxt2l1*	NM_027907
A_55_P2160243	1.8	4.0.E-02	*N/A*	N/A
A_52_P457339	1.8	2.6.E-02	*Gm1965*	NM_001033491
A_55_P2033987	1.8	3.0.E-02	*N/A*	N/A
A_52_P207964	1.8	3.4.E-02	*Mrps18b*	NM_025878
A_51_P245414	1.8	2.9.E-02	*Klk1*	NM_010639
A_51_P353543	1.8	3.2.E-02	*Magee2*	NM_053206
A_55_P1992174	1.8	2.3.E-02	*Tars2*	NM_001163619
A_55_P2095844	1.8	2.6.E-02	*Kalrn*	NM_001164268
A_55_P2017343	1.8	4.2.E-02	*Olfr767*	NM_146318
A_55_P2015267	1.8	3.1.E-02	*N/A*	N/A
A_52_P317040	1.8	2.6.E-02	*Edem2*	NM_145537
A_55_P2017689	1.8	2.2.E-02	*Gprc5c*	NM_147217
A_55_P1952775	1.8	4.8.E-02	*Olfr559*	NM_147112
A_55_P2159791	1.8	4.0.E-02	*Serpinb3d*	NM_201376
A_55_P2078460	1.8	4.9.E-02	*N/A*	N/A
A_55_P2075268	1.8	2.8.E-02	*Tmem216*	NM_026798
A_55_P2001481	1.8	2.9.E-02	*Pde6c*	NM_033614
A_55_P1976829	1.8	2.9.E-02	*Fam165b*	N/A
A_55_P2068486	1.8	1.9.E-02	*Arfgap1*	NM_001177706
A_51_P367060	1.8	1.9.E-02	*Ifrd1*	NM_013562
A_51_P120066	1.8	2.5.E-02	*9330151L19Rik*	NR_033222
A_55_P2026894	1.8	2.8.E-02	*N/A*	N/A
A_51_P250445	1.8	5.0.E-02	*Zfp276*	NM_020497
A_52_P164709	1.8	2.0.E-02	*Poc1a*	NM_027354
A_52_P587642	1.8	4.5.E-02	*Ddx19a*	NM_007916
A_55_P2215880	1.8	3.1.E-02	*Ephb2*	NM_010142
A_55_P2053491	1.8	2.3.E-02	*Pdia6*	NM_027959
A_51_P342307	1.8	3.0.E-02	*Olfr732*	NM_146665
A_55_P2244971	1.8	3.1.E-02	*4930404O17Ri*	BY714618
A_55_P1975832	1.8	2.3.E-02	*1810009N02Ri*	NM_026939
A_55_P1973813	1.8	3.6.E-02	*N/A*	N/A
A_55_P2079324	1.8	3.9.E-02	*Sephs2*	NM_009266
A_55_P2109752	1.8	3.8.E-02	*Nans*	NM_053179

A_52_P237792	1.8	1.9.E-02	*Slc19a1*	NM_031196
A_66_P101703	1.8	2.0.E-02	*Krtap5-5*	NM_001037822
A_55_P2183032	1.8	3.9.E-02	*Zfp787*	AK018745
A_52_P185343	1.8	2.6.E-02	*Gna13*	AK011851
A_51_P269320	1.8	4.0.E-02	*Adsl*	NM_009634
A_55_P2106201	1.8	2.3.E-02	*N/A*	N/A
A_55_P2046055	1.8	3.2.E-02	*Arhgap42*	AK018520
A_51_P330428	1.8	3.8.E-02	*Eif4ebp1*	NM_007918
A_55_P2000930	1.8	2.9.E-02	*Ccnyl1*	NM_001097644
A_52_P148658	1.8	2.5.E-02	*Mettl22*	NM_146247
A_52_P348720	1.8	3.3.E-02	*Rhobtb3*	NM_028493
A_55_P2020188	1.8	4.3.E-02	*N/A*	N/A
A_55_P1980836	1.8	4.3.E-02	*N/A*	EF154513
A_52_P108850	1.8	3.1.E-02	*St8sia1*	NM_011374
A_55_P2023352	1.8	2.6.E-02	*N/A*	N/A
A_55_P2138366	1.8	3.3.E-02	*Xpo4*	NM_020506
A_52_P404329	1.8	5.0.E-02	*Saa4*	NM_011316
A_55_P1952925	1.8	2.2.E-02	*AI854703*	NR_027236
A_51_P118223	1.8	3.5.E-02	*Gm1943*	NR_002928
A_55_P2022049	1.8	2.9.E-02	*Klf15*	NM_023184
A_55_P2077048	1.8	4.9.E-02	*Itih5*	NM_172471
A_55_P2031939	1.8	2.8.E-02	*Aqp4*	NM_009700
A_55_P1994190	1.8	3.6.E-02	*Heph*	NM_181273
A_55_P1988102	1.8	2.4.E-02	*Hmbs*	NM_013551
A_51_P465292	1.8	4.0.E-02	*Hnmt*	NM_080462
A_55_P2145606	1.8	3.5.E-02	*Kmo*	NM_133809
A_55_P2024763	1.8	5.0.E-02	*Lama3*	NM_010680
A_55_P2068148	1.8	2.8.E-02	*Pias1*	NM_019663
A_55_P2370931	1.8	3.6.E-02	*AU017674*	CK334688
A_52_P562267	1.8	2.2.E-02	*9130409I23Rik*	NM_001033819
A_52_P188678	1.8	3.9.E-02	*Pvrl2*	NM_001159724
A_55_P1966690	1.8	3.3.E-02	*Cyp2e1*	NM_021282
A_51_P252410	1.8	2.6.E-02	*Cope*	NM_021538
A_55_P2045048	1.8	4.3.E-02	*N/A*	N/A
A_52_P458697	1.8	2.3.E-02	*Higd1a*	NM_019814
A_55_P2115851	1.8	3.6.E-02	*9930013L23Rik*	NM_030728
A_55_P2127692	1.8	2.8.E-02	*Timm50*	NM_025616
A_55_P2093023	1.8	3.8.E-02	*Xrcc6bp1*	NM_026858
A_52_P199299	1.8	3.1.E-02	*Urgcp*	NM_001077661
A_66_P130035	1.8	2.1.E-02	*Klk1b24*	NM_010643
A_51_P235139	1.8	4.2.E-02	*Kbtbd5*	NM_028202

A_55_P2073945	1.8	2.6.E-02	*Eif2b3*	NM_001111277
A_55_P2100866	1.8	4.6.E-02	*N/A*	N/A
A_55_P2115732	1.8	2.3.E-02	*Kif1c*	NM_153103
A_51_P369311	1.8	3.1.E-02	*Nox4*	NM_015760
A_52_P8903	1.8	3.2.E-02	*D230037D09Ri*	NM_177140
A_51_P519189	1.8	3.3.E-02	*Eif3i*	NM_018799
A_55_P2153221	1.8	3.1.E-02	*N/A*	N/A
A_55_P1952882	1.8	2.4.E-02	*Cyp4f18*	AF233647
A_55_P2024669	1.8	2.8.E-02	*Myo6*	NM_001039546
A_52_P72654	1.8	2.7.E-02	*Slc25a17*	NM_011399
A_55_P2161640	1.8	3.5.E-02	*Slc38a1*	NM_134086
A_55_P1995417	1.8	4.4.E-02	*1810031K17Ril*	NM_026977
A_55_P1988083	1.7	2.8.E-02	*Prc1*	NM_145150
A_55_P1963201	1.7	2.1.E-02	*Chchd7*	NM_181391
A_52_P223571	1.7	2.0.E-02	*Mlec*	NM_175403
A_55_P1968195	1.7	3.4.E-02	*Cyp2d13*	NR_003552
A_55_P1979463	1.7	3.3.E-02	*Prdx5*	NM_012021
A_55_P1981719	1.7	4.0.E-02	*Rreb1*	NR_033615
A_55_P2229645	1.7	4.7.E-02	*C77847*	BG079317
A_51_P276063	1.7	2.6.E-02	*Phyh*	NM_010726
A_51_P506328	1.7	3.2.E-02	*Cyp2j6*	NM_010008
A_55_P2043622	1.7	2.3.E-02	*Grhpr*	AK168351
A_55_P1985758	1.7	4.1.E-02	*Psmd9*	NM_026000
A_51_P281608	1.7	2.3.E-02	*Olfr609*	NM_147082
A_55_P2067798	1.7	4.2.E-02	*Vmn1r59*	NM_207543
A_55_P1990067	1.7	4.2.E-02	*N/A*	N/A
A_52_P96552	1.7	2.7.E-02	*Vkorc1l1*	NM_027121
A_55_P2087061	1.7	2.6.E-02	*Gm5151*	NM_001101506
A_51_P205385	1.7	2.8.E-02	*Uox*	NM_009474
A_55_P2065577	1.7	3.9.E-02	*N/A*	AK020097
A_55_P2154387	1.7	3.6.E-02	*Bmp4*	NM_007554
A_51_P150521	1.7	3.9.E-02	*Mgat2*	NM_146035
A_51_P301964	1.7	2.8.E-02	*Arl6ip1*	NM_019419
A_52_P376106	1.7	4.4.E-02	*Slc30a9*	NM_178651
A_55_P2105406	1.7	4.7.E-02	*N/A*	XM_003085830
A_51_P128075	1.7	4.7.E-02	*1700008P20Ril*	NM_001163810
A_55_P1957618	1.7	3.7.E-02	*Ak2*	NM_001033966
A_55_P2002933	1.7	2.4.E-02	*Klk1b5*	NM_008456
A_51_P254234	1.7	2.7.E-02	*Chchd4*	NM_133928
A_51_P125050	1.7	3.5.E-02	*Wdr74*	NM_134139
A_51_P227345	1.7	2.5.E-02	*Dpep1*	NM_007876

A_51_P108489	1.7	2.5.E-02	*Gtl3*	NM_008187
A_55_P1972104	1.7	2.6.E-02	*Acsm3*	NM_212441
A_51_P243930	1.7	4.2.E-02	*Qrsl1*	NM_001081054
A_55_P2093720	1.7	3.2.E-02	*Dnajc5*	NM_016775
A_55_P2043682	1.7	4.8.E-02	*Ubac1*	BC021811
A_55_P2145617	1.7	3.6.E-02	*Krt80*	NM_028770
A_55_P2088715	1.7	3.9.E-02	*Deb1*	NM_026794
A_55_P1998539	1.7	3.1.E-02	*C1qtnf1*	NM_001204129
A_52_P378900	1.7	4.8.E-02	*Lpcat2b*	NM_027599
A_55_P2029630	1.7	2.6.E-02	*Mpdu1*	NM_011900
A_55_P2002068	1.7	3.9.E-02	*Gm10510*	AK145347
A_55_P2102621	1.7	3.3.E-02	*Eaf2*	NM_001113401
A_51_P402458	1.7	2.7.E-02	*Anxa7*	NM_009674
A_55_P2186282	1.7	3.8.E-02	*Agxt2*	NM_001031851
A_51_P468762	1.7	2.9.E-02	*Alkbh6*	NM_198027
A_66_P129619	1.7	3.6.E-02	*Gm2011*	NR_038067
A_55_P2027678	1.7	4.4.E-02	*N/A*	AK006745
A_55_P2114110	1.7	3.0.E-02	*Cadm4*	NM_153112
A_66_P100021	1.7	2.7.E-02	*Ncaph2*	NM_001115132
A_55_P2038479	1.7	4.1.E-02	*Pddc1*	NM_172116
A_55_P2100120	1.7	2.8.E-02	*Nme1*	NM_008704
A_51_P160372	1.7	2.9.E-02	*Wbp1*	NM_016757
A_55_P1956873	1.7	2.6.E-02	*Timm17a*	NM_011590
A_51_P102438	1.7	4.7.E-02	*Ugt2b36*	NM_001029867
A_55_P2120155	1.7	3.6.E-02	*Ccdc88b*	NM_001081291
A_51_P255329	1.7	2.9.E-02	*Riok2*	NM_025934
A_55_P2425342	1.7	4.5.E-02	*4932443L11Rik*	XR_105182
A_51_P193716	1.7	4.3.E-02	*Supv3l1*	NM_181423
A_55_P2081630	1.7	2.6.E-02	*Gpa33*	NM_021610
A_55_P1990740	1.7	3.7.E-02	*N/A*	N/A
A_55_P2147220	1.7	3.2.E-02	*Ak2*	NM_016895
A_52_P469277	1.7	5.0.E-02	*N/A*	AF087578
A_55_P2187043	1.7	4.1.E-02	*Tpm1*	NM_001164248
A_51_P492125	1.7	2.7.E-02	*Ciapin1*	NM_134141
A_52_P365768	1.7	2.6.E-02	*Nsun2*	NM_145354
A_55_P2045535	1.7	3.4.E-02	*6330545A04Rik*	N/A
A_55_P2031676	1.7	3.1.E-02	*Gstm6*	NM_008184
A_51_P234544	1.7	3.4.E-02	*Azin1*	NM_018745
A_51_P155303	1.7	4.2.E-02	*Rbfa*	NM_199197
A_55_P2052481	1.7	4.4.E-02	*Olfr1130*	NM_146838
A_55_P2081981	1.7	3.5.E-02	*N/A*	AK046704

A_55_P2040280	1.7	3.1.E-02	*Dhps*	NM_001039514
A_52_P303077	1.7	5.0.E-02	*Rogdi*	NM_133185
A_55_P1960208	1.7	2.5.E-02	*Vcan*	NM_172955
A_55_P2112245	1.7	4.9.E-02	*Ccdc13*	AK038868
A_51_P435922	1.7	4.1.E-02	*Rsph9*	NM_029338
A_55_P2096772	1.7	4.7.E-02	*Zfp11*	NM_172462
A_55_P1963529	1.7	3.9.E-02	*Abcb10*	NM_019552
A_51_P395473	1.7	3.6.E-02	*Tenc1*	NM_153533
A_55_P2019217	1.7	3.7.E-02	*Gm2695*	XR_106466
A_55_P1987675	1.7	3.2.E-02	*N/A*	N/A
A_52_P159885	1.7	3.9.E-02	*Ddx49*	NM_001024922
A_55_P2007233	1.7	4.3.E-02	*A530021J07Ri*	XR_035179
A_55_P2072801	1.7	4.6.E-02	*Lypd5*	NM_029806
A_51_P204702	1.7	3.3.E-02	*Olfr493*	NM_146310
A_52_P100199	1.7	3.3.E-02	*Abhd10*	NM_172511
A_55_P2176320	1.7	4.1.E-02	*Pgc*	NM_025973
A_52_P302345	1.7	3.3.E-02	*Cyp4v3*	NM_133969
A_51_P235705	1.7	4.2.E-02	*Tsfm*	NM_025537
A_51_P480904	1.7	3.8.E-02	*Blmh*	NM_178645
A_55_P2166349	1.7	3.0.E-02	*N/A*	BC024335
A_55_P2053616	1.7	2.9.E-02	*N/A*	N/A
A_51_P376407	1.7	3.5.E-02	*D9Ertd402e*	NM_001013405
A_52_P469381	1.7	3.6.E-02	*Comtd1*	NM_026965
A_51_P309988	1.7	3.7.E-02	*Gprc5c*	NM_147217
A_55_P2168736	1.7	2.6.E-02	*Relb*	NM_009046
A_55_P2019014	1.6	4.4.E-02	*Tmem120b*	NM_001039723
A_51_P422893	1.6	3.5.E-02	*Tmem14a*	NM_029398
A_51_P116687	1.6	4.3.E-02	*1700010I14Rik*	NM_025851
A_55_P2029498	1.6	4.5.E-02	*Osbpl1a*	NM_207530
A_52_P45797	1.6	2.8.E-02	*Rg9mtd2*	NM_175389
A_55_P2026639	1.6	3.6.E-02	*Gm11564*	NM_001100614
A_51_P440790	1.6	3.7.E-02	*Dpp7*	NM_031843
A_55_P2005956	1.6	4.7.E-02	*Egfbp2*	NM_010115
A_55_P2127288	1.6	3.5.E-02	*Spsb1*	N/A
A_55_P2051791	1.6	3.1.E-02	*Cbr4*	NM_145595
A_55_P2072468	1.6	3.8.E-02	*Sirt6*	NM_181586
A_55_P2012689	1.6	4.0.E-02	*Stk16*	NM_011494
A_55_P2043352	1.6	4.0.E-02	*LOC10050398*	XM_003085022
A_55_P2073795	1.6	3.7.E-02	*Mecr*	NM_025297
A_55_P2159061	1.6	4.2.E-02	*Taar8a*	NM_001010830
A_55_P2334424	1.6	4.9.E-02	*D630024D03Ri*	AK158188

A_51_P465809	1.6	4.1.E-02	*Slc30a9*	NM_178651
A_55_P2076846	1.6	3.0.E-02	*Tead2*	NM_011565
A_66_P120605	1.6	4.7.E-02	*Fbxo34*	NM_001146085
A_55_P2187053	1.6	4.7.E-02	*Tpm1*	NM_001164255
A_52_P217437	1.6	4.2.E-02	*Htra3*	NM_001042615
A_52_P618187	1.6	4.0.E-02	*Mrs2*	NM_001013389
A_55_P2126363	1.6	4.7.E-02	*Wnt10b*	NM_011718
A_55_P2169218	1.6	4.0.E-02	*N/A*	N/A
A_51_P160744	1.6	4.5.E-02	*Ndufb3*	NM_025597
A_51_P394115	1.6	4.8.E-02	*Aadac*	NM_023383
A_55_P2063041	1.6	4.8.E-02	*Slmo2*	NM_025531
A_55_P2074811	1.6	3.4.E-02	*Zfp609*	NM_172536
A_52_P230904	1.6	2.9.E-02	*Wdr18*	NM_175450
A_51_P305547	1.6	4.8.E-02	*Snai2*	NM_011415
A_55_P1978847	1.6	4.6.E-02	*Vmn1r183*	NM_203489
A_52_P24986	1.6	4.2.E-02	*Agpat6*	NM_018743
A_51_P490171	1.6	3.8.E-02	*Nubp2*	NM_011956
A_55_P2003216	1.6	4.9.E-02	*Igsf5*	NM_001177886
A_55_P1976689	1.6	3.7.E-02	*Snrnp35*	NM_029532
A_66_P121117	1.6	4.2.E-02	*Serpina1d*	NM_009246
A_51_P109888	1.6	3.7.E-02	*Coro1b*	NM_011778
A_52_P117313	1.6	3.4.E-02	*Azin1*	NM_018745
A_55_P2043684	1.6	4.5.E-02	*Ubac1*	NM_133835
A_51_P109421	1.6	4.5.E-02	*Eif2d*	NM_010709
A_51_P468329	1.6	4.3.E-02	*Psmb7*	NM_011187
A_55_P1985458	1.6	4.0.E-02	*Susd3*	AK013276
A_52_P1003335	1.6	3.0.E-02	*5830473C10Ri*	XM_886735
A_51_P357561	1.6	4.3.E-02	*Fbxw9*	NM_026791
A_55_P2044433	1.6	4.9.E-02	*LOC10050401*	XR_108069
A_55_P2146650	1.6	4.5.E-02	*N/A*	N/A
A_55_P2429714	1.6	3.3.E-02	*4930455C21Ri*	NM_024273
A_52_P10781	1.6	4.6.E-02	*Lman1*	NM_001172062
A_51_P311096	1.6	4.5.E-02	*2410003K15Ri*	NM_029353
A_55_P2073642	1.6	4.3.E-02	*1600014C10Ri*	NM_001085385
A_55_P2302000	1.6	4.0.E-02	*4930455J16Ri*	AK030052
A_55_P2248556	1.6	3.8.E-02	*6530402F18Ri*	AK220426
A_52_P343343	1.6	3.7.E-02	*Rpl7l1*	NM_025433
A_52_P52272	1.6	4.7.E-02	*Zfp846*	NM_172919
A_55_P2009875	1.6	4.5.E-02	*N/A*	N/A
A_55_P1953972	1.6	4.9.E-02	*Pdhb*	NM_024221
A_66_P115056	1.6	4.5.E-02	*Gpn1*	NM_133756

A_55_P2011341	1.6	3.8.E-02	*N/A*	N/A
A_51_P435068	1.6	4.1.E-02	*Acadsb*	NM_025826
A_55_P2137521	1.6	4.5.E-02	*Nmral1*	NM_026393
A_55_P2021455	1.6	5.0.E-02	*N/A*	N/A
A_51_P520106	1.6	4.6.E-02	*Adprhl2*	NM_133883
A_55_P1988070	1.6	4.4.E-02	*Eef1g*	NM_026007
A_55_P1984601	1.6	4.9.E-02	*Vapb*	NM_019806
A_52_P215829	1.6	5.0.E-02	*Alg13*	NR_037145
A_55_P2031288	1.6	3.6.E-02	*Abcg5*	NM_031884
A_55_P2003211	1.6	4.9.E-02	*Igsf5*	NM_001177887
A_55_P2013756	1.6	4.5.E-02	*Sult5a1*	XR_108050
A_55_P2111429	1.6	3.4.E-02	*Btf3l4*	NM_027453
A_52_P180335	1.6	4.3.E-02	*Mrpl28*	NM_024227
A_55_P2094362	1.6	4.8.E-02	*Erp29*	NM_026129
A_51_P419759	1.6	4.3.E-02	*Tha1*	NM_027919
A_51_P483311	1.6	4.8.E-02	*Mpv17l2*	NM_183170
A_51_P504522	1.6	5.0.E-02	*Derl1*	NM_024207
A_55_P2020084	1.6	3.6.E-02	*N/A*	N/A
A_55_P2117319	1.6	5.0.E-02	*Ntan1*	NM_010946
A_55_P1963389	1.6	4.2.E-02	*N/A*	N/A
A_55_P1979491	1.6	3.7.E-02	*Cd28*	NM_007642
A_51_P407193	1.6	4.8.E-02	*Clp1*	NM_133840
A_55_P2022128	1.6	4.7.E-02	*Cntnap1*	NM_016782
A_52_P676063	1.6	4.7.E-02	*Tmem126b*	NM_026734
A_55_P2028064	1.6	4.1.E-02	*Kdm4b*	NM_172132
A_55_P2037657	1.6	4.9.E-02	*Gm10427*	AK149283
A_55_P2002557	1.6	4.4.E-02	*Srebf1*	NM_011480
A_55_P2114785	1.6	4.0.E-02	*N/A*	N/A
A_55_P2025006	1.6	4.9.E-02	*Klk1b26*	NM_010644
A_55_P2150053	1.6	4.7.E-02	*Yipf5*	NM_023311
A_55_P2048928	1.6	4.1.E-02	*Cbfb*	NM_001161458
A_55_P2000254	1.6	4.4.E-02	*Rundc2a*	NM_001163498
A_55_P1992839	1.6	4.5.E-02	*Dsn1*	NM_025853
A_55_P2006554	1.6	4.3.E-02	*Eif4e2*	NM_001039169
A_51_P462556	1.6	4.2.E-02	*Gm10658*	XR_105260
A_55_P2317665	1.6	4.7.E-02	*E2f1*	NM_007891
A_55_P2056995	1.5	4.6.E-02	*Gcgr*	NM_008101
A_66_P123787	1.5	4.4.E-02	*Lars2*	NM_153168
A_51_P411297	1.5	4.4.E-02	*Nup50*	NM_016714
A_51_P362554	1.5	4.9.E-02	*Olfr1307*	NM_001011787
A_55_P1964302	1.5	4.7.E-02	*Timm8a1*	NM_013898

A_51_P125567	1.5	4.7.E-02	*Mettl13*	NM_144877
A_55_P2007771	1.5	5.0.E-02	*Catsper2*	NM_153075
A_66_P132037	1.5	4.8.E-02	*Jmjd4*	NM_178659
A_52_P263201	1.5	4.8.E-02	*Tmem33*	NM_030108
A_52_P139097	1.5	4.9.E-02	*N/A*	N/A
